# Time–frequency co-movement and risk connectedness among cryptocurrencies: new evidence from the higher-order moments before and during the COVID-19 pandemic

**DOI:** 10.1186/s40854-022-00395-w

**Published:** 2022-09-30

**Authors:** Jinxin Cui, Aktham Maghyereh

**Affiliations:** 1 Collaborative Innovation Center of Statistical Data Engineering Technology and Application, Zhejiang Gongshang University, Hangzhou, 310018 People’s Republic of China; 2 International Business School, Zhejiang Gongshang University, Hangzhou, 310018 People’s Republic of China; 3 Department of Accounting and Finance, United Arab Emirates University, Abu Dhabi, United Arab Emirates

**Keywords:** Cryptocurrencies, Higher-order realized moments and jumps, Time–frequency comovements and connectedness, High-frequency data, COVID-19 pandemic, C51, C58, G20, G15, G40

## Abstract

Analyzing comovements and connectedness is critical for providing significant implications for crypto-portfolio risk management. However, most existing research focuses on the lower-order moment nexus (i.e. the return and volatility interactions). For the first time, this study investigates the higher-order moment comovements and risk connectedness among cryptocurrencies before and during the COVID-19 pandemic in both the time and frequency domains. We combine the realized moment measures and wavelet coherence, and the newly proposed time-varying parameter vector autoregression-based frequency connectedness approach (Chatziantoniou et al. in Integration and risk transmission in the market for crude oil a time-varying parameter frequency connectedness approach. Technical report, University of Pretoria, Department of Economics, 2021) using intraday high-frequency data. The empirical results demonstrate that the comovement of realized volatility between BTC and other cryptocurrencies is stronger than that of the realized skewness, realized kurtosis, and signed jump variation. The comovements among cryptocurrencies are both time-dependent and frequency-dependent. Besides the volatility spillovers, the risk spillovers of high-order moments and jumps are also significant, although their magnitudes vary with moments, making them moment-dependent as well and are lower than volatility connectedness. Frequency connectedness demonstrates that the risk connectedness is mainly transmitted in the short term (1–7 days). Furthermore, the total dynamic connectedness of all realized moments is time-varying and has been significantly affected by the outbreak of the COVID-19 pandemic. Several practical implications are drawn for crypto investors, portfolio managers, regulators, and policymakers in optimizing their investment and risk management tactics.

## Introduction

Cryptocurrencies, which are completely decentralized, have experienced rapid development recently and attracted widespread attention from investors, regulators, speculators, media, and academics (Mensi et al. 2021). Cryptocurrency differs from traditional fiat currencies in that it builds a new distributed payment system based on cryptographical protocols (Yi et al. 2018). In April 2021, the market capitalization of Bitcoin, the most notable cryptocurrency, reached an all-time high and had grown by more than US$1,000 billion compared to the summer months.^1^ According to the latest data,^2^ the capitalization of Bitcoin is US$573.32 billion. Bitcoin is driving the global payment system to a new level (Mamun et al. 2021). The second-largest cryptocurrency is Ethereum and its capitalization is US$247.26 billion.^3^ The other newly proposed cryptocurrencies, such as Ripple, Litecoin, Stellar, and Dash, are also remarkable and gradually reducing Bitcoin’s dominant market-value share, suggesting that speculators and investors have more alternative cryptocurrencies (Ji et al. 2019).

For now, cryptocurrencies have been considered as a significant part of the global financial market (Gajardo et al. 2018) and a new asset class (Mamun et al. 2021). Besides, many fund managers have also considered currencies a desirable asset class and pinned their hopes on them to gain high returns despite their extreme volatility (Ji et al. 2019). Furthermore, cryptocurrencies have been regarded as a ‘safe haven’ due to their low correlations with traditional assets and favorable performance during turbulent times (Kumar et al. 2022; Aharon et al. 2021). Cryptocurrencies have shown tremendous potential, and their trading volumes are skyrocketing (Hasan et al. 2022). Recent research on the comprehensive economic implications of cryptocurrency focuses on price discovery (Dimpfl and Peter 2021; Huang et al. 2022), market efficiency (Kakinaka and Umeno 2022; Montasser et al. 2022), volatility forecasting (Ma et al. 2020; Catania and Grassi 2021), tail risk (Borri 2019; Xu et al. 2021), bubble contagion (Chowdhury et al. 2022; Tiwari et al. 2020), herding behavior (Ballis and Drakos 2020; King and Koutmos 2021; Mandaci and Cagli 2022), and the speculative nature (Tan et al. 2020; Grobys and Junttila 2021) of cryptocurrencies.

Recently, the COVID-19 pandemic declared by the World Health Organization (WHO) on March 11, 2020 has been recognized as a global health crisis and exerted profound impacts on the global economy and financial markets. As asserted by Mnif et al. (2020), fear and anxiety caused by the COVID-19 pandemic have caused behavioral biases like herding behavior. After the outbreak of the COVID-19 pandemic, global financial markets tumbled, economic activity froze, and uncertainty spiked (Shahzad et al. 2021). It is worth noting that the prices of Bitcoin have surged dramatically during the COVID-19 pandemic and reached $24,000 (the highest since its introduction) in December 2020 (Ftiti et al. 2021). To boost the global economy under the COVID-19 pandemic, the quantitative easing (QE) monetary policy has been commonly implemented by worldwide central banks thus leading to a widespread fear that fiat currencies will most likely lose value (Shahzad et al. 2021). Bitcoin has been regarded as a ‘safe haven’ during the COVID-19 pandemic (Kumar et al. 2022; Aharon et al. 2021).

Against this backdrop, investors have become more interested in Bitcoin, so the demand for it continues to rise. A substantial body of literature has confirmed the impacts of the COVID-19 pandemic on contagion effects, interdependence, comovements, risk spillovers,^4^ and portfolio diversifications in global traditional financial markets (see Liu et al. 2022; Gharib et al. 2021; Boubaker et al. 2021; Zhang et al. 2022a; Narayan et al. 2022; Rehman et al. 2021; Abuzayed et al. 2021; Ali et al. 2021). As asserted by Lahmiri and Bekiros (2020), cryptocurrencies have been relatively more volatile than international stock markets during the COVID-19 pandemic. Owing to the lack of legal regulations and quality information, the cryptocurrency markets have failed to mature and deepen, which caused them to fluctuate excessively (Wątorek et al. 2021). Cryptocurrency prices have been subject to speculative frenzies due to the lack of appropriate legal and regulatory frameworks (Ahmed and Mafrachi 2021). In addition, the herding behavior^5^ in the cryptocurrency markets has greatly intensified during the COVID-19 outbreak, thus further exacerbating market volatility (Mandaci and Cagli 2022) and also exerting a great impact on the cryptocurrency market efficiency (Mnif et al. 2020). The impact of coronavirus media coverage on herding in the cryptocurrency market is significant, which explains the presence of herding during the COVID-19 pandemic crisis. The behavior of crypto investors is affected by news about the COVID-19 pandemic due to panic and fear. Therefore, investors neglect their own knowledge and imitate the investment strategies of other investors (Youssef and Waked 2022). This also leads to the increasing interdependence and spillovers among cryptocurrencies during the COVID-19 pandemic. In this context, several studies have also confirmed that the COVID-19 pandemic has exerted significant impacts on the interactions among cryptocurrencies (e.g. Yousaf and Ali 2020; Polat and Günay 2021; Naeem et al. 2021; Demiralay and Golitsis 2021; Raza et al. 2022; Kumar et al. 2022; Özdemir 2022; Ahmed and Sleem 2022). More importantly, asymmetric characteristics, tail risk, extreme volatility, and pricing bubbles in the cryptocurrency market have all been identified during the COVID-19 pandemic (see Nguyen et al. 2020; Xu et al. 2021; González et al. 2021; Apergis 2022; Iqbal et al. 2021; Montasser et al. 2022; Ahn 2022; Shahzad et al. 2022). Indeed, these typical features are all related to the higher-order moment risks, highlighting the necessity of analyzing the higher-order moment comovement and risk connectedness.

Existing research has thoroughly investigated the dynamic linkages, comovements, and risk connectedness among major cryptocurrencies. However, most studies are limited to the first and second moments of the return distribution, even though most cryptocurrencies exhibit extreme volatility and non-normally disturbed returns (Bouri et al. 2021a, b). In the cryptocurrency market, little attention has been paid to higher-order moment (i.e. skewness and kurtosis) comovements and risk connectedness. As asserted by Rubinstein (1973), a non-quadratic utility function and non-normal distribution of returns require investors to take into account not only the mean and variance but also higher-order moments (e.g., skewness and kurtosis). Thus, it is significant to explore the higher-order moment interlinkages among cryptocurrencies. Among them, Hasan et al. (2021) conducted the most relevant study for this paper, which investigated the realized higher moment (realized skewness and realized kurtosis) connectedness in the cryptocurrency market. However, they did not consider the asymmetric feature of volatility connectedness and the jump and fifth- and sixth-order moment (realized hyper-skewness and kurtosis) connectedness among cryptocurrencies. Another limitation is that the dynamic connectedness analysis based on the rolling-window method is vulnerable to the artificial setting of the rolling-window size, and the empirical results in the first rolling sample interval will be lost.^6^ Furthermore, Hasan et al. (2021) only quantified the higher-order moment risk connectedness in the time domain. They have not provided further evidence of risk connectedness in the frequency domain, preventing them from providing a more targeted reference for crypto investors and portfolio managers with different investment horizons. More importantly, higher-order moment comovements were not considered in this study.

Given the aforementioned shortcomings, this study investigates the higher-order moment comovements and risk connectedness among major cryptocurrencies from the time- and frequency-domain perspectives. Using the intraday 5-min high-frequency data, we first construct the realized moment measures. We explore the time–frequency comovements of realized volatility (RV), realized skewness (RS), realized kurtosis (RK), and signed jump variation (SJV) based on wavelet coherence. Regarding the quantification of connectedness, we employ the newly proposed time-varying parameter vector autoregression (TVP-VAR) based frequency connectedness approach by Chatziantoniou et al. (2021). We then quantify not only the RV, RS, and RK spillovers but also the realized semi-variance (RSV-P and RSV-N), SJV, and realized hyper-skewness and kurtosis (RHS and RHK) spillovers among major cryptocurrencies in both the time- and frequency-domain.

We have attempted to answer the following significant questions. How do cryptocurrencies co-move together at different frequency bands? What are the differences between the comovements at the second moment (RV) and higher-order moments (RS and RK)? Do differences exist in RV, RS, RK, RSV, SJV, RHS, and RHK spillovers? Are there significant asymmetric features of volatility spillover effects in the cryptocurrency market? Are the realized moment-based spillovers heterogeneous at different frequency bands? Which cryptocurrency is the leading net transmitter or receiver of the risk connectedness? Are the dynamic risk spillovers significantly intensified after the COVID-19 outbreak? What is the dynamic evolutionary trend of moment-based spillovers?

The motivations for analyzing the higher-order moment comovements and risk connectedness among major cryptocurrencies in the time and frequency domains can be elaborated as follows. First, cryptocurrency markets are usually filled with individual investors and speculators, whose trading activities are often featured in herding behavior (Vidal-Tomás et al. 2019; Silva et al. 2019; Hasan et al. 2022). Besides, the distributions of the cryptocurrency returns are non-normal (Baek and Elbeck 2015). The cryptocurrency markets also exhibit several distinguishing characteristics, including extreme tail risk and asymmetry (Feng et al. 2018; Borri 2019; Xu et al. 2021), speculative nature (Tan et al. 2020; Grobys and Junttila 2021), jumps and co-jumps (Bouri et al. 2021a; Walid et al. 2021) and pricing bubbles (Corbet et al. 2018; Chowdhury et al. 2022; Montasser et al. 2022). These characteristics are all related to higher-order moments of the return distributions, such as asymmetry and kurtosis, which give additional information on investors’ preferences for the risk-return trade-off (Silva et al. 2019). According to Amaya et al. (2015), higher-order moments, including skewness and kurtosis, are critical for investment decisions and risk management. The third moment (skewness) represents the likelihood of market crashes (i.e. asymmetry), whereas the fourth moment (kurtosis) represents the likelihood of extreme events (i.e. leptokurtosis and fat tails). Del Brio et al. (2017) asserted that examining interdependence through higher-order moments will provide a better understanding of the interlinkages among financial markets for investors and policymakers. The importance of high-order moments has been widely confirmed in financial studies, such as portfolio optimization (Zhao et al. 2021; Gülten and Ruszczyński 2015; Liu et al. 2020), return prediction (Jia et al. 2021), and asset pricing (Harvey and Siddique 2000; Nguyen and Puri 2009). Indeed, many studies have examined the higher-order moment comovement and risk spillovers among various financial markets, such as between developed and emerging stock markets (Del Brio et al. 2017), commodity and global financial markets (Gomez-Gonzalez et al. 2022), gold and oil markets (Bonato et al. 2020), carbon and energy markets (Dai et al. 2021), stock and commodity markets (Bouri et al. 2021a; Ahmed 2022), crude oil, gold and Bitcoin (Gkillas et al. 2022) and oil and commodity markets (Cui et al. 2022). Second, financial markets are typically made up of a diverse range of market participants with varying investment horizons. Market participants with short-term investment horizons (ranging from minutes to several days), such as speculators and day traders, are particularly concerned with short-term movements. In contrast, market participants with long-term investment horizons (ranging from several months to several years), including institutional investors and portfolio managers, are more susceptible to fundamental factors and long-term shocks (Wang and Wang 2019). Several studies have investigated the volatility connectedness among cryptocurrencies in the frequency domain (e.g. Mensi et al. 2021; Kumar et al. 2022; Mo et al. 2022). However, no studies to date have investigated the higher-order moment connectedness of major cryptocurrencies from a frequency-domain perspective.

The innovations and contributions of this paper to the related literature can be summarised as follows. First, to the best of our knowledge, this paper is the first to comprehensively investigate the higher-order moment comovements and risk connectedness among major cryptocurrencies from both the time- and frequency-domain perspectives. We shed new light on analyzing the comovements and risk connectedness in the cryptocurrency market. Compared to Hasan et al. (2021), we have examined both the higher-order moment connectedness and the time–frequency comovements among major cryptocurrencies. We further quantified the RSV, SJV, RHS, and RHK connectedness, besides the RV, RS, and RK connectedness. The asymmetric feature of the volatility spillovers has also been depicted. More importantly, we conducted the risk connectedness analysis not only in the time domain but also in the frequency domain (i.e. at different frequency bands). By considering the various investment horizons of market participants in comovement and spillover analysis, we can offer more targeted references for investors, regulators, and policymakers in portfolio optimization and systemic risk management.

Second, we develop a higher-order comovement and risk connectedness analysis framework. Based on the intraday 5-min high-frequency data, we first calculate daily realized moment measures. Then, we combine the realized moment measures with the wavelet coherence to portray the higher-order moment time–frequency comovements among cryptocurrencies. According to the directions of the phase arrows, we further identify the lead-lag relationships between Bitcoin and other cryptocurrencies. We apply the newly proposed TVP-VAR-based frequency connectedness approach of Chatziantoniou et al. (2021). We do not need to set the rolling-window size factitiously, and no observations are lost during the first rolling-window sample. We further constructed the directional net-pairwise connectedness network to identify the leading transmitter and recipient. By portraying the dynamics of the connectedness, we can identify the great impacts of a major crisis, such as the COVID-19 pandemic, on the dynamic higher-order moment risk spillovers among cryptocurrencies.

Finally, the empirical results of the time–frequency higher-order comovement and risk connectedness analysis can provide more practical references for cryptocurrency investors, speculators, and market regulators in their decision-making processes. Given that skewness measures asymmetry and crash risk, whereas kurtosis is associated with leptokurtosis and fat tails, the skewness comovement and connectedness results reveal how cryptocurrencies interact through asymmetry or crash risk. Furthermore, the kurtosis comovement and connectedness results show how fat-tail (extreme) risks spread among cryptocurrencies (Hasan et al. 2021). We also present a new empirical analysis framework for cryptocurrency comovement and risk spillovers. That is, we combine the realized higher-order moment measures with the wavelet coherence method to depict the higher-order comovements. Further, we combine the moment measures with the TVP-VAR Frequency connectedness approach to quantify the time–frequency higher-order moment risk connectedness among cryptocurrencies.

The remainder of this paper is organized as follows. “Literature review” section briefly reviews the existing related literature. “Econometric methods” section introduces the econometric methods. “Data and preliminary analysis” section provides the data and preliminary analysis. “Time–frequency comovement analysis” section presents the comovement results. “Realized moment connectedness results” section shows the connectedness results. “Discussion” section is the discussion part. Finally, “Conclusions and implications” section outlines the conclusions and implications.

## Literature review

In this section, we briefly review the state-of-art studies that are highly correlated to this paper. Indeed, before the outbreak of the COVID-19 pandemic, many researchers explored the return and volatility spillovers among cryptocurrencies. For example, using the LASSO-VAR-based connectedness approach, Yi et al. (2018) investigated the volatility connectedness among cryptocurrencies. They concluded that cryptocurrencies with high market capitalization transmit larger volatility spillovers to others, and Bitcoin contributes significant volatility spillovers to other cryptocurrencies, but it does not dominate the entire market. However, this finding is opposite to Koutmos (2018) that Bitcoin is the dominant transmitter of return and volatility spillovers among selected cryptocurrencies. Another representative study is Ji et al. (2019) which examined the dynamic return and volatility connectedness in cryptocurrency markets using the DY connectedness framework. The return connectedness network is centered on Litecoin and Bitcoin, with Bitcoin serving primarily as the largest net transmitter. This finding concurs with the empirical results of Koutmos (2018). Furthermore, Bouri et al. (2021b) examined the volatility connectedness among cryptocurrencies using the DCC-GARCH-based connectedness approach and concluded that investor sentiment is highly correlated with volatility connectedness. Other similar studies can be found in Kumar and Anandarao (2019), Katsiampa (2019), and Ji et al. (2021).

As the COVID-19 pandemic rages worldwide, a vast majority of studies have examined the return and volatility spillovers among cryptocurrencies considering the impacts of the COVID-19 pandemic. Among them, Yousaf and Ali (2020) applied the VAR-AGARCH model to analyze the return and volatility spillovers among Bitcoin, Ethereum, and Litecoin. They found that the spillovers show differences before and during the COVID-19 pandemic and the hedging effectiveness is higher during the COVID-19 period. Similarly, Özdemir (2022) examined the volatility spillover during the COVID‑19 pandemic based on DCC-GARCH and wavelet analysis. They found that the COVID-19 pandemic enhances the spillovers and causes more integrated cryptocurrency markets. This result is consistent with Raza et al. (2022) that the COVID-19 pandemic significantly caused higher connectedness among cryptocurrencies based on non-parametric causality-in-quantiles methods. Naeem et al. (2021) found that Bitcoin, Litecoin, and Ripple are the dominant transmitters of return spillovers; moreover, the post-COVID period shows tangled clusters across the cryptocurrencies. Considering the different volatility regimes, Shahzad et al. (2021) found different patterns of spillovers in high- and low-volatility regimes. The spillovers were relatively higher in the high volatility regime during the COVID-19 pandemic.

However, those studies only measure the spillovers in the time domain, ignoring the frequency dynamics. To fill this gap, Polat and Günay (2021) utilized the DY connectedness (Diebold and Yilmaz 2012, 2014) and the BK frequency connectedness (Baruník and Křehlík 2018) approaches to investigate the dynamic volatility connectedness among cryptocurrencies during the COVID-19 pandemic from time–frequency domains and found that the volatility spillovers intensified greatly after the outbreak of the COVID-19 pandemic and the connectedness varied with frequency bands. These results are also confirmed in the recent work of Kumar et al. (2022). Also, Mensi et al. (2021) concluded that Bitcoin, Ethereum, and Litecoin are the primary net transmitters of volatility spillovers, with short-term risk spillovers being stronger than medium- and long-term risk spillovers. Furthermore, using the BK frequency connectedness approach, Fousekis and Tzaferi (2021) investigated the frequency-domain connectedness between returns and volume in cryptocurrency markets. Most recently, Hasan et al. (2022) used the BK method to analyze liquidity connectedness in the cryptocurrency market and found that liquidity connectedness is higher in the short run than in the medium and long run. Furthermore, BTC, LTC, and XRP are the leading contributors to liquidity spillovers. Similar time–frequency spillover analysis in cryptocurrency markets can be found in Qiao et al. (2020) and Mensi et al. (2019a).

Due to the safe haven property, cryptocurrencies have been regarded as an alternative asset and obtain great popularity among investors. Hence, many scholars have investigated the risk relationships between Bitcoin and other financial assets during the COVID-19 pandemic. For instance, González et al. (2021) investigated the asymmetric interdependencies between large cryptocurrencies and gold using the nonlinear autoregressive distributed lag (NARDL) model. They found that during the COVID-19 pandemic, most cryptocurrencies respond more to negative changes and exhibit greater persistence with gold returns. Elsayed et al. (2022) also claimed that global shocks, such as the COVID-19 pandemic, have altered the comovements of cryptocurrency and other traditional financial assets significantly. Moreover, besides the time-domain connectedness, many researchers also explored the spillovers among cryptocurrencies in the frequency domain. Among them, Maghyereh and Abdoh (2022) examined the effects of the COVID-19 on volatility spillovers between Bitcoin and traditional assets (gold, oil, foreign exchange, stock, and bond) and concluded that volatility dynamics are weak or negative before the COVID-19 but become positive afterward. Most of these financial assets’ ability to hedge against Bitcoin shocks is desirable in the short and long term. Zhang et al. (2022b) examined the information spillovers from COVID-19-related news to the Bitcoin, crude oil, and gold markets; they found that the return and volatility spillovers from epidemic-related news are stronger in the short term (i.e., less than 1 week). Mo et al. (2022) investigated the time–frequency connectedness between cryptocurrencies and commodity markets and discovered that cryptocurrencies act as the primary transmitters of risk spillovers to the system both in the short and long run, and cryptocurrencies exhibit better hedging performance following the COVID-19 pandemic.

Intraday high-frequency trading data in the cryptocurrency market contains richer information, thus attracting extensive attention from investors and scholars. For instance, Mensi et al. (2019b) analyzed the high-frequency asymmetric volatility connectedness between Bitcoin and major precious metals markets. They concluded that the spillovers are sensitive to negative shocks and political events and confirmed the significant asymmetric feature of volatility connectedness. Sensoy et al. (2021) explored the high-frequency return and volatility spillovers among cryptocurrencies and found that the return and volatility have different spillover patterns among cryptocurrencies, and BTC, LTC, and ETH are the most connected cryptocurrencies. Ji et al. (2021) explored the realized volatility connectedness among Bitcoin exchange markets based on the intraday hourly data and concluded that Coinbase is the largest net transmitter of volatility spillovers, whereas EXMO is the largest net recipient. Most recently, Katsiampa et al. (2022) explored the high-frequency connectedness between Bitcoin and other top-traded crypto assets during the COVID-19 crisis and found that there exists a positive contagion effect in cryptocurrency markets.

The comovements, interdependence, and dynamic linkages among cryptocurrencies are of great significance for cryptocurrency portfolio optimization and risk management. Given this, Qiao et al. (2020) depicted the time–frequency comovement of cryptocurrency return and volatility. The results demonstrate positive correlations between Bitcoin and other cryptocurrencies and that Bitcoin has hedging effects on other cryptocurrencies. Qureshi et al. (2020) also concluded that the coherence among cryptocurrencies tends to fluctuate at higher frequencies and is significantly stable at lower frequencies. Also, several researchers have noted the significant tail dependence in the cryptocurrency market. Using a LASSO quantile regression approach, Nguyen et al. (2020) concluded that the right tail dependence among cryptocurrencies is significantly stronger than the left tail. More recently, Shahzad et al. (2022) investigated the return interdependence among cryptocurrencies under both normal and extreme market conditions. They found that return interdependence is greater in the right tail, and Bitcoin is not the leading risk net transmitter or net receiver. In contrast, Ahn (2022) found that the downward tail correlations among cryptocurrencies and equities are much stronger than the upward tail correlations. Similar studies can be also found in Tiwari et al. (2020), González et al. (2021), Chowdhury et al. (2022) and Cao and Ling (2022). Furthermore, the dynamic linkages among cryptocurrencies have also attracted the broad attention of many scholars. Among them, Yousaf and Ali (2020) studied the interlinkages among major cryptocurrencies using intraday high-frequency data. They found that the dynamic linkages, hedging cost, and hedging effectiveness of cryptocurrencies are all relatively higher during the COVID-19 pandemic. Bouri et al. (2021c) applied the dynamic equicorrelation (DECO)-GARCH model to examine the market integration among leading cryptocurrencies and found that there exists a high market integration in the cryptocurrency market and trading volume and uncertainties are the main determining factors. Similarly, Demiralay and Golitsis (2021) also employed this approach and concluded that the interlinkages among cryptocurrencies greatly increased after the outbreak of the COVID-19 pandemic. Another research highly related to this study is conducted by Ahmed and Sleem (2022) who explored the moment interdependence of equity, oil, and gold markets during the COVID-19 pandemic. The difference between this study and our paper lies in that besides the RV, RS, and RK comovements, we have also investigated the SJV (jump) comovements. More importantly, we have further quantified the RV, RS, RK, SJV, RSV, RHS, and RHK connectedness among leading cryptocurrencies.

## Econometric methods

### Realized moment measures

We construct the realized moment measures based on the intraday logarithmic returns. For each cryptocurrency high-frequency price series, we calculate the intraday returns on the day $t$ for the *i*th intraday price as the natural logarithmic difference between two continuous price observations within a trading day.

1 $$r_{t,i} = \ln (p_{t,i} ) - \ln (p_{t,i - 1} )$$

where $r_{t,i}$ denotes the intraday returns of cryptocurrencies and $p_{t,i}$ represents the *i*-th intraday price.

The realized volatility (RV) can be expressed by (Andersen and Bollerslev 1998)

2 $$RV_{t} = \sum\limits_{i = 1}^{T} {r_{t,i}^{2} }$$

where $i = (1,2,...,T)$ and $T$ denotes the number of the intraday logarithmic returns.

We then build the realized semi-variance (RSV). Positive semi-variance (RSV-P) and negative semi-variance (RSV-N) are presented as follows (Barndorff-Nielsen et al. 2008; Patton and Sheppard 2015):

3 $$RSVN_{t} = \sum\limits_{i = 1}^{T} {r_{t,i}^{2} } I(r_{t,i} < 0)$$

4 $$RSVP_{t} = \sum\limits_{i = 1}^{T} {r_{t,i}^{2} } I(r_{t,i} > 0)$$

where $I( \bullet )$ is an indicator function.

Per Patton and Sheppard (2015), we define daily signed jump variation (SJV) as:

5 $$SJV_{t} = RSVP_{t} - RSVN_{t}$$

Following Amaya et al. (2015), we construct the realized skewness (RS) and realized kurtosis (RK):

6 $$RS_{t} = {{\sqrt T \sum\limits_{i = 1}^{T} {r_{t,i}^{3} } } \mathord{\left/ {\vphantom {{\sqrt T \sum\limits_{i = 1}^{T} {r_{t,i}^{3} } } {RV_{t}^{{{3 \mathord{\left/ {\vphantom {3 2}} \right. \kern-\nulldelimiterspace} 2}}} }}} \right. \kern-\nulldelimiterspace} {RV_{t}^{{{3 \mathord{\left/ {\vphantom {3 2}} \right. \kern-\nulldelimiterspace} 2}}} }}$$

7 $$RK_{t} = {{T\sum\limits_{i = 1}^{T} {r_{t,i}^{4} } } \mathord{\left/ {\vphantom {{T\sum\limits_{i = 1}^{T} {r_{t,i}^{4} } } {RV_{t}^{2} }}} \right. \kern-\nulldelimiterspace} {RV_{t}^{2} }}$$

Per Khademalomoom et al. (2019), Kinateder and Papavassiliou (2019), and Ahmed and Mafrachi (2021), we further define the realized hyper-skewness (RHS) and kurtosis (RHK)^7^:

8 $$RHS_{t} = {{T^{{{3 \mathord{\left/ {\vphantom {3 2}} \right. \kern-\nulldelimiterspace} 2}}} \sum\limits_{i = 1}^{T} {r_{t,i}^{5} } } \mathord{\left/ {\vphantom {{T^{{{3 \mathord{\left/ {\vphantom {3 2}} \right. \kern-\nulldelimiterspace} 2}}} \sum\limits_{i = 1}^{T} {r_{t,i}^{5} } } {RV_{t}^{{{5 \mathord{\left/ {\vphantom {5 2}} \right. \kern-\nulldelimiterspace} 2}}} }}} \right. \kern-\nulldelimiterspace} {RV_{t}^{{{5 \mathord{\left/ {\vphantom {5 2}} \right. \kern-\nulldelimiterspace} 2}}} }}$$

9 $$RHK_{t} = {{T^{2} \sum\limits_{i = 1}^{T} {r_{t,i}^{6} } } \mathord{\left/ {\vphantom {{T^{2} \sum\limits_{i = 1}^{T} {r_{t,i}^{6} } } {RV_{t}^{3} }}} \right. \kern-\nulldelimiterspace} {RV_{t}^{3} }}$$

### Wavelet coherence

We employ the continuous wavelet method to depict the time–frequency comovements of cryptocurrencies. The continuous wavelet transform (CWT) separates the original series into time and frequency components, which allows us to offer information on the time and over various frequency scales. CWT does not require a requirement for portraying wavelets (timescales) (Qureshi et al. 2020). CWT measures the similarity between two functions (time series and analyzing functions) based on the inner product (Gencay et al. 2002; Singh et al. 2019). The CWT method has been extensively applied in recent studies to explore the comovement effects among cryptocurrencies (e.g., Mensi 2019a; Qiao et al. 2020; Qureshi et al. 2020; Celeste et al. 2020).

Accordingly, the continuous wavelet transform of a time series can be defined as (Singh et al. 2019):

10 $$W_{x} \left( {\tau ,s} \right) = \frac{1}{\sqrt s }\int_{ - \infty }^{\infty } {x(t)\frac{1}{\sqrt s }} \psi \left( {\frac{t - \tau }{s}} \right)dt$$

where ${1 \mathord{\left/ {\vphantom {1 {\sqrt s }}} \right. \kern-\nulldelimiterspace} {\sqrt s }}$ denotes a normalization factor, $s$ represents a scaling factor, $\tau$ denotes a translation parameter.

The Morlet wavelet^8^ is applied in this paper to obtain the amplitude and phase angle of the original signal.

11 $$\psi \left( t \right) = \pi^{{ - \frac{1}{4}}} e^{{i\omega_{0} t}} e^{{ - \frac{{t^{2} }}{2}}}$$

where $1/(\pi^{{{1 \mathord{\left/ {\vphantom {1 4}} \right. \kern-\nulldelimiterspace} 4}}} )$ ensures unity energy of the wavelet, $\omega_{0}$ represents the dimensionless frequency.

Using the continuous wavelet transform, we can obtain squared wavelet coherence.

12 $$R^{2} \left( {\tau ,s} \right) = \frac{{\left| {S\left( {s^{ - 1} W_{xy} \left( {\tau ,s} \right)} \right)} \right|^{2} }}{{S\left( {s^{ - 1} \left| {W_{x} \left( {\tau ,s} \right)} \right|^{2} } \right)S\left( {s^{ - 1} \left| {W_{y} \left( {\tau ,s} \right)} \right|^{2} } \right)}}$$

where $S( \bullet )$ signifies the time and scale smoothing operator.

In addition, we compute the wavelet phase difference to identify the comovement directions (i.e. the lead-lag relationship) among cryptocurrency markets. The wavelet phase difference can be expressed as follows:

13 $$\phi_{xy} (\mu ,s) = \tan^{ - 1} \left( {\frac{{\Im \left\{ {S\left( {s^{ - 1} W_{xy} \left( {\mu ,s} \right)} \right)} \right\}}}{{\Re \left\{ {S\left( {s^{ - 1} W_{xy} \left( {\mu ,s} \right)} \right)} \right\}}}} \right)$$

where $\Im$ represents an imaginary part operator and $\Re$ indicates a real part operator. The wavelet phase difference is presented by the directions of the arrows. A rightward (leftward) arrow demonstrates that the series $x\left( t \right)$ and $y\left( t \right)$ are in phase (out of phase); namely, they are positively (negatively) correlated, with negligible or no time lag (Ahmed 2022). Regarding the lead-lag relationships, right-up (left-down) arrows suggest that $x\left( t \right)$ positively (negatively) leads $y\left( t \right)$, whereas right-down (left-up) arrows indicate that $y\left( t \right)$ positively (negatively) leads *x*(*t*) (Ahmed 2022; Kartal et al. 2021).

### TVP-VAR-based frequency connectedness approach

We investigate the moment-based risk connectedness among major cryptocurrencies using the TVP-VAR-based frequency connectedness approach. Chatziantoniou et al. (2021) combined the Frequency Connectedness of Baruník and Křehlík (2018) and the TVP-VAR-based connectedness of Antonakakis et al (2020). We briefly introduce the TVP-VAR-based connectedness approach, which is based on the work of Chatziantoniou et al. (2021), Gabauer and Gupta (2018), and Antonakakis et al. (2020). The $p$-order lag time-varying parameter vector autoregression can be given by:

14 $$x_{t} = \Phi_{1t} x_{t - 1} + \Phi_{2t} x_{t - 2} + \ldots + \Phi_{pt} x_{t - p} + \xi_{t} \quad \xi_{t} \sim N\left( {0,\Sigma_{t} } \right)$$

where $x_{t}$ and $\xi_{t}$ are $N \times 1$ dimensional vectors, $\Phi_{it}$ denotes the time-varying coefficients of the VAR model, $\sum_{t}$ represents the time-varying variance–covariance matrix.

The generalized forecast error variance decomposition (GFEVD) can be expressed as:

15 $$\theta_{ijt} (H) = {{\left\{ {\left( {\Sigma_{t} } \right)_{jj}^{ - 1} \sum\limits_{h = 0}^{H} {\left( {\left( {\Psi_{h} \Sigma_{t} } \right)_{ijt} } \right)^{2} } } \right\}} \mathord{\left/ {\vphantom {{\left\{ {\left( {\Sigma_{t} } \right)_{jj}^{ - 1} \sum\limits_{h = 0}^{H} {\left( {\left( {\Psi_{h} \Sigma_{t} } \right)_{ijt} } \right)^{2} } } \right\}} {\left\{ {\sum\limits_{h = 0}^{H} {\left( {\Psi_{h} {{\varvec{\Sigma}}}_{t} \Psi_{h}^{\prime } } \right)_{ii} } } \right\}}}} \right. \kern-\nulldelimiterspace} {\left\{ {\sum\limits_{h = 0}^{H} {\left( {\Psi_{h} {{\varvec{\Sigma}}}_{t} \Psi_{h}^{\prime } } \right)_{ii} } } \right\}}}$$

16 $$\tilde{\theta }_{ijt} (H) = {{\left\{ {\theta_{ijt} (H)} \right\}} \mathord{\left/ {\vphantom {{\left\{ {\theta_{ijt} (H)} \right\}} {\left\{ {\sum\limits_{k = 1}^{N} {\theta_{ijt} } (H)} \right\}}}} \right. \kern-\nulldelimiterspace} {\left\{ {\sum\limits_{k = 1}^{N} {\theta_{ijt} } (H)} \right\}}}$$

where $\widetilde{\theta }_{ijt} \left( H \right)$ measures the contribution of the cryptocurrency $i$ to the variance of the forecast error of the cryptocurrency $j$ at the horizon $H$. The TVP-VAR-based connectedness measures are as follows.

17 $$\left\{ \begin{gathered} TO_{it} (H) = \sum\limits_{i = 1,i \ne j}^{N} {\tilde{\theta }_{jit} } (H);\;\;FROM_{it} (H) = \sum\limits_{j = 1,i \ne j}^{N} {\tilde{\theta }_{ijt} } (H); \hfill \\ NET_{it} \left( H \right) = TO_{it} \left( H \right) - FROM_{it} \left( H \right);\;\;NPDC_{ijt} \left( H \right) = \widetilde{\theta }_{ijt} \left( H \right) - \widetilde{\theta }_{jit} \left( H \right); \hfill \\ TCI_{t} \left( H \right) = N^{ - 1} \sum\nolimits_{i = 1}^{N} {TO_{it} \left( H \right)} = N^{ - 1} \sum\nolimits_{i = 1}^{N} {FROM_{it} \left( H \right)} \hfill \\ \end{gathered} \right.$$

where $TO_{it} \left( H \right)$ and $FROM_{it} \left( H \right)$ are directional total connectedness transmitted (received) to (from) other markets, $NET_{it} \left( H \right)$ denotes directional net connectedness, $NPDC_{ijt} \left( H \right)$ is directional net pairwise connectedness, and $TCI_{t} \left( H \right)$ represents the total connectedness index.

The spectral density of $x_{t}$ at the frequency $\omega$ can be defined as a Fourier Transformation of the TVP-VMA (Chatziantoniou et al. 2021):

18 $$S_{x} (\omega ) = \sum\limits_{h = - \infty }^{\infty } E \left( {x_{t} x_{t - h}^{\prime } } \right)e^{ - i\omega h} = \psi \left( {e^{ - i\omega h} } \right){{\varvec{\Sigma}}}_{t} \psi^{\prime } \left( {e^{ + i\omega h} } \right)$$

Then, the frequency GFEVD can be stated as:

19 $$\theta_{ijt} (\omega ) = {{\left\{ {\left( {\Sigma_{t} } \right)_{jj}^{ - 1} \left| {\sum\limits_{h = 0}^{\infty } {\left( {\Psi \left( {e^{ - i\omega h} } \right)\Sigma_{t} } \right)_{ijt} } } \right|^{2} } \right\}} \mathord{\left/ {\vphantom {{\left\{ {\left( {\Sigma_{t} } \right)_{jj}^{ - 1} \left| {\sum\limits_{h = 0}^{\infty } {\left( {\Psi \left( {e^{ - i\omega h} } \right)\Sigma_{t} } \right)_{ijt} } } \right|^{2} } \right\}} {\left\{ {\sum\limits_{h = 0}^{\infty } {\left( {\Psi \left( {e^{ - i\omega h} } \right)\Sigma_{t} \Psi \left( {e^{i\omega h} } \right)} \right)_{ii} } } \right\}}}} \right. \kern-\nulldelimiterspace} {\left\{ {\sum\limits_{h = 0}^{\infty } {\left( {\Psi \left( {e^{ - i\omega h} } \right)\Sigma_{t} \Psi \left( {e^{i\omega h} } \right)} \right)_{ii} } } \right\}}}$$

20 $$\tilde{\theta }_{ijt} (\omega ) = {{\left\{ {\theta_{ijt} (\omega )} \right\}} \mathord{\left/ {\vphantom {{\left\{ {\theta_{ijt} (\omega )} \right\}} {\left\{ {\sum\limits_{k = 1}^{N} {\theta_{ijt} } (\omega )} \right\}}}} \right. \kern-\nulldelimiterspace} {\left\{ {\sum\limits_{k = 1}^{N} {\theta_{ijt} } (\omega )} \right\}}}$$

where $\widetilde{\theta }_{ijt} \left( \omega \right)$ denotes the percentage of the spectrum of the market $i$ at a specific frequency $\omega$.

To quantify the connectedness at various frequency bands, we gather all frequencies into a specific range.

21 $$\tilde{\theta }_{ijt} (d) = \int_{a}^{b} {\tilde{\theta }_{ijt} (\omega )d\omega }$$

where $$d = (a,b),a,b \in ( - \pi ,\pi ),a < b$$.

Finally, the relationships between DY time-domain connectedness (Diebold and Yilmaz 2012, 2014) and BK frequency-domain connectedness (Baruník and Křehlík 2018) measures are presented as follows:

22 $$TO_{it} (H) = \sum\limits_{d} T O_{it} (d) = \sum\limits_{d} {\left( {\sum\limits_{i = 1,i \ne j}^{N} {\tilde{\theta }_{jit} } (d)} \right)}$$

23 $$FROM_{it} (H) = \sum\limits_{d} F ROM_{it} (d) = \sum\limits_{d} {\left( {\sum\limits_{i = 1,i \ne j}^{N} {\tilde{\theta }_{ijt} } (d)} \right)}$$

24 $$NET_{it} (H) = \sum\limits_{d} N ET_{it} (d) = \sum\limits_{d} {\left\{ {\left( {\sum\limits_{i = 1,i \ne j}^{N} {\tilde{\theta }_{jit} } (d)} \right) - \left( {\sum\limits_{i = 1,i \ne j}^{N} {\tilde{\theta }_{ijt} } (d)} \right)} \right\}}$$

25 $$NPDC_{ijt} (H) = \sum\limits_{d} N PDC_{ijt} (d) = \sum\limits_{d} {\left( {\tilde{\theta }_{ijt} (d) - \tilde{\theta }_{jit} (d)} \right)}$$

26 $$TCI_{t} (H) = \sum\limits_{d} T CI_{t} (d) = \sum\limits_{d} {\left( {N^{ - 1} \sum\limits_{i = 1}^{N} T O_{it} (d)} \right)} = \sum\limits_{d} {\left( {N^{ - 1} \sum\limits_{i = 1}^{N} F ROM_{it} (d)} \right)}$$

where $TO_{it} (d)$, $FROM_{it} (d)$, $NET_{it} \left( d \right)$, $NPDC_{it} (d)$ and $TCI_{it} (d)$ are frequency spillover measures.

## Data and preliminary analysis

### Data source

**Fig. 1:**
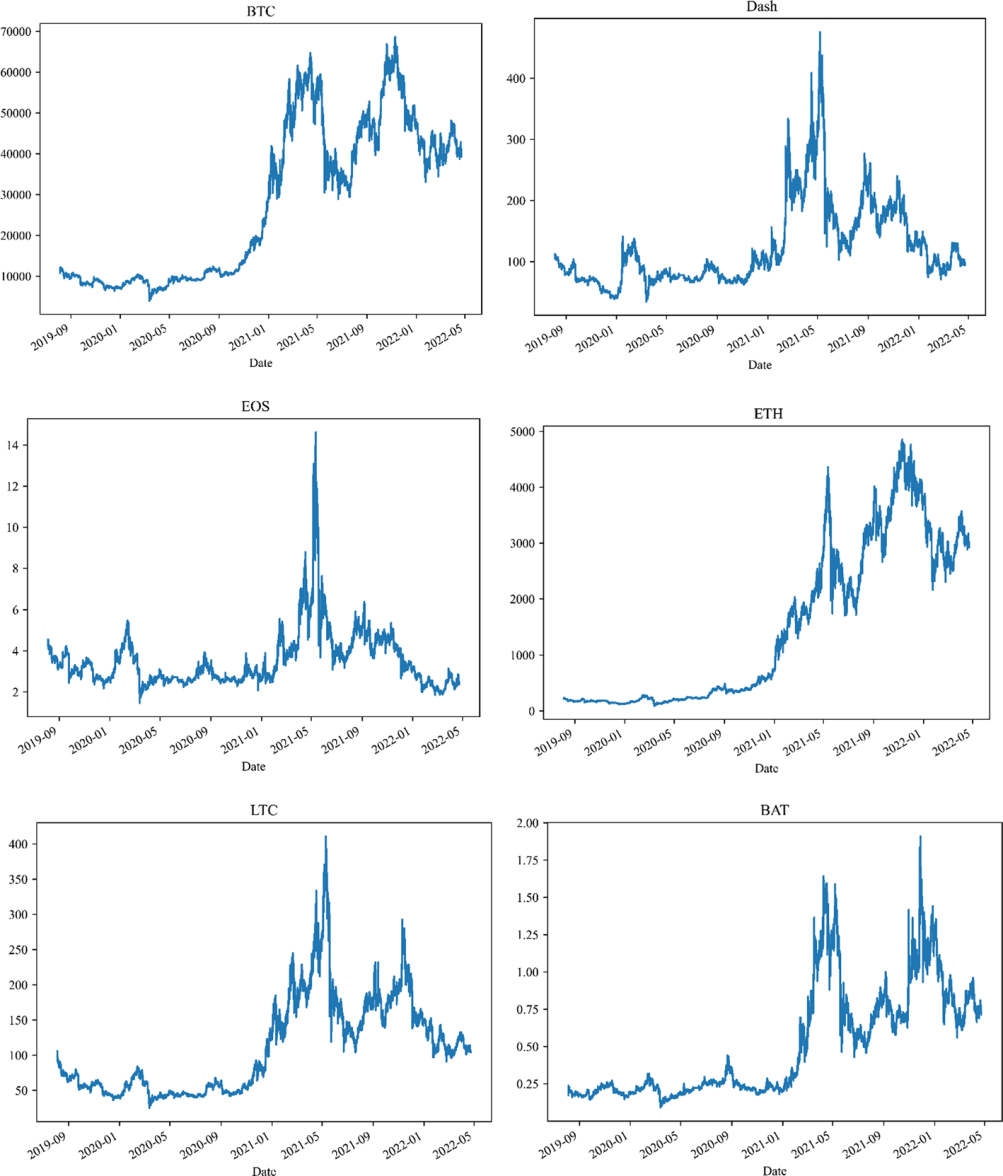
Intraday 5-min high-frequency closing prices for cryptocurrencies

This study explores the comovement and risk connectedness among major cryptocurrencies. Thus, we chose Bitcoin (BTC), Dash, EOS, Ethereum (ETH), Litecoin (LTC), and Basic Attention Token (BAT). These cryptocurrencies have recently piqued the interest of policymakers and investors, and they are widely used in academic research (e.g. Hasan et al. 2021, 2022; Shahzad et al. 2021 among others). Dukascopy (www.dukascopy.com) provided the intraday 5-min high-frequency data^9^ for cryptocurrencies. All chosen cryptocurrencies are priced in USD. The dataset spans the period from 5 August 2019 to 23 April 2022^10^ and includes major crises, such as the ongoing COVID-19 pandemic and the Russian-Ukrainian war. This dataset enables us to assess the effects of major crises on the time–frequency dependence and connectedness among cryptocurrencies. Figure 1 depicts the dynamics of cryptocurrency intraday high-frequency closing prices. We find that BTC, ETH, and BAT prices exhibit a similar evolution feature. The prices fluctuate at lower levels in 2019 and 2020, then rise rapidly and reach an all-time high in April 2021. The prices fall rapidly until rising again in July 2021 and peaked in November 2021, before falling again. Notably, Dash, EOS, and LTC prices also show similar variations. The prices oscillate at low levels until January 2021, then begin to rise rapidly, peaking in May 2021, before falling rapidly again.

### Descriptive statistics

Table 1 displays descriptive statistics for the daily moment measures, including RV, RS, RK, and SJV. In terms of RV, LTC has the highest mean value, followed by Dash. Meanwhile, Dash has the highest standard deviation, indicating that its RV is the most volatile. BTC has the smallest standard deviation. The mean RS values for ETH and LTC are positive, while the others are negative. The RS for BTC and ETH have more volatile characteristics. Furthermore, BTC has the highest RK, followed by ETH, implying that extreme events are more likely in the BTC and ETH markets. Similarly, the RK for BTC and ETH show the most volatility. The SJV mean values for all cryptocurrencies are roughly equal to zero and negative (except for Dash). The SJV for BAT exhibited the highest volatility. The kurtosis and Jarque–Bera test results indicate that all realized moments are non-normal. The ADF and PP unit root test results demonstrate that all daily realized moment measures are stationary, thus satisfying the condition of our modeling.

**Table 1:** Descriptive statistics of daily realized moments

	Mean	SD	Skewness	Kurtosis	Jarque–Bera	ADF	PP
*Panel A: Realized Volatility (RV)*							
BTC	0.001822	0.004654	14.98685	302.2478	3,501,079***	− 14.93552***	− 22.70821***
Dash	0.007937	0.078771	22.69180	540.9226	11,280,399***	− 14.31224***	− 17.97840***
EOS	0.004337	0.010198	12.53668	225.4151	1,939,178***	− 10.52139***	− 26.68899***
ETH	0.003769	0.006946	11.14418	174.1410	1,152,967***	− 7.664114***	− 24.26728***
LTC	0.008497	0.009734	8.060229	104.9441	412,339.2***	− 7.829643***	− 23.60590***
BAT	0.006143	0.011404	14.55549	308.8150	3,652,917***	− 23.40249***	− 24.61295***
*Panel B: Realized Skewness (RS)*							
BTC	− 0.016939	3.746830	0.104197	14.93655	5516.891***	− 31.78434***	− 31.76607***
Dash	− 0.147581	1.812458	− 0.275905	22.33551	14,483.37***	− 30.87494***	− 30.87634***
EOS	− 0.243857	2.387582	− 0.842229	17.01832	7716.528***	− 29.50464***	− 29.49814***
ETH	0.064291	3.491486	− 0.061328	20.55325	11,927.26***	− 32.72021***	− 32.67073***
LTC	0.050513	3.069305	0.589382	28.33280	24,894.88***	− 32.43155***	− 32.43120***
BAT	− 0.103550	1.407083	1.869004	38.58945	49,569.18***	− 32.26440***	− 32.05995***
*Panel C: Realized Kurtosis (RK)*							
BTC	25.60290	54.14920	3.905588	18.16178	11,260.03***	− 5.370928***	− 30.61559***
Dash	12.44279	20.85579	6.138808	55.13884	111,061.9***	− 4.171099***	− 28.30612***
EOS	16.31601	29.25903	4.993374	32.99441	38,685.10***	− 7.498770***	− 30.48970***
ETH	20.36547	54.71493	4.307199	20.66446	14,950.75***	− 5.156628***	− 30.84059***
LTC	19.64629	49.68773	4.809156	25.59066	23,335.32***	− 4.972393***	− 33.73456***
BAT	10.28841	18.64893	6.837938	65.02460	156,152.5***	− 9.137507***	− 32.41556***
*Panel D: Signed Jump Variation (SJV)*							
BTC	− 0.000031	0.000812	− 2.519562	50.94549	89,964.48***	− 33.58385***	− 33.60951***
Dash	0.000004	0.003145	3.839030	174.9927	1,147,332***	− 28.93107***	− 28.93923***
EOS	− 0.000324	0.003589	− 8.653027	140.7738	746,340.2***	− 30.13224***	− 30.13122***
ETH	− 0.000009	0.001453	− 1.550353	59.85337	125,489.3***	− 33.90443***	− 33.80057***
LTC	− 0.00009	0.002189	− 7.043786	119.5256	533,272.5***	− 30.42290***	− 30.48398***
BAT	− 0.000077	0.004941	− 2.546340	310.9413	3,671,633***	− 34.27580***	− 33.73035***

The numerical results presented in the table show the basic descriptive statistics of the daily realized moment measures for cryptocurrencies. The Jarque–Bera statistic is used to test the normality of the sample distribution. The unit root tests ADF (Augmented Dickey-Fuller) and PP (Phillips-Perron) are used to test the stationarity of the daily realized moment measures. *** denotes statistical significance at the 1% level

### Unconditional correlation analysis

**Fig. 2:**
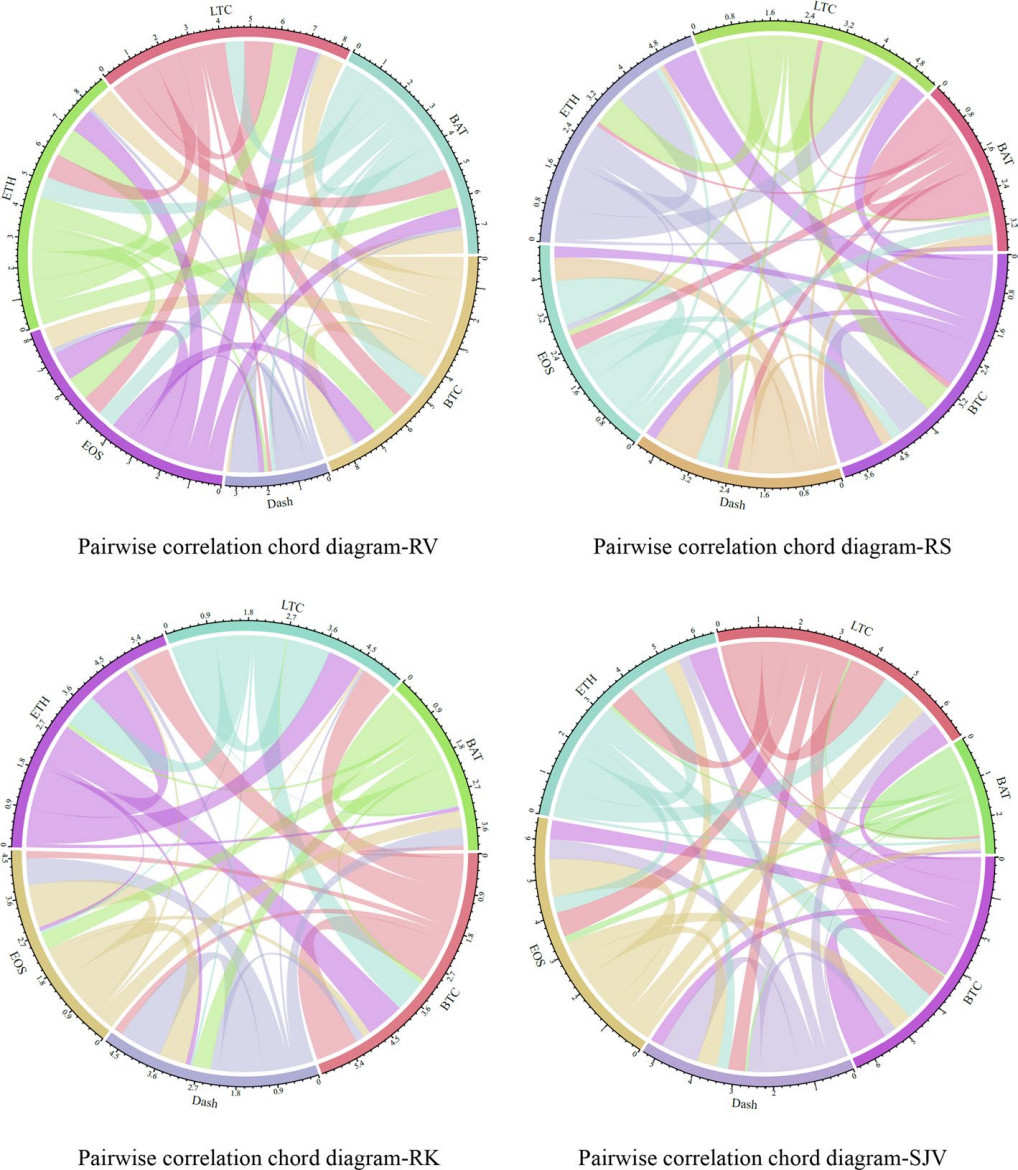
Unconditional correlation chord diagram

Table 2 presents the Pearson correlation matrix of different realized moments, while Fig. 2 shows the chord diagram of the pairwise correlation. Generally, the unconditional correlations of RV for cryptocurrencies are higher than those for other realized moments. This implies that the RV connectedness may be higher than other realized moment connectedness. Specifically, we find that BTC exhibits the highest correlation with ETH for all realized moments (RV-0.9019, RS-0.7735, RK-0.8859, and SJV-0.7555), followed by LTC. Besides, Dash is more correlated with EOS (0.6244) at the realized fourth moment (RK). However, the correlation between RV for Dash and EOS is relatively lower (0.187993), indicating a significant difference between the correlations of realized volatility and higher-order moments. At the realized second moment (RV), EOS also has a relatively higher correlation with ETH. Overall, BAT has lower correlations with other cryptocurrencies for RS, RK, and SJV. The unconditional correlation analysis provides a preliminary description of the relationship among cryptocurrencies. However, the Pearson correlation is both unconditional and static.

**Table 2:** Unconditional correlation matrix of daily realized moments

	BTC	Dash	EOS	ETH	LTC	BAT
*Panel A: Realized Volatility (RV)*						
BTC	1.000000	0.102604	0.749265	0.901921	0.827615	0.802753
Dash	0.102604	1.000000	0.187993	0.128894	0.122714	0.136312
EOS	0.749265	0.187993	1.000000	0.822094	0.699581	0.634966
ETH	0.901921	0.128894	0.822094	1.000000	0.840007	0.724390
LTC	0.827615	0.122714	0.699581	0.840007	1.000000	0.654917
BAT	0.802753	0.136312	0.634966	0.724390	0.654917	1.000000
*Panel B: Realized Skewness (RS)*						
BTC	1.000000	0.242808	0.252156	0.773499	0.638008	0.126200
Dash	0.242808	1.000000	0.514639	0.104678	0.103753	0.235356
EOS	0.252156	0.514639	1.000000	0.118706	0.129093	0.325813
ETH	0.773499	0.104678	0.118706	1.000000	0.692752	0.083513
LTC	0.638008	0.103753	0.129093	0.692752	1.000000	0.099897
BAT	0.126200	0.235356	0.325813	0.083513	0.099897	1.000000
*Panel C: Realized Kurtosis (RK)*						
BTC	1.000000	0.197618	0.169081	0.885853	0.747158	0.101645
Dash	0.197618	1.000000	0.624418	0.111708	0.058586	0.416170
EOS	0.169081	0.624418	1.000000	0.084971	0.059390	0.396050
ETH	0.885853	0.111708	0.084971	1.000000	0.795237	0.093410
LTC	0.747158	0.058586	0.059390	0.795237	1.000000	0.027974
BAT	0.101645	0.416170	0.396050	0.093410	0.027974	1.000000
*Panel D: Signed Jump Variation (SJV)*						
BTC	1.000000	0.329992	0.485401	0.755450	0.610130	− 0.057630
Dash	0.329992	1.000000	0.523501	0.304977	0.439522	0.064912
EOS	0.485401	0.523501	1.000000	0.423126	0.638753	0.189787
ETH	0.755450	0.304977	0.423126	1.000000	0.662366	− 0.085262
LTC	0.610130	0.439522	0.638753	0.662366	1.000000	0.062005
BAT	− 0.057630	0.064912	0.189787	− 0.085262	0.062005	1.000000

## Time–frequency comovement analysis

**Fig. 3:**
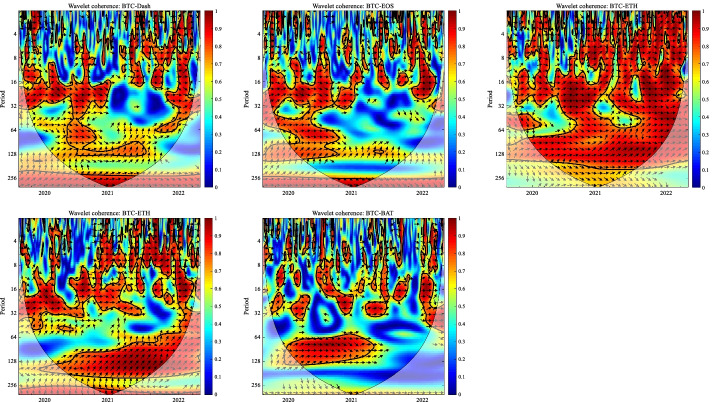
Wavelet coherence—realized volatility (RV)

**Fig. 4:**
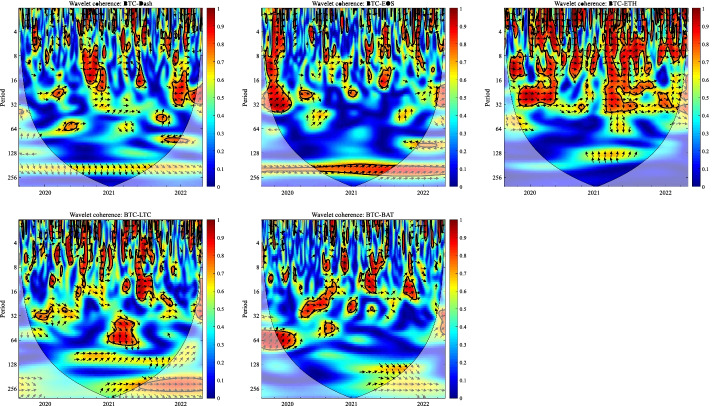
Wavelet coherence—realized skewness (RS)

**Fig. 5:**
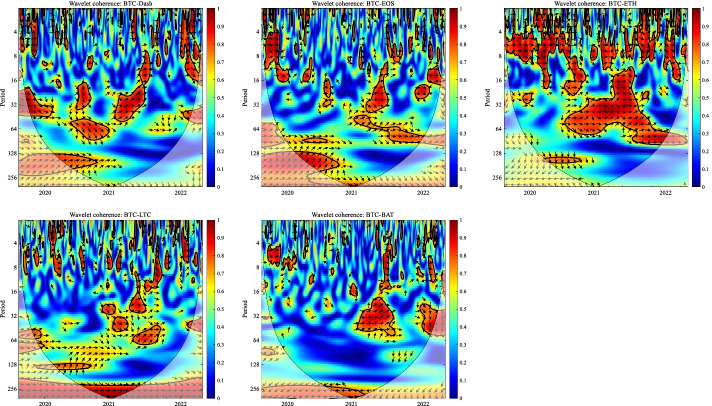
Wavelet coherence—realized kurtosis (RK)

**Fig. 6:**
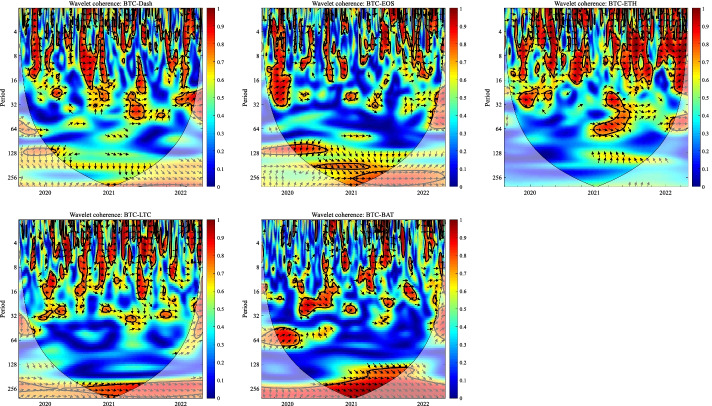
Wavelet coherence—signed jump variation (SJV)

In this section, we apply the wavelet coherence method to analyze the time–frequency dependence among major cryptocurrencies from the higher-order moment perspective. Using wavelet coherence, we can detect regions in time–frequency space where the examined time series move together, but not necessarily at high common power (Vacha and Barunik 2012). Figures 3, 4, 5 and 6 illustrate the wavelet coherence and phase difference results of RV, RS, RK, and SJV. For simplicity, here we only present the realized-moment-based time–frequency dependence between bitcoin and other cryptocurrencies.^11^ Among the numerous cryptocurrencies, Bitcoin is the first decentralized cryptocurrency (Borri and Shakhnov 2020). Bitcoin has become the most popular (Kristoufek 2018) and has the largest market capitalization ($701.95B).^12^ We categorize the smallest scale of 2–8 days as the short-term range. The frequency bands of 8–32 days are classified as medium-term whereas the time scales of more than 32 days are deemed as the long-term range. Specifically, the scales of 128 and 256 days correspond to half and one year. The horizontal axis represents the time horizons. The strength of the dependence is measured by the color bar on the right side of the figures, that is, the red (blue) region represents the high (low) comovement. For robustness, we mainly focus on the significant region (5% significance level) in black contours. The phase difference represented by directional arrows in the significant regions is used to identify the lead-lag relationships between cryptocurrencies. Accordingly, the right (left) direction of the arrows denotes the positive (negative) dependence. The right up or left down direction denotes that BTC leads other cryptocurrencies while the left up or right down direction indicates that other cryptocurrencies lead BTC.

Generally, the wavelet coherence of RV between BTC and other cryptocurrencies is stronger than other realized higher-order moments (RS, RK) and signed jump variation (SJV) over most of the sample period and across different frequency bands. Notably, the nature (positive or negative) and the strength of the time–frequency comovement between BTC and other selected cryptocurrencies tend to be time-dependent and frequency-dependent (Ahmed 2022). Furthermore, the strength and direction of the time–frequency comovement vary across realized moments. In terms of RV comovement, BTC exhibits higher coherence with Dash during 2019 and 2020 at the frequency band of 16–32 days. The phase arrows mainly point to the right down indicating that Dash leads BTC in this time–frequency domain. However, the phase arrows point to right up in the first half of 2021 over the period of 128 days indicating that BTC leads Dash. At the frequency band of 8–128 days, EOS has higher coherence with BTC, primarily in 2019 and 2020. The dependence between BTC and EOS is also strong over a 4-to-32-day period between the end of 2021 and the beginning of 2022. Notably, BTC has greater wavelet coherence with ETH than any other cryptocurrency. This finding is consistent with the findings of Yousaf and Ali (2020), Ji et al. (2019), and Özdemir (2022). This indicates that the comovement effects between BTC and ETH are relatively stronger between mid-2020 and early 2022 in the 4-to-128-day frequency band. BTC exhibits greater comovement with LTC over the entire sample period at two frequency bands: 4–32 days and 100–200 days. The majority of the phase arrows point to right up, indicating that BTC leads LTC in the long run. Furthermore, between early 2020 and mid-2021, BTC exhibits strong coherence with BAT at the frequency band of 64–128 days. Other short-lived significant regions are scattered across the frequency range of 4–32 days. The phase arrows are mostly pointing to the right, indicating positive correlations between cryptocurrencies, but the lead-lag relationships between BTC and BAT are mixed. Similarly, Qiao et al. (2020) discovered that Bitcoin primarily leads other cryptocurrencies and has positive relationships with them.

Regarding the realized higher-moment wavelet coherence, we find that BTC still exhibits relatively strong comovement with ETH at the frequency band of 4–64 days. Besides, the strength of the comovement of RS and RK between BTC and other cryptocurrencies is weaker than that of RV. The phase arrows in significant regions mainly point to the right, implying that the daily realized higher-order moments of those cryptocurrencies move synchronously in the same direction, with the zero-phase difference (Ahmed 2022). As for the RS dependence, BTC only exhibits higher coherence with Dash in Q4 2022 at the frequency band of 8–16 days and at the end of 2021 across the scale of 16–32 days. BTC shows higher comovement with EOS at the frequency band of 4–32 days after the outbreak of COVID-19 in December 2019. The phase arrows mostly direct to right up, indicating that BTC is ahead of EOS. Other short-lived significant regions with a period of 4–16 days can be found, primarily in 2021. Notably, over most sample periods, BTC exhibits strong comovement with ETH, primarily at the frequency band of 4–32 days. However, the directions of phase arrows, on the other hand, vary. LTC shows higher coherence with BTC in Q1 2021 at the frequency band of 32–64 days, as well as in Q2 2021 across the time scale of 4–16 days. The right downward arrows show that LTC is ahead of BTC. Furthermore, following the COVID-19 pandemic, BTC exhibits strong comovement with BAT at the frequency band of 48–80 days. Other short-lived significant regions are located in 2020 and 2021 over the course of 4–32 days.

The RK wavelet coherence reveals the comovement between the probabilities of the occurrence of extreme risk events in cryptocurrencies. This extreme risk comovement among cryptocurrencies could be explained by the joint exposure to systemic sources of uncertainty like major crises (Ahmed 2022). Specifically, the RK comovement between BTC and Dash is strong in 2020 and the first half of 2022, mainly at the frequency band of 16–64 days. Noteworthily, Dash leads BTC in the second half of 2020. The significant regions of wavelet coherence between BTC, EOS, and LTC are scattered in different areas. The right upward oriented arrows in significant regions indicate that BTC leads EOS and LTC. BTC exhibits relatively stronger comovement with ETH mainly at two frequency bands: 4–16 days (over the most sample period) and 32–64 days (in the second half of 2020 and the first half of 2021). BAT exhibits higher coherence with BTC in the first half of 2021 at the frequency band of 16–64 days. BTC also leads BAT in this time–frequency domain. Similarly, Tiwari et al. (2020) asserted that strong extreme dependence exists between BTC and other main cryptocurrencies. Ahn (2022) also confirmed the existence of a strong extreme movement in cryptocurrency returns.

We further explored the jump^13^ comovement between BTC and other cryptocurrencies. The wavelet coherence results of SJV are illustrated in Fig. 6. Generally, BTC shows stronger comovement with other cryptocurrencies, mainly at the scale of 4–16 days. This means that jump information in cryptocurrency markets tends to move in the same direction over a shorter period. BTC also outperforms other cryptocurrencies in the majority of sample intervals. However, between mid-2020 and mid-2021, EOS and BAT outperform BTC in the frequency band of 128–256 days. According to Walid et al. (2021), intraday jumps in Bitcoin and Ethereum are quite common, and co-jumps among cryptocurrencies are associated with news releases. Ma and Luan (2022) discovered that the co-jumps can be interpreted as a sign of a cryptocurrency market crash.

## Realized moment connectedness results

In this section, we use the novel time-varying parameter vector autoregression (TVP-VAR) based frequency connectedness approach proposed by Chatziantoniou et al. (2021) to quantify the moment-based connectedness among selected cryptocurrencies. Based on this novel method, we can quantify the connectedness from the time-domain (Diebold and Yilmaz 2012, 2014) and the frequency-domain perspective, that is, considering the connectedness on diverse investment horizons (Baruník and Křehlík 2018). We start from the averaged connectedness analysis based on the connectedness tables. Then, we present the net-pairwise connectedness network to visualize the net-pairwise risk transmissions among cryptocurrencies. Finally, we conducted a dynamic analysis of the connectedness, including the dynamic total, net, and net-pairwise connectedness.

### Averaged connectedness analysis

Tables 3, 4, 5 and 6 present the averaged connectedness results of RV, RS, RK, and SJV, respectively. And notably, each square of the connectedness table presents four numerical results: DY connectedness, short-term (1–7 days), medium-term (7–30 days), and long-term (30-Inf days) connectedness results. The frequency bands are designed according to Kumar et al. (2022).^14^ The sum of the last three numerical values (i.e., frequency connectedness results) is equal to the first numerical value (time-domain connectedness result). Accordingly, the total connectedness indices among cryptocurrencies are 67.31% (RV), 40.99% (RS), 49.99% (RK), and 50.12% (SJV). The total connectedness of RV is higher than that of RS and RK, suggesting that the volatility transmissions among crypto markets are stronger than the higher-order moments. This finding concurs with that of Hasan et al. (2021) and Yi et al. (2018). Bouri et al. (2021a) also found that the realized volatility spillovers are higher than the realized skewness and kurtosis spillovers.

**Table 3:** Averaged connectedness table-RV

	BTC	Dash	EOS	ETH	LTC	BAT	FROM
BTC	26.72 (16.28) [7.23] {3.21}	8.53 (4.72) [2.23] {1.58}	10.94 (6.27) [2.96] {1.71}	21.19 (13.38) [5.67] {2.14}	20.08 (12.51) [5.51] {2.05}	12.54 (8.19) [2.99] {1.36}	73.28 (45.07) [19.37] {8.84}
Dash	10.05 (4.31) [2.46] {3.27}	44.71 (21.51) [14.05] {9.15}	15.68 (7.21) [4.67] {3.79}	9.09 (5.38) [2.09] {1.61}	6.23 (3.25) [1.47] {1.51}	14.25 (7.52) [4.01] {2.72}	55.29 (27.68) [14.70] {12.91}
EOS	12.08 (6.75) [2.93] {2.39}	13.47 (7.54) [3.30] {2.62}	34.04 (20.81) [8.94] {4.29}	11.13 (7.69) [2.20] {1.24}	8.72 (5.39) [1.99] {1.34}	20.55 (13.12) [5.02] {2.41}	65.96 (40.49) [15.45] {10.01}
ETH	21.24 (13.35) [5.65] {2.24}	9.04 (5.33) [2.33] {1.38}	10.86 (6.92) [2.80] {1.14}	27.99 (17.68) [7.32] {2.99}	20.20 (12.37) [5.64] {2.20}	10.67 (7.63) [2.23] {0.81}	72.01 (45.60) [18.65] {7.77}
LTC	22.26 (12.80) [6.31] {3.14}	6.85 (4.35) [1.71] {0.79}	9.60 (6.73) [2.15] {0.72}	22.68 (12.92) [6.31] {3.46}	29.84 (17.47) [8.29] {4.08}	8.77 (6.41) [1.79] {0.57}	70.16 (43.21) [18.26] {8.69}
BAT	14.91 (9.29) [3.81] {1.81}	12.06 (7.54) [3.17] {1.35}	20.85 (12.51) [5.94] {2.40}	11.22 (7.98) [2.30] {0.94}	8.11 (5.15) [2.07] {0.89}	32.85 (20.88) [8.80] {3.17}	67.15 (42.48) [17.29] {7.38}
TO	80.54 (46.51) [21.17] {12.86}	49.94 (29.49) [12.73] {7.72}	67.93 (39.63) [18.53] {9.77}	75.31 (47.36) [18.57] {9.38}	63.35 (38.68) [16.68] {7.99}	66.78 (42.86) [16.04] {7.88}	TCI
NET	7.27 (1.45) [1.80] {4.02}	− 5.35 (1.81) [− 1.97] {− 5.19}	1.97 (− 0.86) [3.09] {− 0.25}	3.30 (1.76) [− 0.08] {1.62}	− 6.82 (− 4.53) [− 1.59] {− 0.69}	− 0.37 (0.38) [− 1.25] {0.50}	67.31 (40.76) [17.29] {9.27}

The numerical results are obtained by employing Chatziantoniou et al.′s (2021) novel TVP-VAR based frequency connectedness approach. The results are based on a TVP-VAR model with the optimal lag length (lag order = 2) and a forecast error variance decomposition with 100 steps ahead. The values in the upper left corner of each square represent the time-domain connectedness results obtained using the traditional DY connectedness approach. The values in parentheses (), square brackets [], and curly brace {} denote the short-term (1–7 days, weekly), medium-term (7–30 days, monthly), and long-term (more than 30 days) frequency connectedness results. The column ‘FROM’ indicates the total connectedness received by market i from all other markets. The row ‘TO’ indicates the total connectedness transmitted by a market i to all other markets. The row ‘NET’ denotes the net connectedness results. The positive/negative ‘NET’ values represent the net-transmitter/net-recipient of the connectedness. ‘TCI’ located in the bottom right denotes the total connectedness index, which measures the total spillover effects within the system

**Table 4:** Averaged connectedness table-RS

	BTC	Dash	EOS	ETH	LTC	BAT	FROM
BTC	47.18 (40.80) [5.12] {1.26}	3.69 (3.19) [0.41] {0.10}	4.65 (3.92) [0.58] {0.14}	25.87 (22.33) [2.83] {0.71}	16.95 (14.12) [2.26] {0.57}	1.66 (1.28) [0.31] {0.08}	52.82 (44.84) [6.38] {1.60}
Dash	5.06 (4.45) [0.49] {0.12}	67.90 (57.40) [8.39] {2.11}	16.99 (14.73) [1.81] {0.44}	2.12 (1.74) [0.30] {0.08}	2.05 (1.75) [0.24] {0.06}	5.89 (5.20) [0.56] {0.14}	32.10 (27.87) [3.40] {0.83}
EOS	5.63 (4.84) [0.64] {0.16}	16.27 (14.61) [1.34] {0.32}	62.02 (52.74) [7.42] {1.86}	3.31 (2.75) [0.44] {0.11}	4.15 (3.49) [0.54] {0.13}	8.62 (7.29) [1.06] {0.27}	37.98 (32.97) [4.02] {1.00}
ETH	26.31 (23.34) [2.39] {0.58}	1.64 (1.45) [0.15] {0.04}	2.66 (2.35) [0.25] {0.06}	47.91 (41.50) [5.13] {1.28}	20.18 (16.93) [2.60] {0.65}	1.29 (1.06) [0.19] {0.05}	52.09 (45.13) [5.59] {1.37}
LTC	18.74 (16.59) [1.73] {0.42}	1.67 (1.34) [0.26] {0.07}	3.41 (2.93) [0.38] {0.09}	21.63 (17.95) [2.93] {0.75}	53.29 (44.91) [6.69] {1.69}	1.27 (0.96) [0.25] {0.06}	46.71 (39.77) [5.56] {1.38}
BAT	2.60 (2.33) [0.21] {0.05}	6.62 (5.72) [0.71] {0.18}	10.55 (9.53) [0.83] {0.19}	2.43 (2.08) [0.28] {0.07}	2.05 (1.78) [0.22] {0.05}	75.76 (64.22) [9.22] {2.31}	24.24 (21.44) [2.25] {0.54}
TO	58.33 (51.55) [5.47] {1.32}	29.89 (26.30) [2.88] {0.71}	38.26 (33.47) [3.86] {0.93}	55.35 (46.86) [6.77] {1.72}	45.38 (38.07) [5.85] {1.46}	18.74 (15.77) [2.37] {0.59}	TCI
NET	5.51 (6.71) [− 0.91] {− 0.28}	− 2.21 (− 1.57) [− 0.52] {− 0.12}	0.27 (0.50) [− 0.16] {− 0.07}	3.26 (1.73) [1.18] {0.34}	− 1.33 (− 1.69) [0.29] {0.08}	− 5.50 (− 5.67) [0.11] {0.05}	40.99 (35.34) [4.53] {1.12}

See Table 3

**Table 5:** Averaged connectedness table-RK

	BTC	Dash	EOS	ETH	LTC	BAT	FROM
BTC	38.03 (33.14) [3.93] {0.97}	2.88 (2.69) [0.16] {0.03}	3.64 (3.41) [0.19] {0.04}	30.86 (27.11) [3.02] {0.72}	22.51 (19.54) [2.40] {0.57}	2.08 (1.96) [0.10] {0.02}	61.97 (54.72) [5.86] {1.38}
Dash	4.09 (3.38) [0.55] {0.16}	58.78 (45.22) [10.73] {2.82}	17.49 (13.15) [3.43] {0.91}	4.23 (3.65) [0.46] {0.12}	3.20 (2.65) [0.43] {0.12}	12.22 (9.78) [1.95] {0.48}	41.22 (32.62) [6.82] {1.78}
EOS	3.81 (2.92) [0.69] {0.19}	19.03 (13.87) [4.08] {1.07}	56.8 (44.11) [10.06] {2.64}	3.28 (2.55) [0.57] {0.16}	3.74 (2.91) [0.65] {0.18}	13.34 (10.21) [2.49] {0.64}	43.2 (32.47) [8.48] {2.24}
ETH	29.46 (26.32) [2.55] {0.59}	2.69 (2.53) [0.13] {0.03}	3.59 (3.27) [0.26] {0.06}	37.32 (32.96) [3.51] {0.84}	24.56 (21.60) [2.40] {0.56}	2.38 (2.26) [0.10] {0.02}	62.68 (55.98) [5.45] {1.26}
LTC	23.64 (20.42) [2.58] {0.64}	2.20 (1.84) [0.29] {0.07}	3.30 (2.79) [0.41] {0.10}	26.94 (23.16) [3.03] {0.75}	42.63 (36.32) [5.06] {1.25}	1.29 (1.05) [0.20] {0.05}	57.37 (49.26) [6.50] {1.61}
BAT	3.20 (2.72) [0.38] {0.10}	12.12 (10.28) [1.47] {0.37}	11.77 (9.64) [1.68] {0.45}	3.79 (3.35) [0.35] {0.09}	2.64 (2.20) [0.35] {0.09}	66.48 (57.76) [6.99] {1.73}	33.52 (28.19) [4.23] {1.10}
TO	64.2 (55.76) [6.75] {1.69}	38.92 (31.22) [6.13] {1.57}	39.78 (32.26) [5.98] {1.55}	69.09 (59.82) [7.44] {1.83}	56.65 (48.91) [6.22] {1.52}	31.31 (25.27) [4.83] {1.21}	TCI
NET	2.24 (1.04) [0.89] {0.31}	− 2.30 (− 1.4) [− 0.69] {− 0.21}	− 3.42 (− 0.22) [− 2.51] {− 0.69}	6.41 (3.84) [1.99] {0.58}	− 0.72 (− 0.35) [− 0.28] {− 0.09}	− 2.21 (− 2.92) [0.60] {0.11}	49.99 (42.21) [6.22] {1.56}

See Table 3

**Table 6:** Averaged connectedness table-SJV

	BTC	Dash	EOS	ETH	LTC	BAT	FROM
BTC	40.26 (35.27) [3.99] {1.00}	6.21 (5.16) [0.70] {0.35}	9.39 (8.19) [0.96] {0.25}	22.40 (19.87) [2.02] {0.50}	15.64 (13.97) [1.35] {0.33}	6.10 (5.11) [0.70] {0.30}	59.74 (52.29) [5.73] {1.73}
Dash	6.90 (5.98) [0.74] {0.17}	64.85 (54.76) [8.01] {2.17}	10.98 (9.46) [1.22] {0.31}	6.99 (6.03) [0.77] {0.18}	7.11 (6.06) [0.84] {0.21}	3.17 (2.39) [0.55] {0.24}	35.15 (29.93) [4.12] {1.11}
EOS	10.54 (9.16) [1.11] {0.27}	9.57 (7.90) [1.19] {0.48}	45.70 (39.64) [4.85] {1.21}	12.23 (10.67) [1.25] {0.31}	13.91 (11.99) [1.53] {0.39}	8.04 (5.88) [1.60] {0.56}	54.30 (45.61) [6.68] {2.01}
ETH	21.48 (18.72) [2.21] {0.54}	5.62 (4.47) [0.78] {0.37}	10.14 (8.72) [1.13] {0.30}	39.72 (34.76) [3.98] {0.98}	17.43 (15.04) [1.91] {0.48}	5.61 (4.33) [0.93] {0.35}	60.28 (51.28) [6.96] {2.04}
LTC	15.46 (13.36) [1.69] {0.42}	6.78 (5.44) [0.92] {0.42}	13.23 (11.16) [1.65] {0.43}	18.93 (16.33) [2.08] {0.52}	39.86 (34.04) [4.65] {1.17}	5.74 (4.17) [1.14] {0.43}	60.14 (50.45) [7.48] {2.21}
BAT	7.43 (6.42) [0.81] {0.20}	3.91 (2.70) [0.79] {0.41}	8.39 (6.84) [1.22] {0.33}	7.39 (6.23) [0.92] {0.24}	3.99 (3.28) [0.56] {0.15}	68.89 (54.45) [11.37] {3.07}	31.11 (25.46) [4.31] {1.34}
TO	61.82 (53.65) [6.56] {1.61}	32.09 (25.67) [4.38] {2.03}	52.14 (44.36) [6.18] {1.60}	67.93 (59.13) [7.04] {1.75}	58.08 (50.34) [6.19] {1.56}	28.66 (21.88) [4.91] {1.87}	TCI
NET	2.07 (1.36) [0.84] {− 0.12}	− 3.06 (− 4.25) [0.26] {0.93}	− 2.16 (− 1.25) [− 0.50] {− 0.40}	7.65 (7.85) [0.09] {− 0.29}	− 2.06 (− 0.11) [− 1.29] {− 0.65}	− 2.45 (− 3.59) [0.61] {0.54}	50.12 (42.50) [5.88] {1.74}

See Table 3

In terms of frequency connectedness results, total connectedness is primarily transmitted in the short-term range (1–7 days), implying that the highest frequency (short-term) component dominates total connectedness. This finding is consistent with those of Kumar et al. (2022) and Mensi et al (2021). For example, the total connectedness of RV is 67.31%, with 40.76% attributed to short-term factors, 17.29% attributed to medium-term factors, and only 9.27% attributed to long-term movements. Furthermore, the total connectedness of RK is 49.99%, with decomposed components of 42.21% (1–7 days), 6.22% (7–30 days), and 1.56% (30-Inf days). This implies that the majority of cryptocurrency investors are influenced by short-term factors and behave similarly at short time scales (Kang et al. 2019).

When we consider the connectedness transmitted ‘TO’ to the entire system, we discover that BTC contributes the most to the system at all realized moments, followed by LTC and ETH. Indeed, BTC is the most popular and largest cryptocurrency, while ETH is the second-largest market after BTC (Mensi et al. 2021). This finding is consistent with the findings of Polat and Günay (2021), Mensi et al. (2021), Moratis (2021), and Ji et al (2019). Furthermore, Wang and Ngene (2020) discovered that BTC dominates price movements in both bear and bull markets. In terms of total connectedness received ‘FROM’ the entire system, BTC, ETH and LTC continue to receive relatively higher connectedness at all realized moments.

Furthermore, the diagonal elements measure the connectedness caused by their own shocks. We find that the diagonal elements of the RV connectedness table are mostly around 30% with the exception, Dash (44.71%), implying that most of the volatility connectedness is caused by the cross-market shocks from other crypto markets. As for the higher-order moment connectedness, we find that only the diagonal elements of the BTC and ETH are less than 50% while others are above half, with the highest values for BAT (75.76%, 66.48%, and 68.89%), followed by Dash. This indicates that most of the connectedness can be attributed to the early shocks in BAT. This also reveals the less important roles of BAT and Dash in the risk transmission system.

The connectedness table’s last row (‘NET’) presents the net spillovers. Based on the net connectedness results, one can identify the role of the cryptocurrency (i.e. the positive value denotes the net transmitter while the negative value indicates the net receiver) that offers references for investors and portfolio managers to optimize their investment strategies and monitor the portfolio risks (Mensi et al. 2021). Accordingly, BTC is the largest connectedness net-transmitter of RV (7.27%) and RS (5.51%). This finding concurs with that of Ji et al. (2019) who found that Bitcoin acts as the largest net transmitter of volatility spillovers. Koutmos (2018) also concluded that Bitcoin acts as a pivot in the volatility connectedness network. Antonakakis et al. (2019) also found that Bitcoin is the most significant transmitter of shocks in the cryptocurrency market, followed by Ethereum. Wang and Ngene (2020) found that BTC dominates the cryptocurrency market by inducing more rapid and destabilizing effects on other cryptocurrencies. However, ETH acts as the largest net spillover transmitter when considering the RK (6.41%) and SJV (7.65%) connectedness, followed by BTC. This may suggest that the extreme risks and jumps in ETH and BTC will exert significant net shocks to the entire crypto-system. Raza et al. (2022) also found that Bitcoin and Ethereum are the highest transmitters among others. Notably, LTC is the largest net receiver of the RV (− 6.82%) connectedness, followed by Dash (− 5.35%). However, BAT and EOS tended to be the largest net recipients of the RS (− 5.50%) and RK (− 3.42%) connectedness, respectively. Besides, Dash acts as the largest net receiver of the SJV (− 3.06%) connectedness. Moreover, the net connectedness is also dominated by the short-term (high-frequency, 1–7 days) movements in cryptocurrencies at all realized moments.

Furthermore, we also quantified the spillovers of RSV-P, RSV-N, RHS, and RHK among the cryptocurrencies. As shown in Appendix Tables 7, 8, 9 and 10, the total connectedness indices are 66.67% (RSV-P), 70.14% (RSV-N), 38.29% (RHS), and 46.21% (RHK). The total connectedness of realized hyper-skewness and hyper-kurtosis are near to the realized skewness and kurtosis connectedness. These results highlight again the higher-order moment risk connectedness among cryptocurrencies. The spillover of the realized negative semi-variance is higher than the realized positive semi-variance, suggesting the significant asymmetrical features in volatility connectedness. This finding is supported by Ji et al. (2019), who asserted that the negative return connectedness is significantly stronger than the positive. However, we portray the asymmetry effects on connectedness from the realized semi-variance perspective. The investment sentiment may cause asymmetric features in volatility connectedness among cryptocurrencies. That is to say, the negative sentiment is more easily spread in cryptocurrency markets, thus increasing the total connectedness. Similarly, the short-term (1–7 days) interactions dominate the total connectedness indices of RSV-P, RSV-N, RHS, and RHK. This highlights that investors with short investment horizons should stay high alert to the connectedness in the short term. BTC and ETH still contribute (receive) the highest RSV, RHS, and RHK connectedness ‘TO’ (‘FROM’) to the entire system. The diagonal element for BTC had the lowest RSV-P and RSV-N connectedness. Regarding the RHS and RHK connectedness, ETH shows the lowest diagonal values, suggesting that the higher-order moment risk connectedness of ETH is mostly caused by the interactions within the entire cryptocurrency system and ETH has more interacted with other cryptocurrencies. This finding is in line with the RV, RS, RK, and SJV connectedness.

Turning to the net connectedness, BTC (6.55%), Dash (2.92%), and EOS (2.12%) are the net transmitters, whereas LTC (− 9.92%) is the largest net recipient of the RSV-P connectedness, followed by BAT (− 1.17%) and ETH (− 0.51%). As for the RSV-N connectedness, BTC (6.32%) acted as the largest net transmitter, followed by ETH (3.77%). LTC (− 6.04%) still acted as the largest net receiver. Regarding the RHS and RHK connectedness, ETH transmits the highest net spillovers to the whole system, followed by BTC. Notably, LTC and BAT change to the net transmitters of the RHS and RHK connectedness. Only Dash and EOS act as the net receivers of the RHS and RHK spillovers. Dash is also the largest net recipient of the RHK connectedness (− 13.5%). In sum, the net connectedness results of RSV, RHS, and RHK partially support the RV, RS, RK, and SJV connectedness results but demonstrate some noteworthy differences. This also implies that the risk connectedness among cryptocurrencies may vary with the order of the realized moments.

### Directional net-pairwise connectedness network

The connectedness table presents the total and net connectedness at different realized moments among selected cryptocurrencies. However, this ignores the directional net-pairwise connectedness between two specific crypto markets. Thus, we further construct the directional net-pairwise connectedness networks to extend our analysis.

**Fig. 7:**
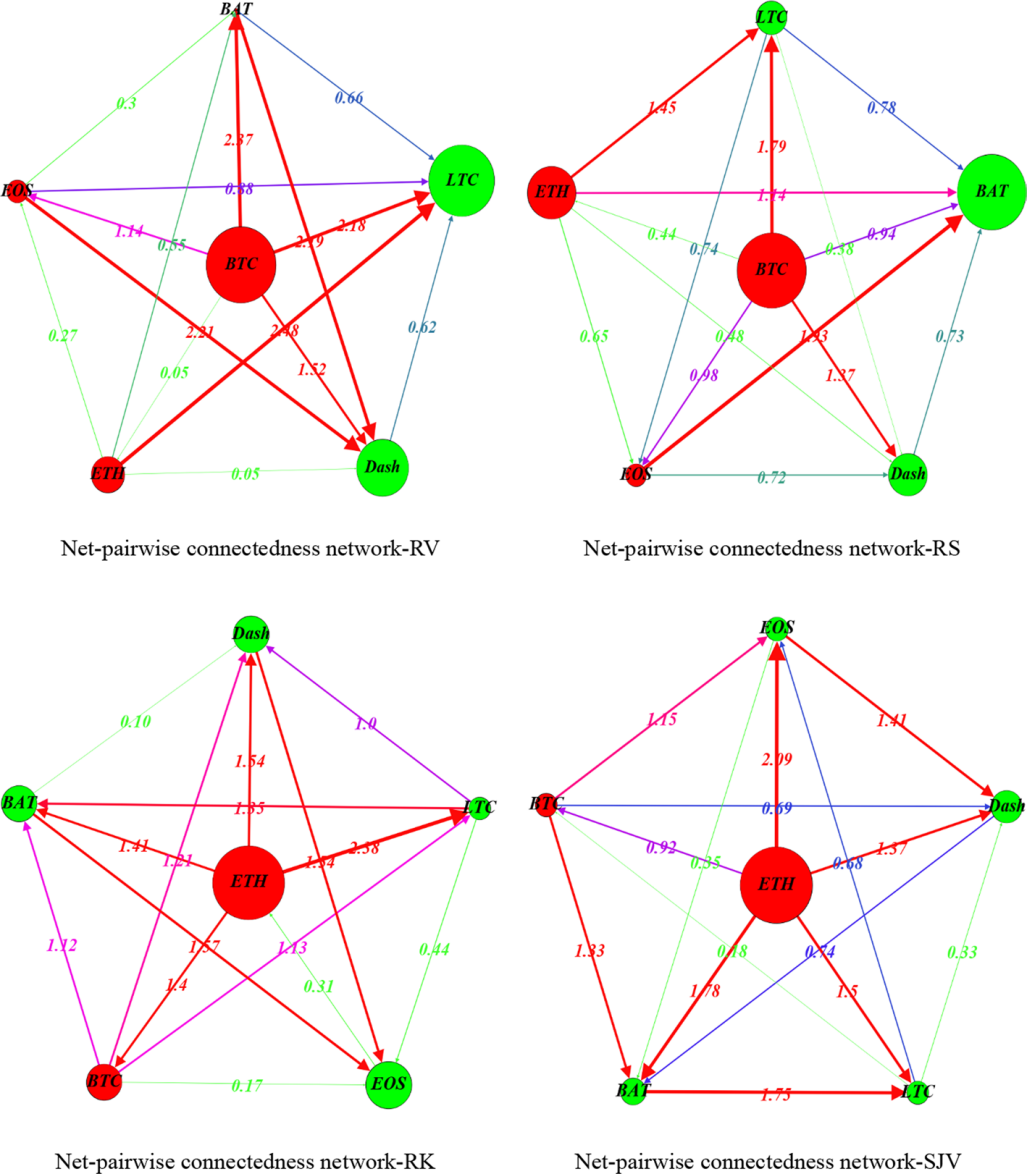
Directional net-pairwise connectedness networks

Accordingly, Fig. 7 presents the net-pairwise networks of RV, RS, RK, and SJV connectedness.^15^ Appendix Fig. 21 shows the net-pairwise connectedness networks of RSV-P, RSV-N, RHS, and RHK connectedness. Notably, as the largest net transmitter, BTC dominates the net-pairwise spillover of RV and RS connectedness networks, while ETH dominates the RK and SJV net-pairwise connectedness. Koutmos (2018) found that Bitcoin still acts as the leader in terms of shock transmissions. Besides, BTC contributed the highest RV connectedness to BAT (2.37%) and LTC (2.18%). Kumar and Anandarao (2019) showed that Bitcoin has significant volatility spillovers to Ethereum and Litecoin. Bitcoin is the most prominent and influential among cryptocurrencies. Further, ETH transmits the highest RV spillovers to LTC (2.48%). Katsiampa (2019) also concluded that there is only a uni-directional spillover of shocks from Ethereum towards Litecoin. EOS and BAT also contributed relatively higher RV connectedness to Dash (2.21% and 2.19%).

When we consider higher-order moment connectedness, we discover that BAT is the largest net transmitter of RS connectedness. BTC transmits more RS net connectedness than LTC (1.79%) and (1.37%). As the second-largest net transmitter, ETH also provides significant RS connectedness to LTC (1.45%) and BAT (1.14%). Notably, EOS transmits the highest RS connectedness to BAT (1.93%). In terms of RK spillovers, ETH is the network’s leading net transmitter, spreading the most RK connectedness to LTC (2.38%), followed by Dash (1.54%). Another net transmitter, BTC, contributes significantly to Dash (1.21%), LTC (1.13%), and BAT RK connectedness (1.12%). Furthermore, BAT sends significant RK spillovers to EOS (1.57%).

Overall, there is significant higher-order net-pairwise connectedness among the selected cryptocurrencies, implying that these cryptocurrencies experience asymmetric and extreme price movements together (Hasan et al. 2021). This finding may be related to herding and speculative behaviors previously confirmed by King and Koutmos (2021), and Anamika and Subramaniam (2022).

When it comes to SJV connectedness, ETH dominates the network’s net-pairwise spillover and spills significant SJV net connectedness to EOS (2.09%), BAT (1.78%), and LTC (1.50%). BTC also has significant SJV spillovers to BAT (1.33%) and EOS (1.15%). Also, BAT contributes higher SJV spillovers to LTC (1.75%), whereas EOS spreads significant SJV spillovers to Dash (1.41%).

As shown in Appendix Fig. 21, BTC remains the leading net transmitter of both the RSV-P and RSV-N connectedness. BTC, in particular, transmits the highest RSV-P connectedness to LTC (2.75%) and BAT (2.3%). LTC also receives higher RSV-P spillovers from Dash (2.06%), EOS (1.85%), and ETH (1.87%). BTC spills a higher RSV-N connectedness to LTC (2.71%) and EOS (1.54%). Notably, ETH also spreads positive RSV-N spillovers to LTC (2.60%), whereas EOS contributes significant RSV-N connectedness to BAT (2.08%). Furthermore, ETH becomes the leading net transmitter of RHS and RHK connectedness. EOS receives more RHS spillovers from BTC (1.48%), LTC (2.74%), and ETH (2.16%). BAT also transmits the strongest RHS connection to Dash (2.88%). In terms of RHK connectedness, BAT spreads the highest net-pairwise RHK connectedness to Dash (7.07%). EOS also contributes significantly to Dash’s RHK connectedness (3.60%). Furthermore, BTC (3.53%), ETH (2.87%), and LTC (3.08%) spread relatively stronger RHK spillovers to EOS.

### Dynamic total connectedness analysis

**Fig. 8:**
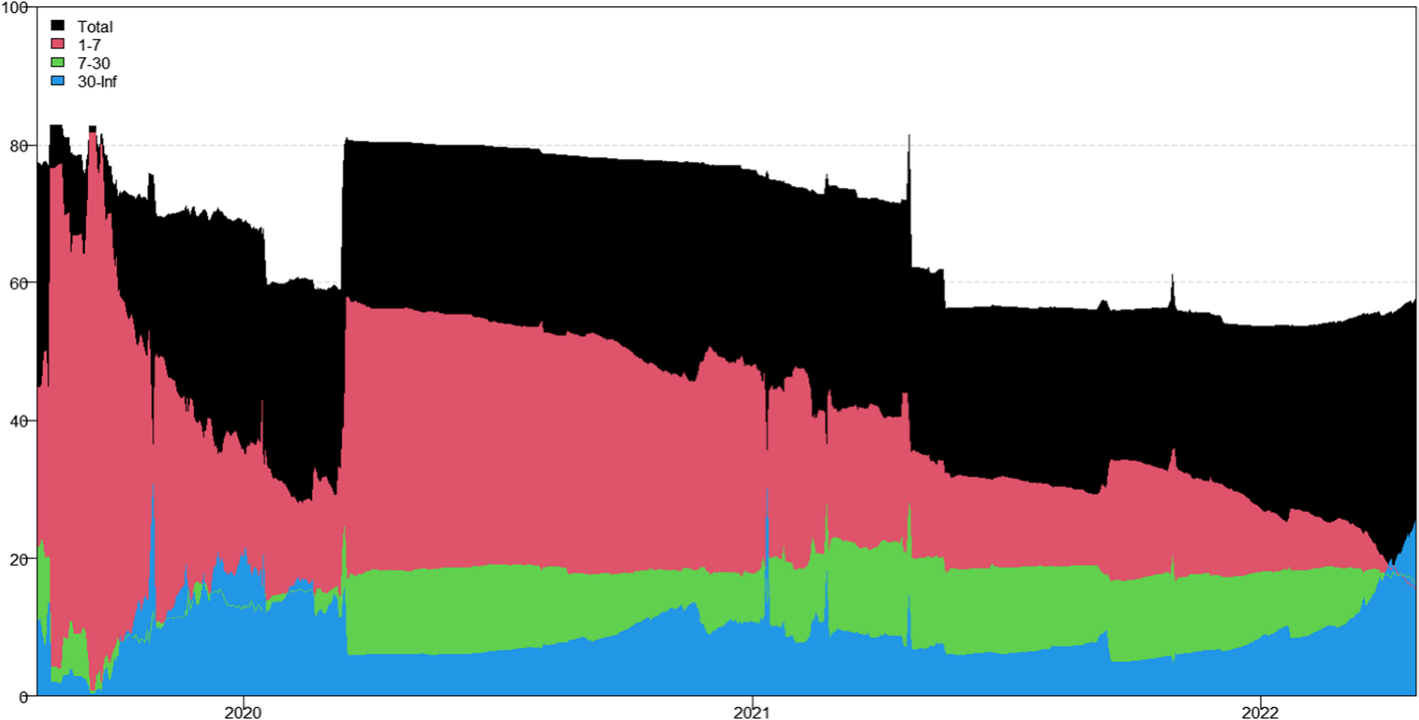
Dynamic total connectedness—realized volatility (RV)

**Fig. 9:**
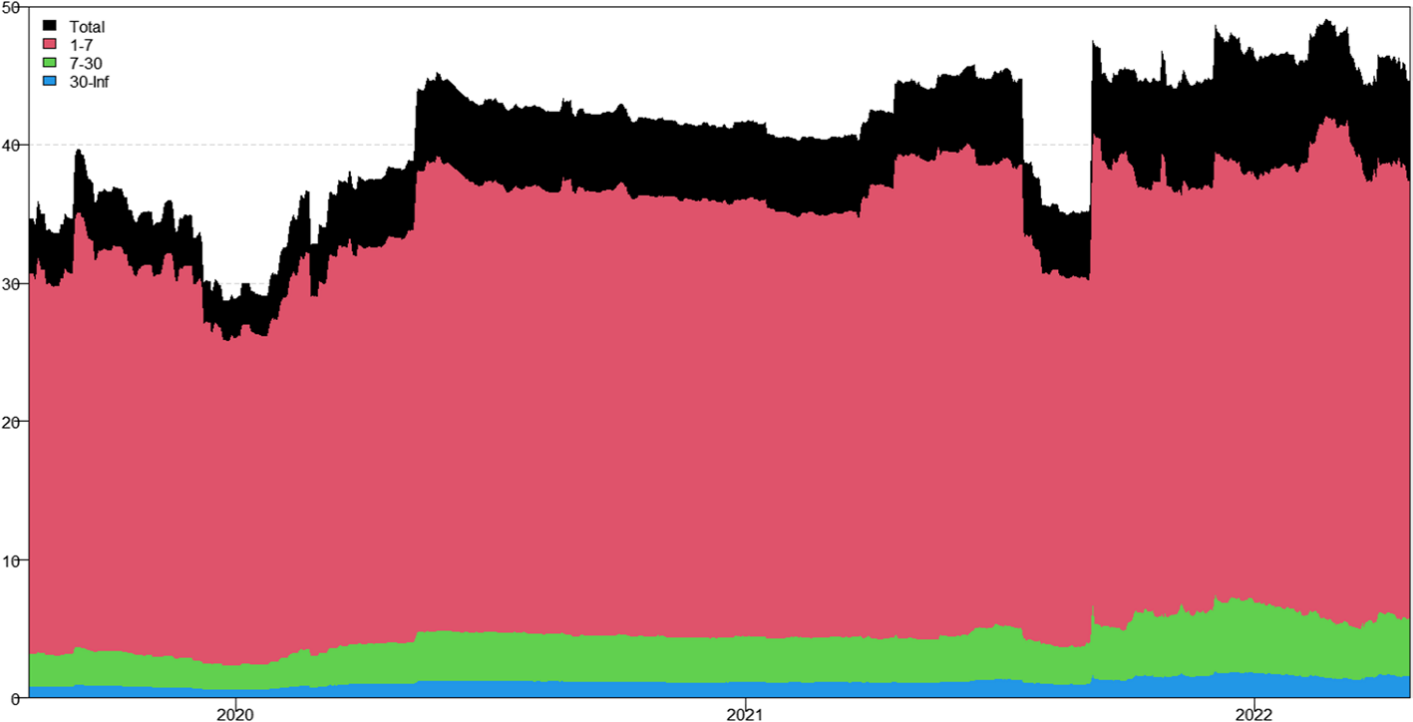
Dynamic total connectedness—realized skewness (RS)

**Fig. 10:**
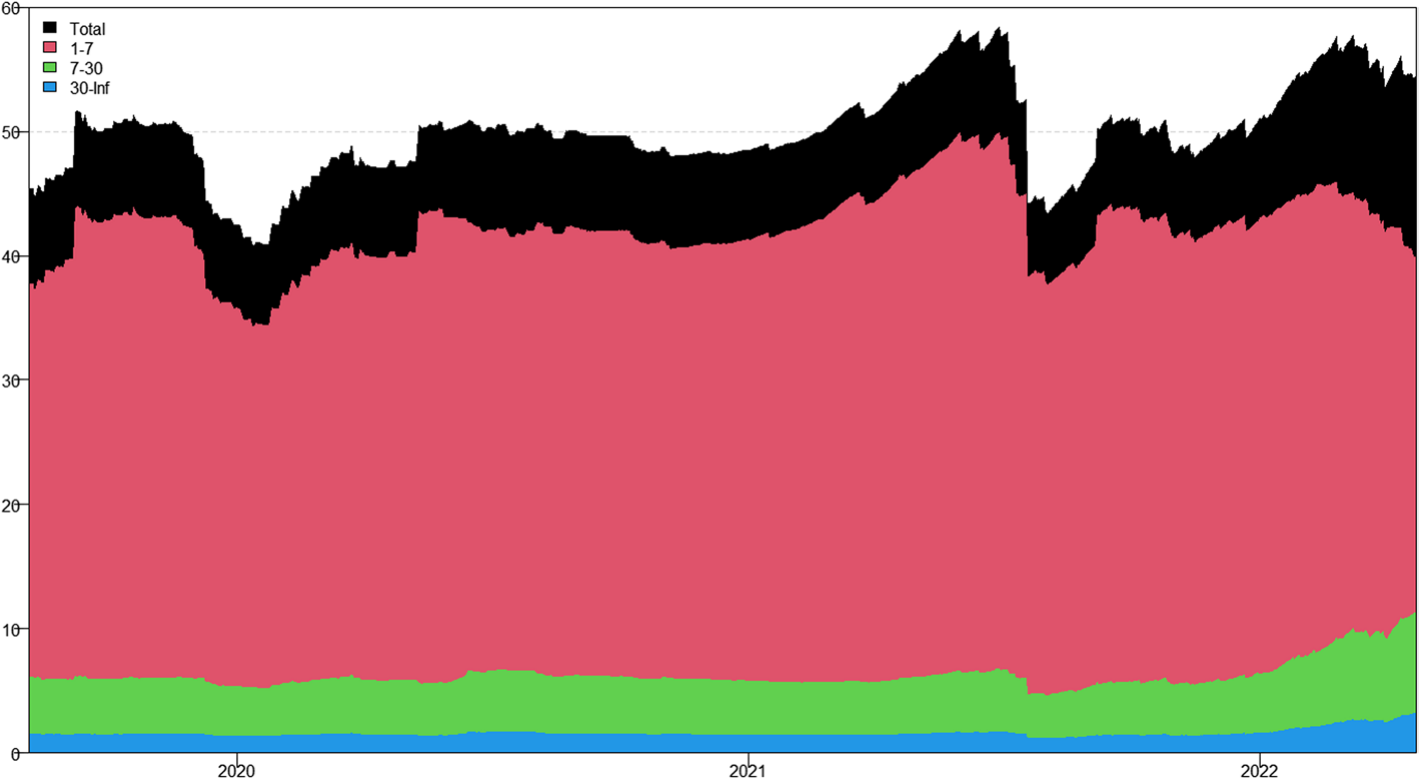
Dynamic total connectedness—realized kurtosis (RK)

**Fig. 11:**
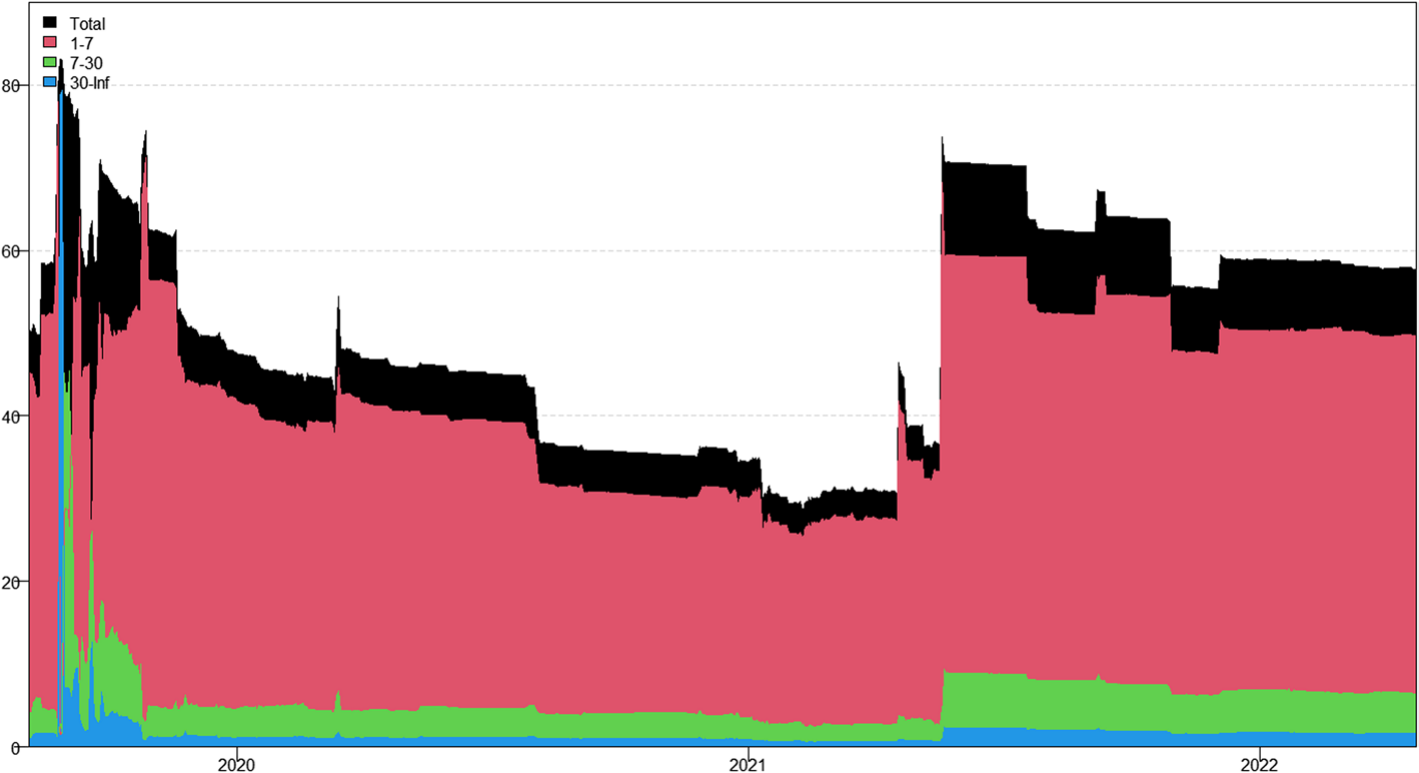
Dynamic total connectedness—signed jump variation (SJV)

The connectedness table and net-pairwise network presented in “Averaged connectedness analysis” and “Directional net-pairwise connectedness network” sections depict the moment-based connectedness among selected cryptocurrencies from a static perspective. This section further portrays the dynamic characteristics of total RV, RS, RK, and SJV connectedness. As illustrated in Figs. 8, 9, 10 and 11, the black shade refers to the total connectedness index, whereas the red, green, and blue shadows denote the total connectedness indices in short-term (1–7 days), medium-term (7–30 days), and long-term (30-Inf days), respectively. We find that the red regions dominate the dynamic total connectedness indices, implying that the total spillovers are mainly transmitted in the short term. This finding agrees with the averaged frequency connectedness results. Besides, the significant time-varying features can be observed in the dynamic total connectedness indices. Kumar et al. (2022) also found that the total dynamic spillover among cryptocurrencies is dominated by the short-term horizon. These findings are also in agreement with the results of Mensi et al. (2021) and Mo et al. (2022).

The dynamic total RV connectedness index, as shown in Fig. 8, oscillates between 50 and 80% and peaks in March 2020, coinciding with the outbreak of the COVID-19 pandemic. This finding is consistent with those of Polat and Günay (2021) and Elsayed et al. (2022), who discovered that spillovers had significantly increased following the official announcement of the COVID-19 pandemic. Lahmiri and Bekiros (2020) concluded that during the COVID-19 pandemic period, cryptocurrency markets became more volatile, and the level of irregularity in cryptocurrency increased significantly. According to Özdemir (2022), the COVID-19 pandemic has resulted in more integrated cryptocurrency markets, stimulating herding behavior among investors. Indeed, Demiralay and Golitsis (2021) also asserted that herding behavior has significantly intensified the interlinkages and risk contagion effects among leading cryptocurrencies during the COVID-19 pandemic period. Following the sharp increase in March 2020, the dynamic total RV connectedness gradually decreases until April 2021, when it fluctuates around 55%.

Regarding the higher-order moment connectedness, the dynamic total RS connectedness index oscillates between 30 and 50%, whereas the total RK connectedness fluctuates between 40 and 60%. The dynamic total RV connectedness indices are mostly higher than the total dynamic RS, RK, and SJV connectedness. This finding supports the results of Bouri et al. (2021a) that systemic volatility risks play a more influential role in terms of spillover magnitude. This may be because volatility transmission is more common while other higher-order moments and jump risk transmission are relatively occasional (Bouri et al. 2021a). Moreover, both the total dynamic RS and RK connectedness indices react remarkably to the COVID-19 pandemic. This finding is consistent with the results of Hasan et al. (2021) that RS and RK connectedness among cryptocurrencies presents a swift increase during the COVID-19 pandemic crisis period. Bouri et al. (2021a) also asserted that the dynamic total realized skewness and kurtosis spillovers tend to intensify during major crises. However, this finding does not support Gomez-Gonzalez et al. (2022) who found that skewness and kurtosis do not exhibit spillover peaks during the pandemic. Another sharp increase in total dynamic RS and RK spillover can be found in September 2021. This finding may be related to the U.S. Treasury sanctioning the cryptocurrency exchange SUEX.^16^ Furthermore, the total dynamic RS and RK connectedness indices illustrate an overall upward trend, suggesting the growing probability of crash risk and exposure to extreme financial events. The dynamic total SJV connectedness index presents unique features. It increased sharply in August 2019, then decreases gradually from 80 to 30%. In May 2021, it rises abruptly from 30 to 70%.

Focusing on the Appendix Figs. 22, 23, 24 and 25, we find that the dynamic total connectedness indices of RSV-P, RSV-N, RHS, and RHK are also time-varying and dominated by short-term movements. The dynamic RSV-P and RSV-N spillover indices show consistent evolving features and both of which are similar to the RV connectedness.

However, notably, the dynamic total RSV-P connectedness is higher than the RSV-N from March to the end of 2020, implying that the realized good volatility spillovers are stronger than bad volatility^17^ during this period. This finding is supported by the results of Mensi et al. (2019b) that the realized good volatility spillovers dominate the realized bad volatility spillovers during some periods. Moreover, the dynamic total connectedness indices of RHS and RHK were in line with the RS and RK connectedness. Both of them are sensitive to the COVID-19 pandemic and are also event dependent. Compared to Hasan et al. (2021), who only depicted the total dynamic RV, RS, and RK spillovers in the time domain, we have offered more in-depth evidence of the total dynamic connectedness in the time as well as the frequency domains. Besides, we also further quantified the total dynamic spillover of the jump, good and bad volatility, and hyper-skewness and hyper-kurtosis.

### Dynamic net connectedness analysis

**Fig. 12:**
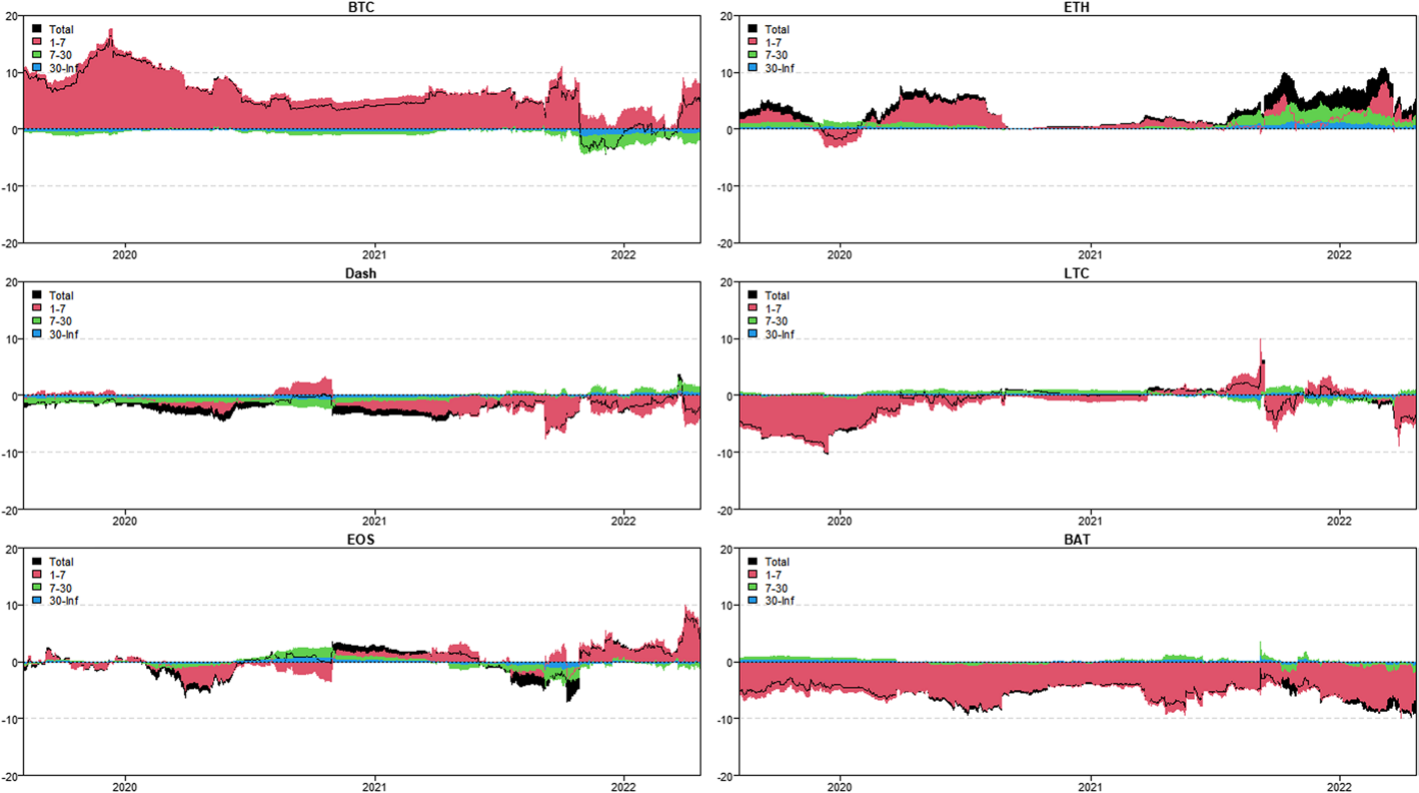
Dynamic net connectedness—realized skewness (RS)

**Fig. 13:**
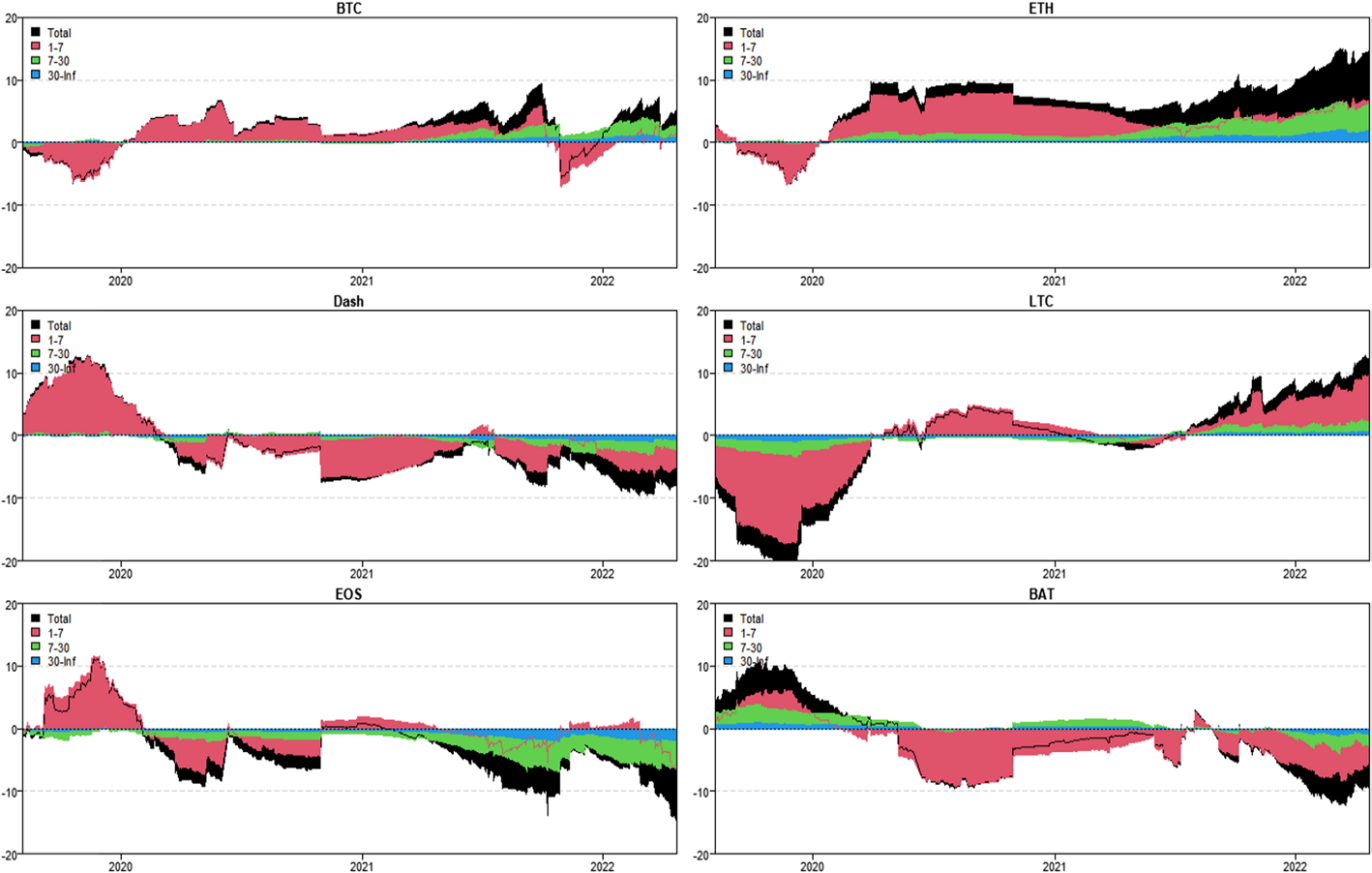
Dynamic net connectedness—realized kurtosis (RK)

**Fig. 14:**
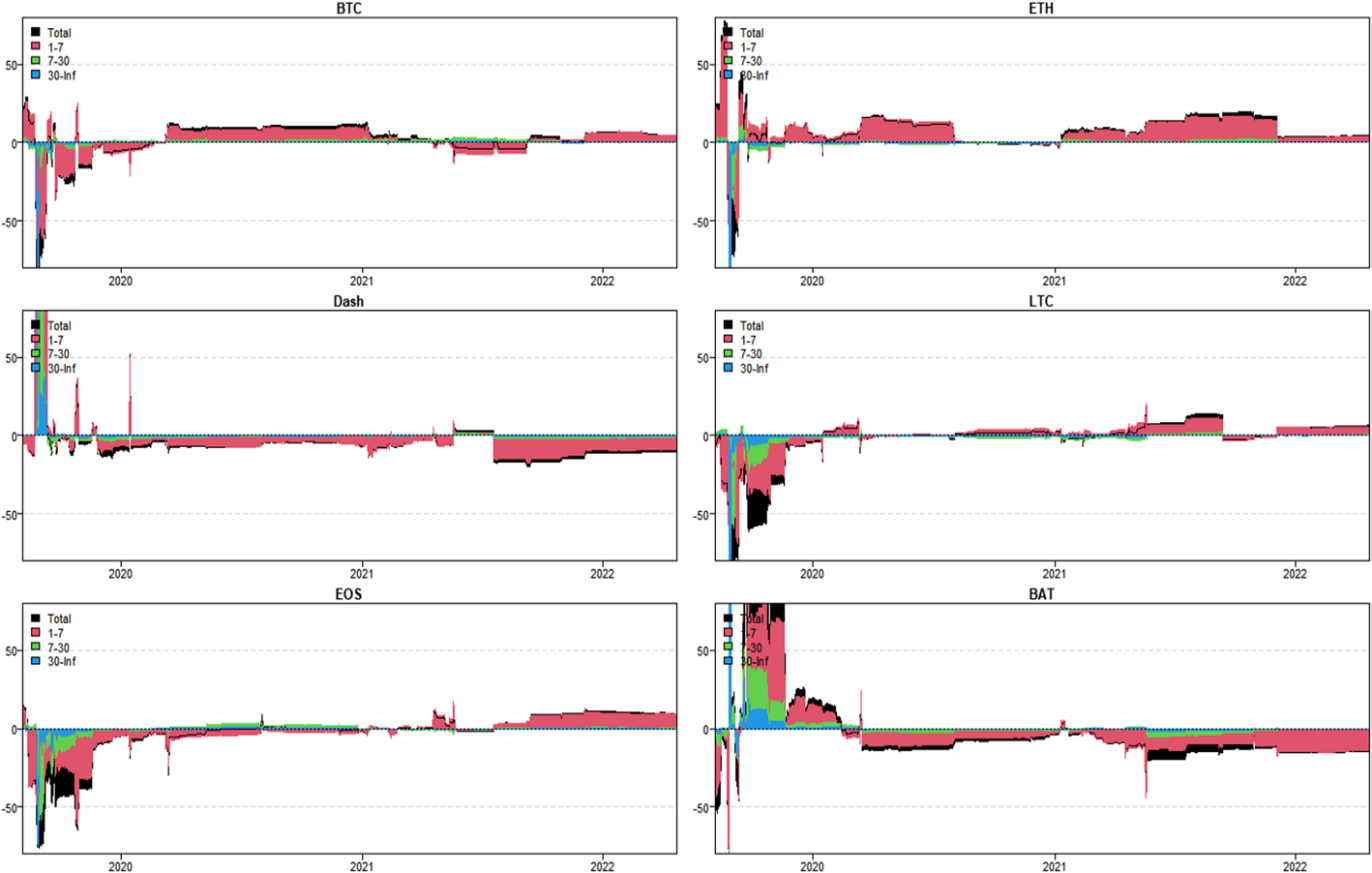
Dynamic net connectedness—signed jump variation (SJV)

To further depict the dynamics of the net spillovers which identify the connectedness role of each market (i.e. the net transmitter or the net receiver), we quantify the net connectedness index of each cryptocurrency, which is presented in Figs. 12, 13 and 14. The positive (negative) spillover index indicates that the cryptocurrency is the net transmitter (recipient). Generally, the dynamic net connectedness indices of the RS, RK, and SJV are also time-varying and are also mainly dominated by red, suggesting that the dynamic net spillovers are largely transmitted in the short-term horizon.

Figure 12 presents the evolution of net-realized skewness connectedness over time. It is distinct that BTC is the net transmitter over the sample period and spreads the strongest skewness spillovers to other cryptocurrencies. ETH is another persistent net transmitter but contributes relatively lower skewness spillovers than BTC over most of the sample period. This is consistent with the average connectedness results. Notably, BAT is the largest constant net receiver, followed by LTC and Dash. Before November 2020, EOS mainly acts as the net receiver. Then, it changes to a net transmitter between December 2020 and June 2021. After November 2021, EOS transmits increasing net skewness spillover to the system. The range of the dynamic net spillover of Dash and EOS is relatively narrow.

Figure 13 shows the dynamic net realized kurtosis connectedness of each cryptocurrency. We find that BTC and ETH are net transmitters during most of the sample period, which agrees with the average connectedness results. Notably, BTC and ETH are net receivers, while Dash, EOS, and BAT are net transmitters of the kurtosis spillovers before 2020. After February 2020, Dash, EOS, and BAT mainly act as net recipients. This indicates that Dash, EOS, and BAT are more vulnerable to the shocks caused by major financial and economic extreme events. Additionally, before the outbreak of the COVID-19 pandemic, LTC acted as the net receiver and received relatively higher spillovers from other cryptocurrencies. But it becomes the net transmitter after COVID-19 and exhibits a growing trend over time. This finding is supported by Mensi et al. (2021) results that LTC exerts significant spillovers to other cryptocurrencies. Regarding the frequency results, the kurtosis net spillover spreads in the short run. Exceptions that the medium-term horizon dominates the net spillovers can be found in ETH and EOS after March 2021.

The evolution of the dynamic jump net connectedness is shown in Fig. 14. Notably, the dynamic net spillovers of the SJV show several peak values from September to December 2019. This finding is consistent with the total spillover result that the dynamic total SJV connectedness peaked in September 2019. BAT acts as the largest net transmitter during this sample period, followed by Dash, while BTC, LTC, and EOS are all net receivers of the jump connectedness. After early 2020, BTC, ETH, and LTC mainly act as net transmitters while Dash and BAT are net recipients during most of the sample periods. Notably, Dash becomes the net transmitter in June 2021.

The dynamic net connectedness of the RHS and RHK is also shown in Appendix Figs. 26 and 27. Similarly, over the sample period, BTC and ETH are the constant net transmitters of realized hyper-skewness spillovers. And both of them peaked following the COVID pandemic. As a result, Dash and EOS are net receivers for the majority of the sample periods. Notably, the dynamic net RHS spillovers of LTC and BAT show opposite movement trends: before April 2020, LTC (BAT) is the net receiver (transmitter); from April 2020 to June 2021, LTC (BAT) is the net transmitter (receiver); and after June 2021, LTC (BAT) becomes the net receiver (transmitter).

In terms of realized hyper-kurtosis connectedness, we discover that BTC and ETH serve as the two persistent spillover net transmitters, while Dash serves as the constant net recipient throughout the sample period. LTC acts as the net receiver until March 2020, when it switches to being the constant net contributor to RHK connectedness. During the majority of the sample period (except the first half of 2021), EOS is the net receiver and receives higher RHK spillovers after June 2021. BAT is the net transmitter before May 2020 and after June 2021, while it is the net receiver from mid-2020 to mid-2021, albeit with a lower magnitude. The frequency connectedness demonstrates that short-term movements mainly dominate the dynamic net RHK spillovers. However, the medium-term factors also matter a lot in the dynamic net RHK spillovers of Dash, LTC, and EOS pairs.

### Dynamic net-pairwise connectedness analysis

To capture the dynamics of the frequency connectedness between Bitcoin and other selected currencies, we further present the dynamic net pairwise connectedness indices of RS, RK, and SJV in Figs. 15, 16, 17, 18, 19 and 20. The dynamic net pairwise connectedness indices of RHS and RHK are also illustrated in Appendix Figs. 28, 29, 30 and 31.^18^

**Fig. 15:**
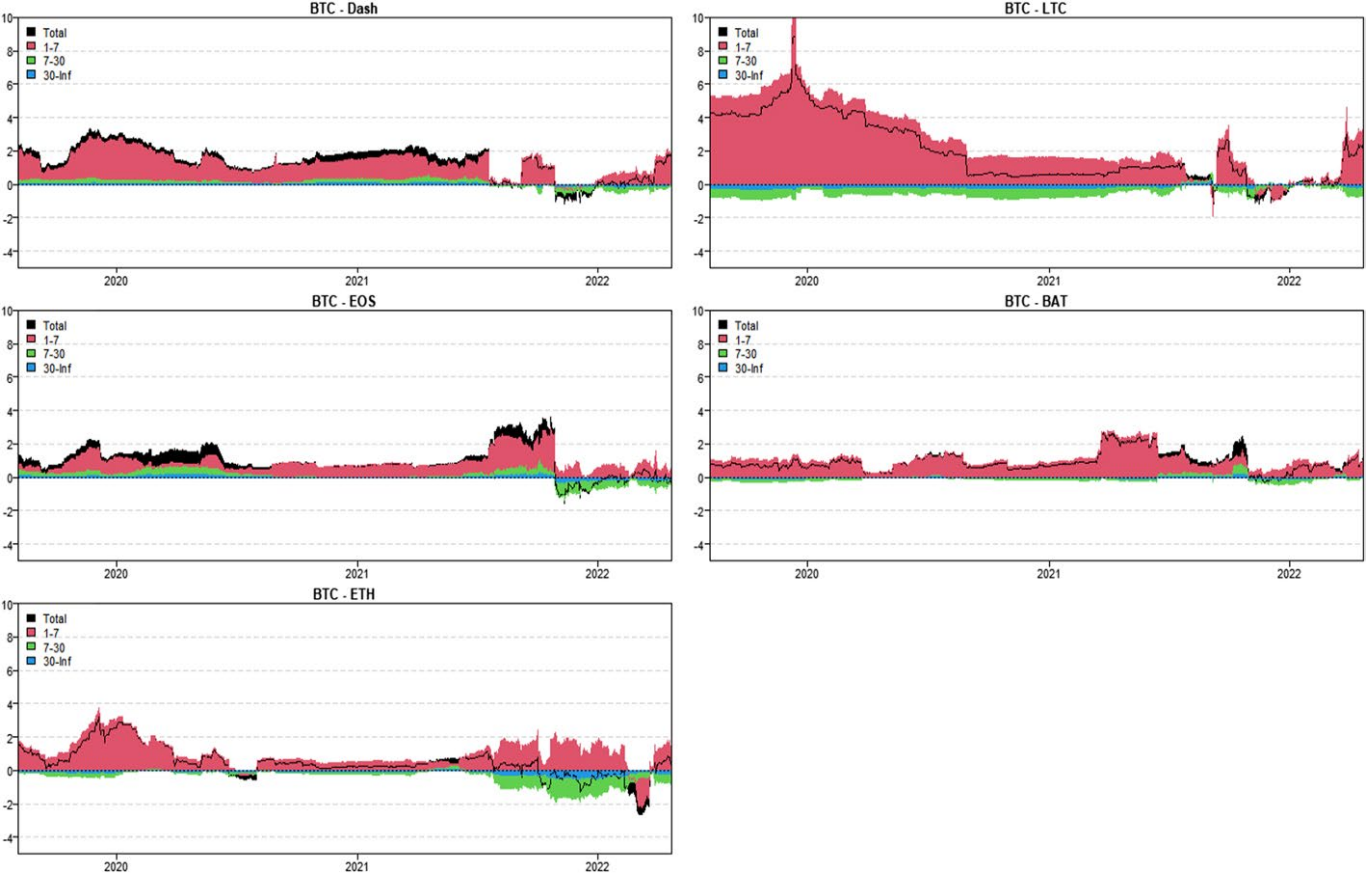
Dynamic net-pairwise connectedness—realized skewness (RS)-BTC

**Fig. 16:**
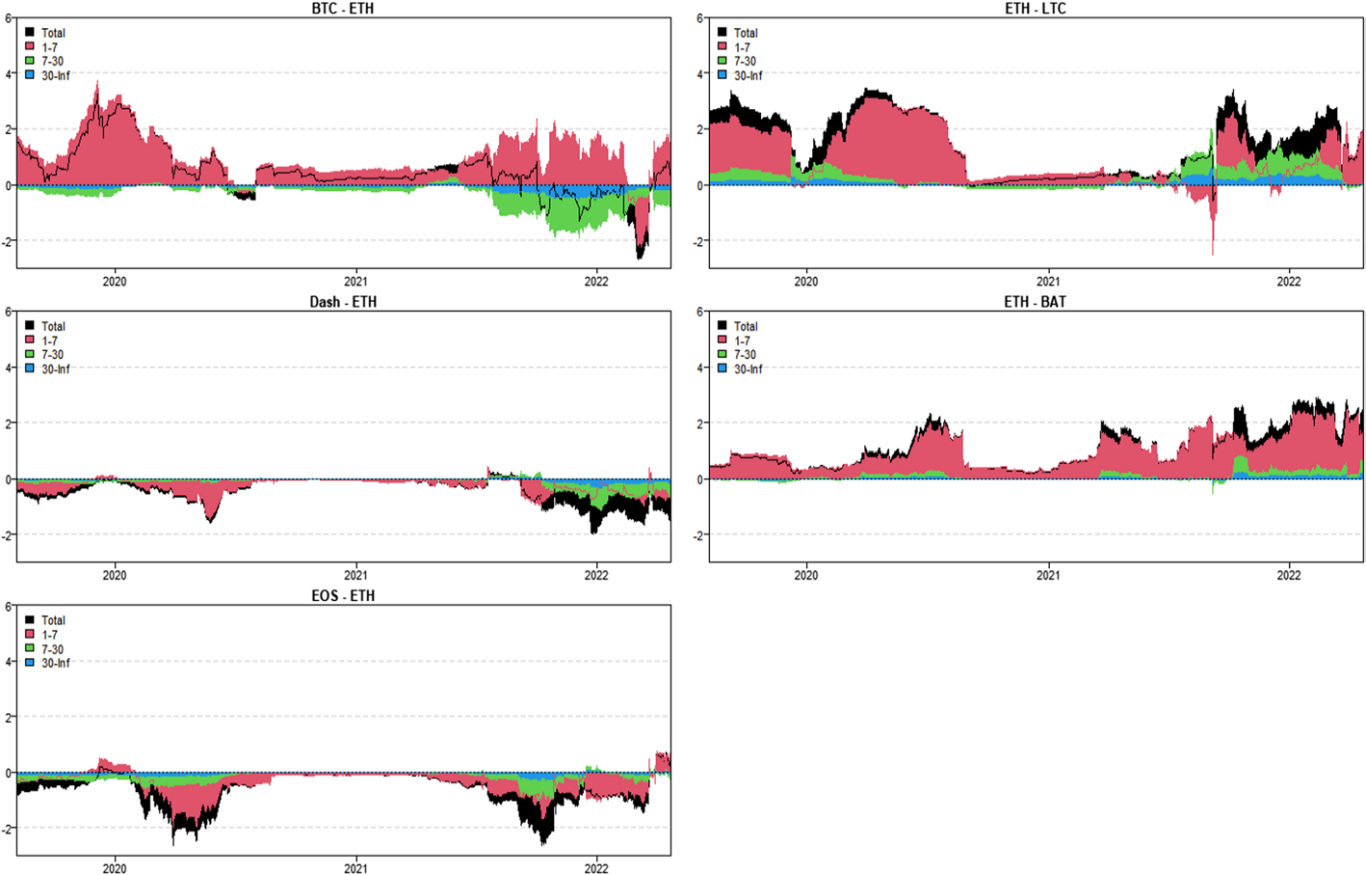
Dynamic net-pairwise connectedness—realized skewness (RS)-ETH

**Fig. 17:**
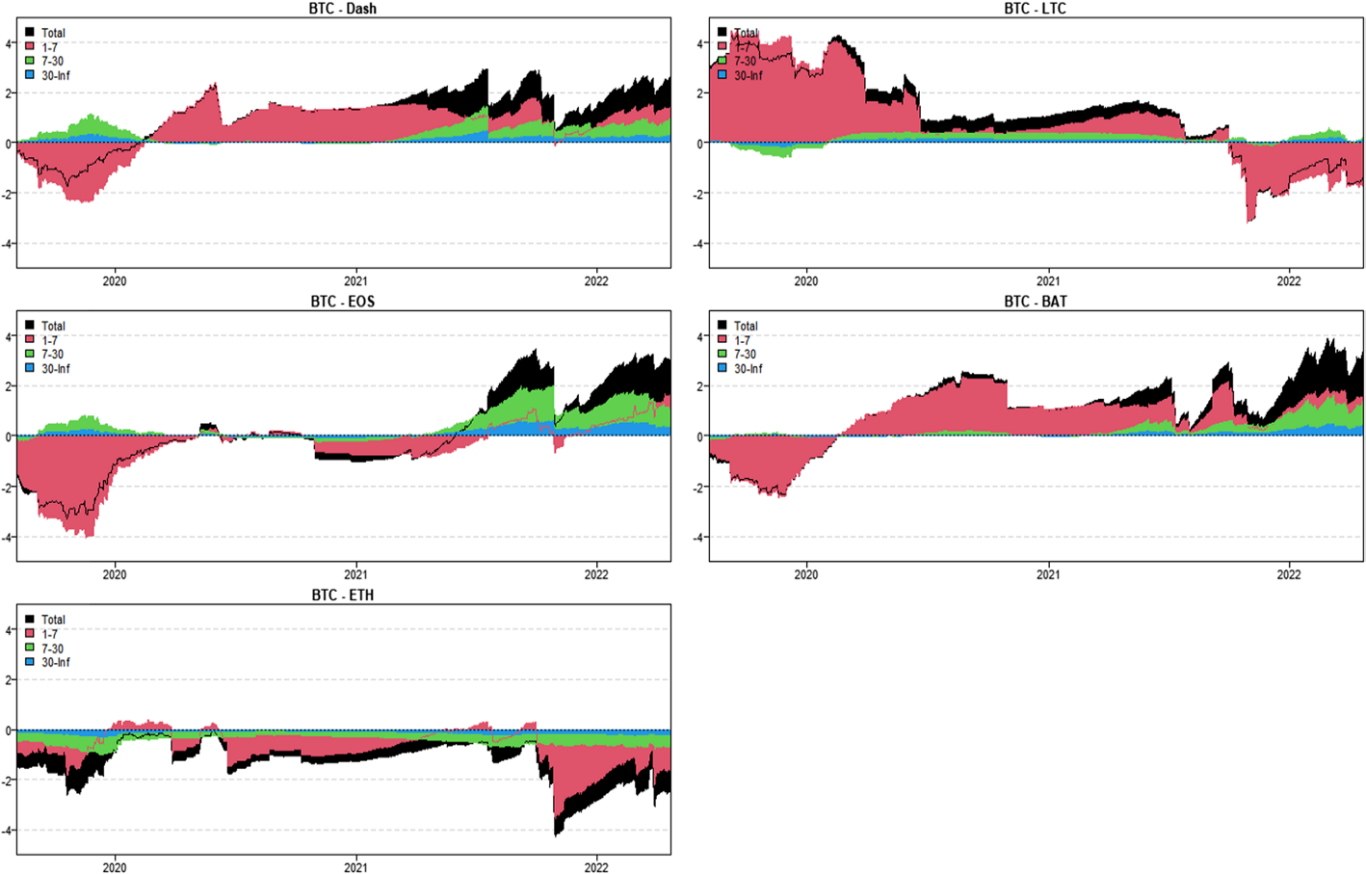
Dynamic net-pairwise connectedness—realized kurtosis (RK)-BTC

**Fig. 18:**
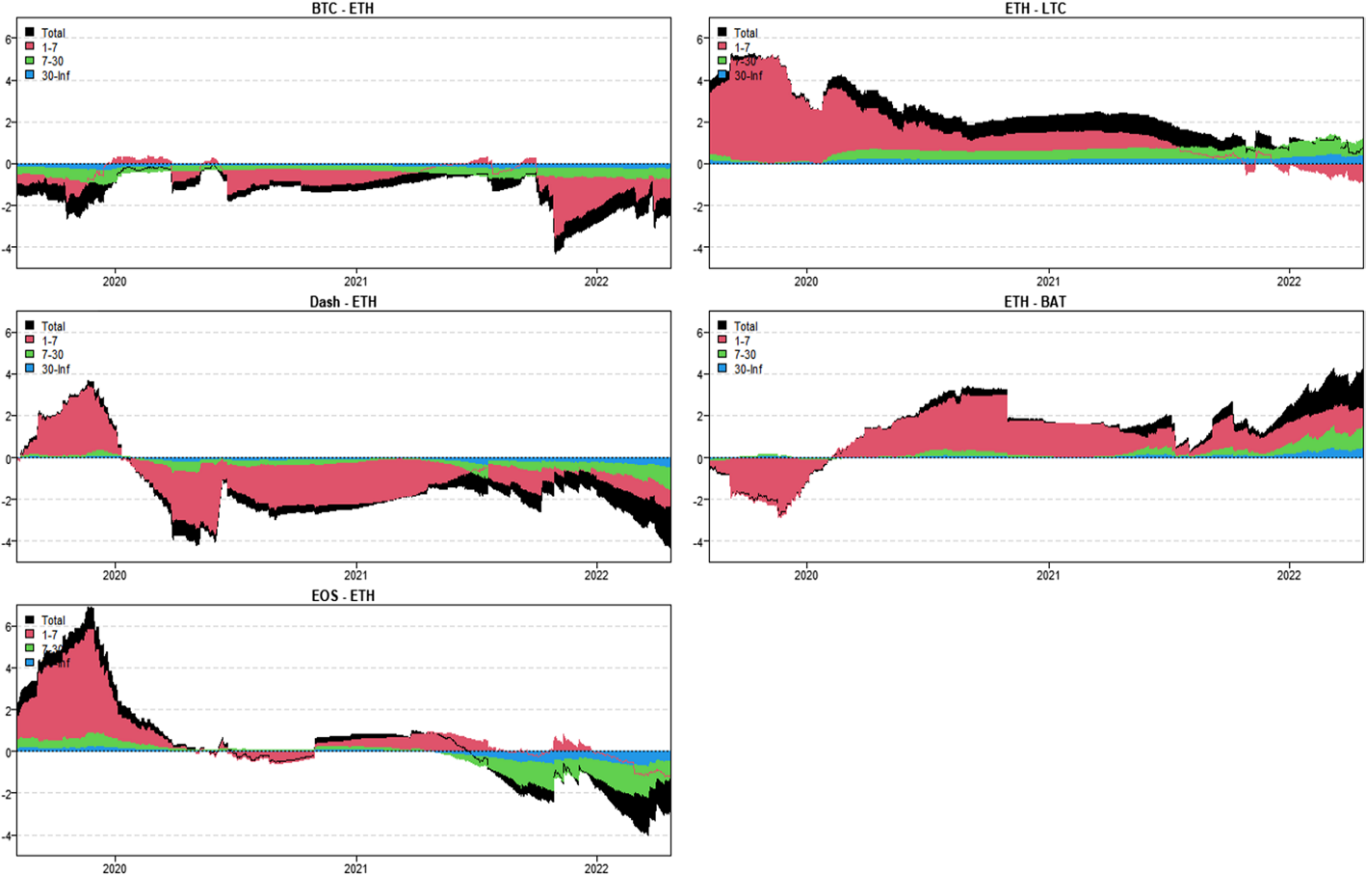
Dynamic net-pairwise connectedness—realized kurtosis (RK)-ETH

**Fig. 19:**
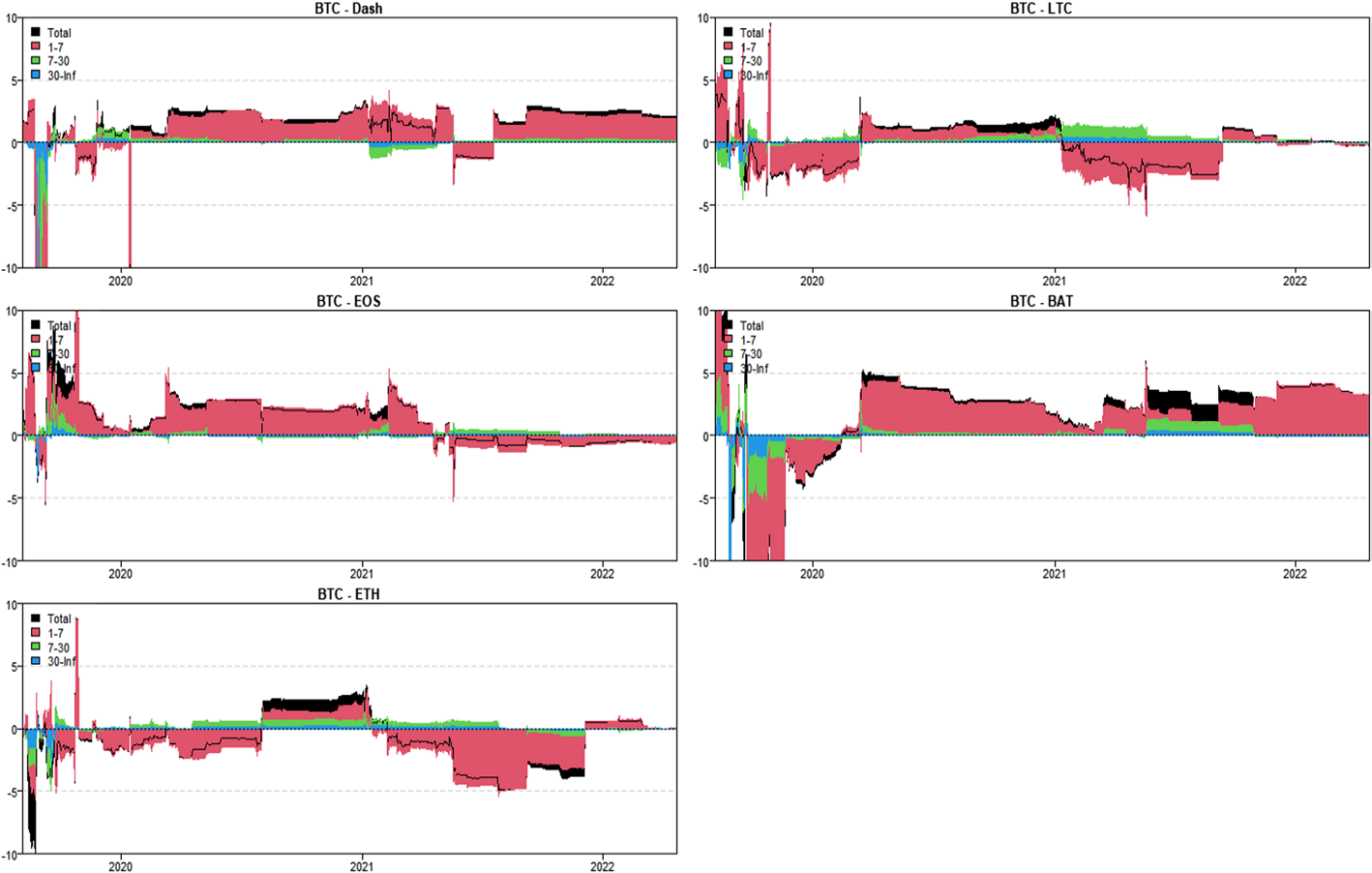
Dynamic net-pairwise connectedness—signed jump variation (SJV)-BTC

**Fig. 20:**
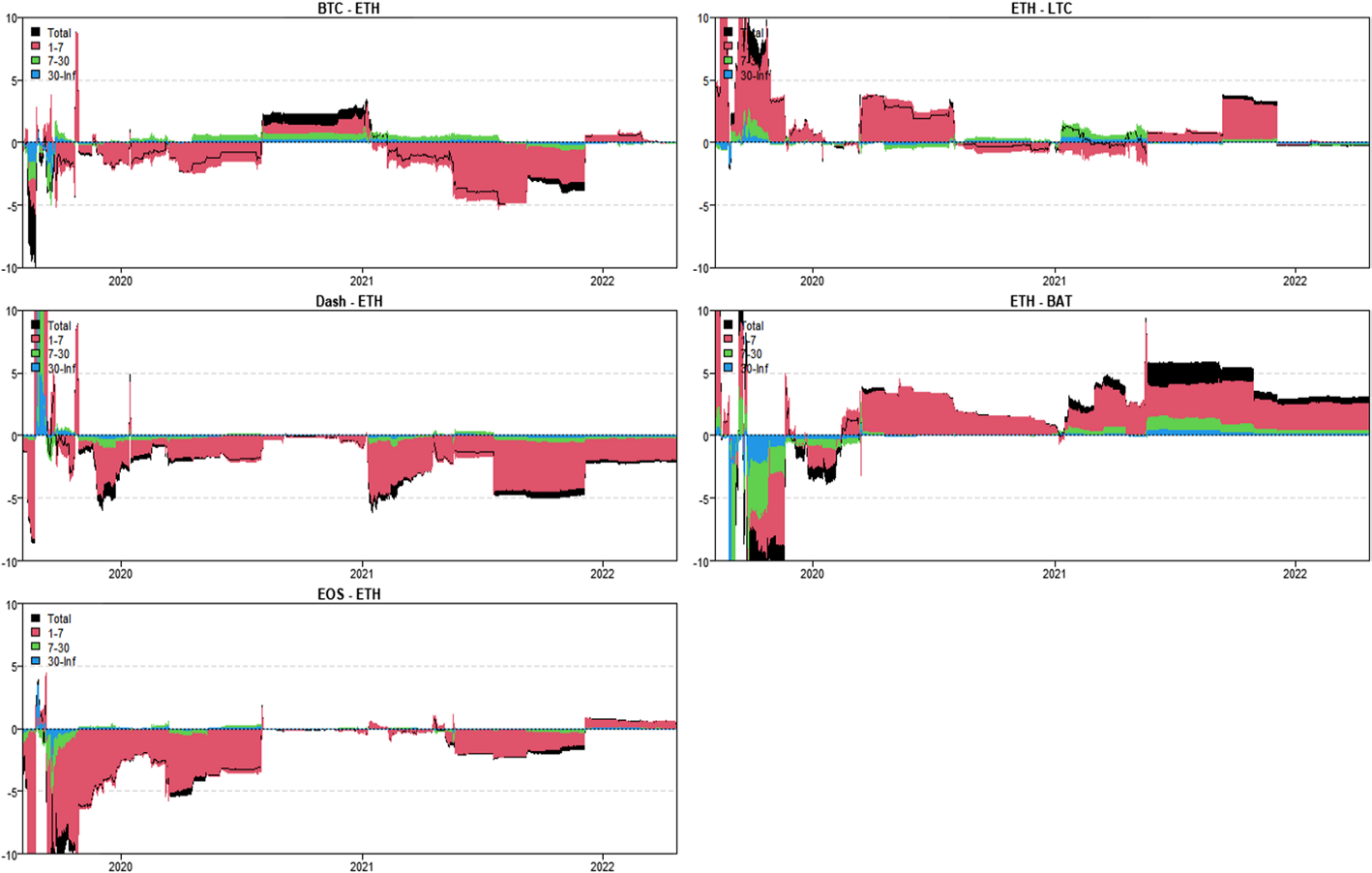
Dynamic net-pairwise connectedness—signed jump variation (SJV)-ETH

Focusing on Fig. 15, we find that the dynamic net pairwise skewness spillovers are mostly positive over the sample period, demonstrating that Bitcoin transmits significant skewness (asymmetric) risk spillovers to other cryptocurrencies, especially Litecoin. The dynamic net pairwise connectedness for the BTC-LTC pair peaks in December 2019 corresponding to the date of the first reported case of COVID-19. Notably, the red regions dominate the dynamic net pairwise spillovers, suggesting that the dynamic net pairwise connectedness is also mainly transmitted in the short run. Remarkably, the dynamic net pairwise spillover transmitted from BTC to LTC in the medium and long term is negative, suggesting that BTC receives considerable skewness spillovers from LTC in the medium and long run. According to Fig. 16, the dynamic net pairwise spillovers transmitted from ETH to other cryptocurrencies (except BTC) are mostly positive over the sample period, implying that ETH contributes significant skewness connectedness to other cryptocurrencies, particularly LTC and BAT. It is also worth noting that ETH transmits significant skewness spillovers to BTC in the medium and long term after mid-2021.

The dynamic net pairwise RK connectedness is depicted in Figs. 17 and 18. Similarly, BTC contributes significant kurtosis spillovers to Dash, LTC, and BAT, demonstrating that extreme shocks in BTC are more easily spread to these cryptocurrencies, increasing the likelihood of extreme risk. Notably, from August 2019 to March 2020, Dash, EOS, and BAT all spread significant kurtosis spillovers to BTC (i.e. before the announcement of the COVID-19 pandemic). Furthermore, after October 2021, LTC contributes significant kurtosis connectedness to BTC. The ETH also transmits relatively higher kurtosis connectedness to BTC, LTC, Dash, and BAT. Before 2020, ETH also receives significant kurtosis spillovers from Dash, BAT, and EOS. For the ETH-EOS pair, the highest value can be found in December 2019 and the medium-term factors dominate the net pairwise spillovers after June 2021. The same phenomenon can be observed for the ETH-LTC pair during this period.

Figures 19 and 20 present the evolution of the dynamic net pairwise SJV connectedness. Several peaks can be found before 2020, which agrees with the dynamic net connectedness result. Generally, BTC transmits notable jump connectedness to Dash, EOS, and BAT. ETH also contributes significant jump spillovers to BTC, LTC, ETH, BAT, and EOS. Notably, BTC also spreads considerable jump spillovers to ETH in the second half of 2020. It’s worth noting that BAT transmits relatively stronger jump spillovers to BTC and ETH in October and December 2019. The dynamic net pairwise jump spillovers are also largely dominated by short-term movements.

Appendix Figs. 28, 29, 30 and 31 show the dynamic net pairwise RHS and RHK connectedness. BTC transmits notable RHS spillovers to other cryptocurrencies (especially LTC) but receives significant connectedness from ETH after March 2020 (the COVID-19 pandemic). BTC spreads relatively stronger RHS spillovers to LTC before September 2020. ETH also contributes notable RHS spillovers to other cryptocurrencies over the sample period. Notably, after the outbreak of the COVID-19 pandemic, the net spillover transmitted from ETH to EOS increased sharply and peaked in mid-2020. Moreover, BTC and ETH transmit significant RHK spillovers to other cryptocurrencies (especially LTC and EOS). The dynamic net RHK spillovers spread from BTC and ETH to LTC, EOS, and BAT all increased remarkably after the outbreak of the COVID-19 pandemic, implying that the net pairwise RHK spillovers are more sensitive to the COVID-19. Finally, the dynamic net-pairwise RHS and RHK spillovers are dominated by a combination of short- and medium-term movements in crypto markets.

### Robustness Check

We further do the robustness check by changing the frequency bands and the VAR lags of the TVP-VAR-based frequency connectedness approach.^19^ To be specific, we reset the frequency bands to the short-term (1–5 days), medium-term (5–22 days), and long-term (more than 22 days). This frequency band is commonly used in the time–frequency spillover analysis (e.g., Pham 2021). The average connectedness results of RV, RS, RK, and SJV are shown in Appendix Tables 11, 12, 13 and 14.^20^ We find that the frequency connectedness of RV is 37.28% (1–5 days), 16.63% (5–22 days), and 13.40% (22–inf days). The frequency connectedness of RS is 33.79% (1–5 days), 5.33% (5–22 days), and 1.87% (22–inf days). The RK connectedness on different frequency bands is 40.13% (short-term), 7.26% (medium-term), and 2.60% (long-term). The SJV frequency connectedness is 40.64% in the short term, 6.73% in the medium term, and 2.74% in the long run. These results are consistent with the frequency connectedness results in Tables 3, 4, 5 and 6. Besides the average connectedness results, we have also depicted the dynamic total connectedness indices at the reselected frequency bands which are presented in Appendix Figs. 32, 33, 34 and 35. We find that those dynamic connectedness indices are highly consistent with the dynamic frequency spillovers illustrated in Figs. 8, 9, 10 and 11.

We also change the lag order of the VAR model and show the dynamic total connectedness indices of RV, RS, RK, and SJV in Appendix Figs. 36, 37, 38 and 39.^21^ We reestimate the TVP-VAR-based frequency connectedness with different VAR lags (1–5 lag orders). Overall, the dynamic total connectedness indices of RV, RS, RK, and SJV with five different lag orders show consistent evolutionary trends and are strongly responsive to specific major events such as the COVID-19 pandemic. All these findings reinforce our earlier empirical results of the moment-based frequency connectedness among cryptocurrencies, namely, our earlier findings are reliable.

## Discussion

In this paper, we comprehensively investigate the higher-order moment comovements and risk spillovers among major cryptocurrencies from both the time- and frequency-domain perspectives. Overall, the time–frequency comovement of Bitcoin and other selected cryptocurrencies varies across frequency bands and sample periods. This finding corroborates a great deal of the previous work by Qiao et al. (2020), Qureshi et al. (2020), and Mensi et al. (2019a). The wavelet coherence results of various realized moments (RV, RS, RK, and SJV) between BTC and other cryptocurrencies are also diverse. The dependence of the same-order moments of cryptocurrencies is both time-dependent and frequency-dependent. This result is consistent with the conclusion of Ahmed (2022). We find that the RV comovement is stronger than the RS, RK, and SJV comovements. For all realized moments, BTC has higher coherence with ETH. This concurs with the findings in Sifat et al. (2019) that there exists higher coherence between BTC and ETH and BTC leads ETH. The influence of BTC is to some extent stronger than Ethereum. Finally, we conclude that higher-order moment time–frequency comovement is most pronounced at the short and medium time scales. This result indicates that tail (extreme) risks among cryptocurrencies tend to comove in the short-term and medium-term. This finding also adds to Ahmed (2022) that cross-kurtosis effects are significant over the entire sample period at short- and medium-range time scales.

Among existing studies highly relevant to our study, Qiao et al. (2020) applied wavelet coherence to investigate the return and volatility comovement between Bitcoin and other cryptocurrencies. They found that Bitcoin has a significant impact on other cryptocurrencies and is ahead of other cryptocurrencies which supports the empirical findings of our study. However, their comovement analysis is confined to return and volatility levels. Moreover, their sample interval spans between April 21, 2014 and October 12, 2019 thus missing the COVID-19 pandemic and the Russia-Ukraine war periods. Qureshi et al. (2020) also analyzed the time–frequency interdependence of cryptocurrency markets and found the short and long-run market integration. The return interdependence oscillates at high frequencies while staying constant at low frequencies. However, their analysis is also limited to the return level. Our study fills this gap by exploring the higher-order moment as well as the jump dependence among cryptocurrencies. The moment-based comovement investigation can provide more targeted and fresh references in the decision-making process for crypto investors with varying investment horizons. Furthermore, looking into higher-order moment comovement effects among cryptocurrencies can shed some new light on time–frequency comovement analysis among other financial markets.

Regarding the moment-based risk connectedness, we find that the connectedness among cryptocurrencies varies with the moments and time scales. It is worth noting that we quantify the connectedness from both the moment and time–frequency perspectives. Specifically, the RV, RS, RK, SJV, RSV, RHS, and RHK spillovers are all dominated by short-term interactions among cryptocurrencies. Besides the volatility spillovers, skewness, kurtosis, and jump spillovers are also pronounced although their magnitudes are lower than volatility. BTC and ETH are two leading net transmitters of risk connectedness to the whole system. We have also captured the significant asymmetric features in volatility spillovers by computing the connectedness of the realized positive and negative semivariance. Notably, as the largest net transmitter, BTC dominates the net-pairwise spillovers of RV and RS connectedness networks while ETH dominates the RK and SJV net-pairwise connectedness among cryptocurrencies. Further, the dynamic connectedness indices of realized moments all vary with time and react remarkably to major events such as the COVID-19 pandemic. The dynamic spillovers among cryptocurrencies are also mainly dominated by short-term movements, that is to say, are mainly transmitted in the short run.

It's worth noting that Hasan et al. (2021) also investigated the realized higher moment connectedness in the cryptocurrency market. They found a moderate, robust, and strong connectedness over volatility, skewness, and kurtosis, respectively. The dynamic connectedness is time-varying and peaked during the COVID-19 pandemic. LTC acts as the leading receiver of RV connectedness. There exist relatively stronger higher-order moment risk spillovers between BTC and ETH. These findings of Hasan et al. (2021) all corroborate our empirical results. More importantly, our study outperforms Hasan et al. (2021) in that we have also analyzed the time–frequency comovements among major cryptocurrencies. Besides the RV, RS, and RK connectedness, we further quantify the RSV, SJV, RHS, and RHK connectedness. We further quantify the moment-based connectedness among cryptocurrencies from both the time- and frequency-domain perspectives.

Our findings also corroborate the results of Hasan et al. (2021), Yi et al. (2018), and Bouri et al. (2021a) that the total connectedness of RV is higher than that of RS and RK. The frequency connectedness results are in line with Kumar et al. (2022) and Mensi et al. (2021) that the total spillovers are mainly transmitted in the short run. We confirm the dominant role of BTC in risk connectedness to other cryptocurrencies which is supported by the works of Polat and Günay (2021), Mensi et al. (2021), Moratis (2021), Wang and Ngene (2020), Ji et al (2019), and Raza et al. (2022). Moreover, our analysis concurs with Polat and Günay (2021), Elsayed et al. (2022), Lahmiri and Bekiros (2020), and Özdemir (2022) that the COVID-19 pandemic has exerted a great impact on the dynamic risk spillovers in cryptocurrency markets. The asymmetric characteristic of the volatility connectedness among cryptocurrencies excavated in this study is supported by the works of Mensi et al. (2019b), Iqbal et al. (2021), Li et al. (2022), Apergis (2022), and Ahn (2022). Our findings also add to the previous works of Gomez-Gonzalez et al. (2022), Bonato et al. (2020), Dai et al. (2021), Bouri et al. (2021a), Ahmed (2022), Gkillas et al. (2022), and Cui et al. (2022) that explored the higher-order moment risk spillovers among financial markets. We have profoundly revealed the higher-moment risk connectedness trajectories among leading cryptocurrencies and identified the major net transmitters and receivers of risk spillovers.

Although our study has drawn rich empirical conclusions, several limitations need to be noted regarding the present study. First, in time–frequency comovement analysis, we have only examined the wavelet coherence between Bitcoin and other cryptocurrencies (Dash, EOS, Ethereum, Litecoin, and Basic Attention Token). This study has not revealed the time–frequency dependence among other cryptocurrencies. Indeed, existing studies like Qiao et al. (2020), Phillips and Gorse (2018), and Sifat et al. (2019) also considered the BTC as a benchmark and analyzed the comovements between Bitcoin and other cryptocurrencies. Second, our sample interval ranges from 5 August 2019 to 23 April 2022 which includes the COVID-19 pandemic and Russian-Ukrainian War. However, our sample period is not long enough to allow us to explore the impacts of major events before August 2019 on risk connectedness among cryptocurrencies. Undeniably, several studies such as Hasan et al. (2021), Kakinaka and Umeno (2022), Maghyereh and Abdoh (2022), Mandaci and Cagli (2022), Özdemir (2022), Raza et al. (2022), Katsiampa et al. (2022), and Chen et al. (2022) also applied the sample interval ranging from 2019 to 2021 or 2022. Additionally, we use 5-min high-frequency trading data in empirical analysis. Hence, the selected 3-year sample interval is sufficient for time–frequency dependence and risk connectedness quantification.

More importantly, our study can be further extended, that is, the risk connectedness results obtained in the aforementioned sections can be used in constructing the minimum connectedness portfolios of cryptocurrencies. Notably, we can also develop the minimum higher-order moment and jump connectedness portfolios based on RV, RS, RK, and SJV pairwise connectedness. By computing the hedging effectiveness, Sharpe ratio, and cumulative returns, we can identify the most efficient crypto portfolio. We can also judge whether considering the high-order moment connectedness will improve the risk management effectiveness of the crypto portfolio. In addition, we can construct the minimum correlation and minimum variance crypto portfolios and compare their risk management effectiveness with the minimum connectedness crypto portfolios.

## Conclusions and implications

Recently, the rapid growth in cryptomarkets has attracted much attention from investors, portfolio managers, market regulators, and governments. The existing literature has extensively examined the complex nexus (linkages, comovement, and risk spillovers) among major cryptocurrencies. However, most of the investigations on comovement and risk connectedness are confined to the first and second moments of the return distribution. More importantly, most studies have focused on the nexus only in the time domain, ignoring the results in the frequency domain. To fill this gap, we explore, for the first time, the higher-order moment comovement and risk connectedness among major cryptocurrencies from both the time- and frequency-domain perspectives.

We draw the following conclusions. First, the wavelet coherence results of various realized moments between BTC and other cryptocurrencies are diverse. The time–frequency comovements of the same-order moments are both time-dependent and frequency-dependent. In general, the time–frequency comovement of RV between BTC and other cryptocurrencies is stronger than the comovement of realized skewness, realized kurtosis, and signed jump variation. BTC exhibits relatively higher comovement with ETH at all realized moments. BTC also exhibits positive interactions with other cryptocurrencies and leads them across the frequency bands and most of the sample periods. Moreover, the RV comovement is stronger at the frequency band of 4–128 days, whereas the RS, RK, and SJV comovements are stronger on the time scale of 4–64 days.

Second, there is a mixed magnitude of risk connectedness at various realized moments, implying that higher-order moments and jumps among cryptocurrencies have heterogeneous spillover effects. The spillovers of higher-order moments and jumps are also notable, although the volatility spillovers outweigh them. This emphasizes the importance of quantifying and tracking higher-order moment and jump risk spillovers among cryptocurrencies. The total connectedness of bad volatility is stronger than good volatility, indicating an asymmetry in risk spillovers among cryptocurrencies.

Third, BTC acts as the largest net transmitter of volatility and skewness connectedness while ETH is the largest net contributor of kurtosis and jump spillovers. Both BTC and ETH are major risk net-spreaders, transmitting significant net connectedness to other cryptocurrencies at various realized moments. Furthermore, the risk spillovers of various realized moments among cryptocurrencies are primarily transmitted in the short run (1–7 days), namely, the short-term interactions among cryptocurrencies dominate the risk connectedness.

Finally, the dynamic total, net, and net-pairwise connectedness indices of all realized moments are time-varying and have been significantly affected to some extent by the COVID-19 pandemic. The short-term movements mainly dominate the dynamic spillovers, although the medium-term factors play an important role in dynamic net and net-pairwise connectedness during some specific sample periods.

Our findings can offer great practical implications for crypto investors, regulators, and policymakers in their decision-making process of portfolio optimization and systemic risk management. First, both the investors and market regulators should stay high alert to not only the volatility spillovers but also the higher-order moment and jump comovement and risk spillovers in cryptocurrencies. The higher-order moment comovement and risk connectedness results reveal how cryptocurrencies interact through asymmetry (or crash risk) and fat-tail (or extreme) risks. Ignoring the comovement and connectedness among cryptocurrencies that can emerge through realized higher-order moments may lead to sub-optimal portfolio strategies. Policymakers and regulators need to deliberately and comprehensively evaluate the risk profile of the cryptocurrency market and formulate policies accordingly. Therefore, considering the higher-order moment dependence and risk connectedness can help policymakers and regulators further enhance their comprehension of risk-generating as well as transmission mechanisms among cryptocurrencies and formulate more scientific crypto risk control policies and measures.

Second, investors and portfolio managers can take into account the higher-order moment risk connectedness in their portfolio construction process. Specifically, investors and portfolio managers can construct bivariate portfolios, namely hedging and optimal-weighted strategies. Moreover, they can also develop multivariate crypto portfolios. Besides the conventional minimum correlation and variance portfolios, they can further construct the minimum RV, RS, RK, and SJV connectedness crypto portfolios which are based on the pairwise connectedness obtained in “Realized moment connectedness results” section. By computing the hedging effectiveness, Sharpe ratio, and cumulative returns, they can identify the most efficient crypto portfolio. Furthermore, investors and portfolio managers should develop higher-moment (i.e., Mean–Variance-Skewness-Kurtosis) crypto portfolio strategies beyond traditional Markowitz’s Mean–Variance portfolio framework to further improve the effectiveness of portfolio risk management. In sum, high-order moment risks should be seriously considered in portfolio construction.

Third, given that BTC and ETH are two leading net transmitters of RV, RS, RK, and SJV connectedness to other cryptocurrencies, crypto investors, regulators, and policymakers should pay enough attention to the risk connectedness transmitted by Bitcoin and Ethereum, especially during the COVID-19 pandemic. Fourth, in light of that risk connectedness among cryptocurrencies is mainly transmitted in the short term (1–7 days), crypto investors and portfolio managers with shorter investment horizons should stay alert to the risk transmissions among cryptocurrencies. Regulators should pay more attention to the risk transmissions among cryptocurrencies in the short run. Considering the time-variant characteristic of the dynamic risk connectedness, investors and portfolio managers are supposed to adjust their portfolio strategies frequently. Regulators and policymakers should reevaluate their risk management policies and measures dynamically according to the real-time status. Furthermore, policymakers should develop and implement more targeted regulations according to the moment- and frequency-based comovement and risk spillover conditions. Finally, market regulators and risk management agencies are supposed to establish emergency response and disposal mechanisms for major crisis events thus minimizing the negative effects on the cryptocurrency market.

Future research could investigate the higher-order moment portfolio optimizations of cryptocurrencies. Another promising research direction is to investigate the extreme risk connectedness among cryptocurrencies from a time–frequency domain perspective using a combination of the quantile VAR model and the frequency connectedness approach.

## Figures and Tables

**Fig. 21:**
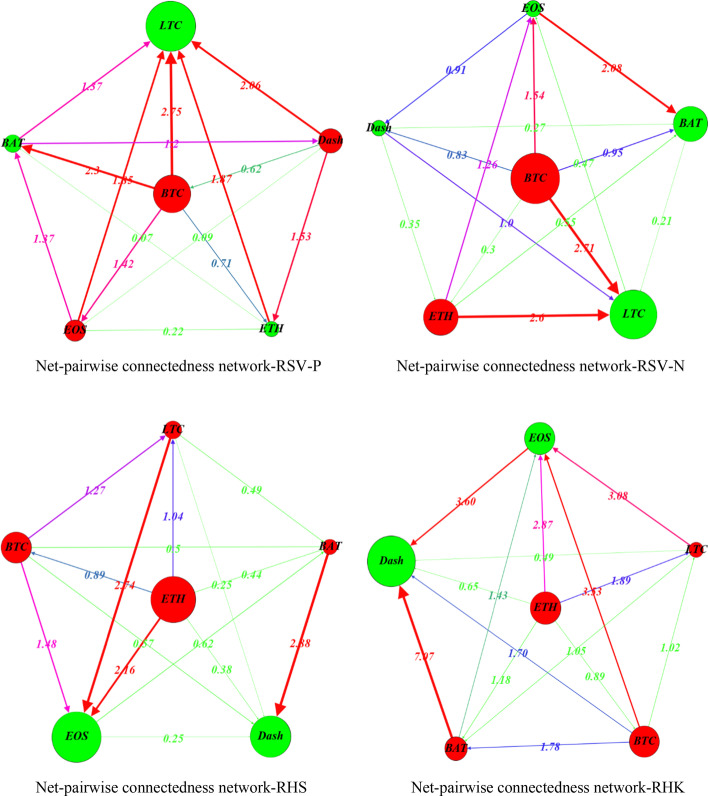
Directional net-pairwise connectedness networks

**Fig. 22:**
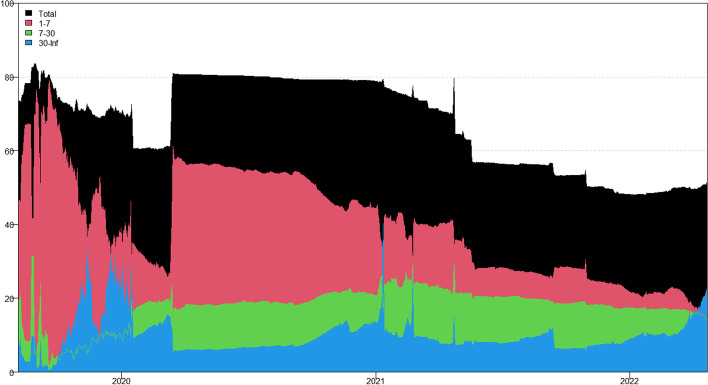
Dynamic total connectedness—realized positive semivariance (RSV-P)

**Fig. 23:**
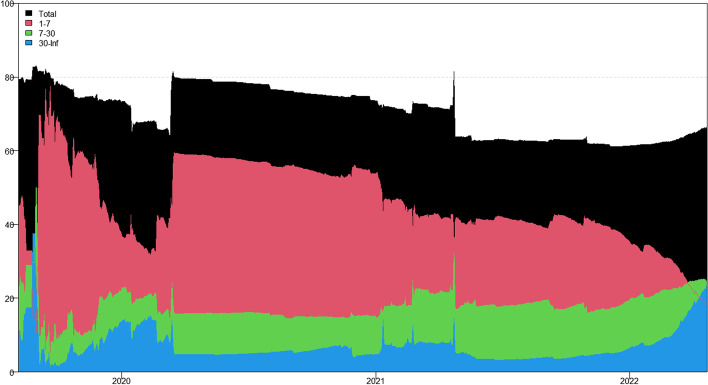
Dynamic total connectedness—realized negative semivariance (RSV-N)

**Fig. 24:**
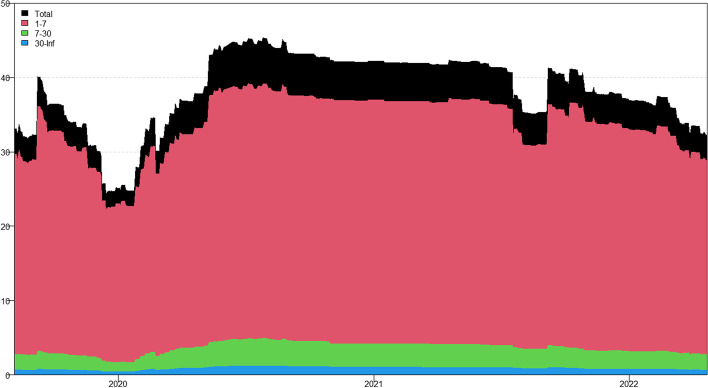
Dynamic total connectedness—realized hyperskewness (RHS)

**Fig. 25:**
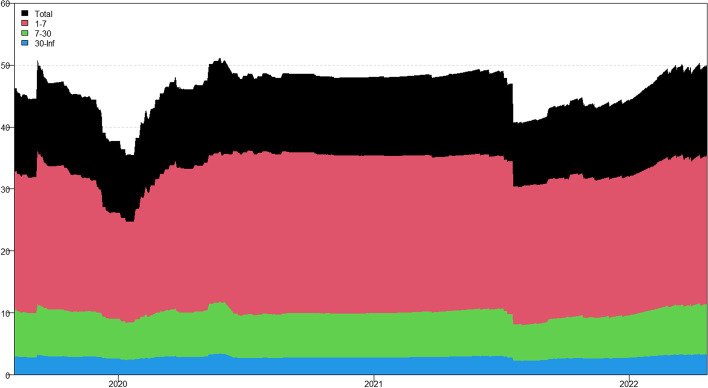
Dynamic total connectedness—realized hyperkurtosis (RHK)

**Fig. 26:**
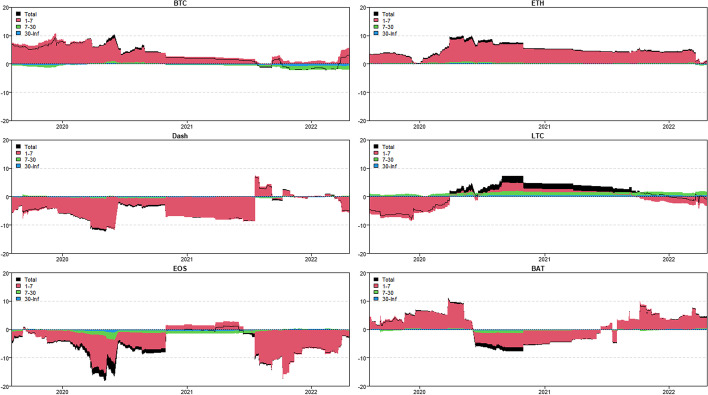
Dynamic net connectedness—realized hyperskewness (RHS)

**Fig. 27:**
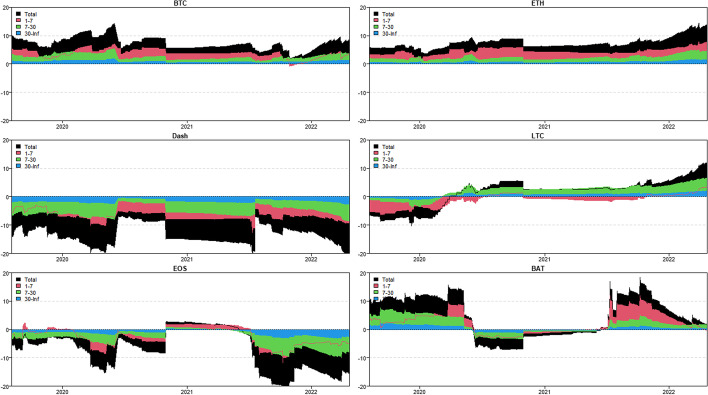
Dynamic net connectedness—realized hyperkurtosis (RHK)

**Fig. 28:**
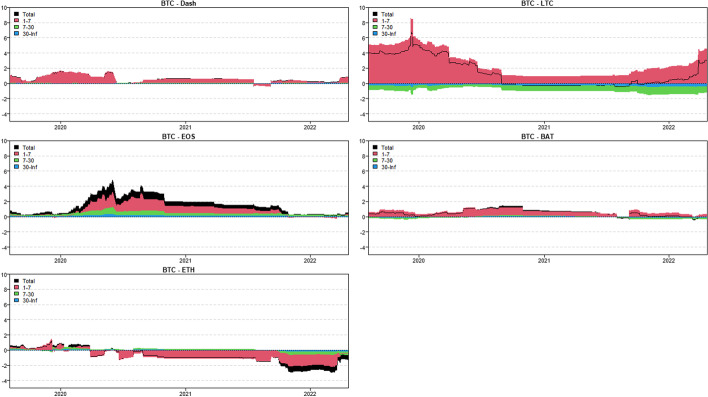
Dynamic net-pairwise connectedness—realized hyperskewness (RHS)-BTC

**Fig. 29:**
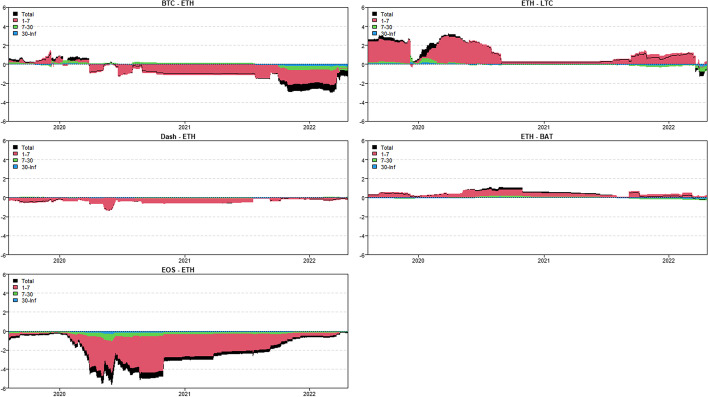
Dynamic net-pairwise connectedness—realized hyperskewness (RHS)-ETH

**Fig. 30:**
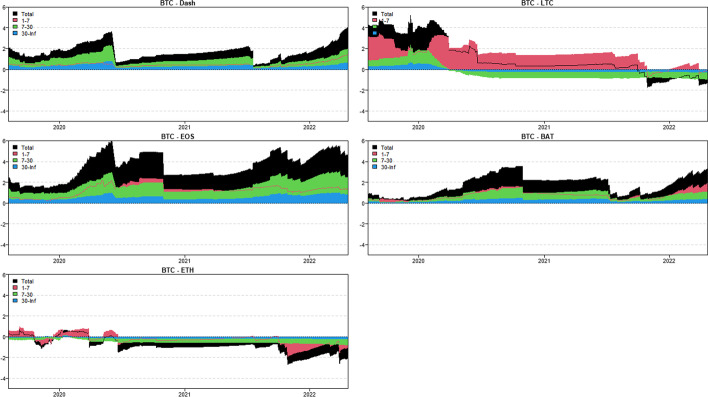
Dynamic net-pairwise connectedness—realized hyperkurtosis (RHK)-BTC

**Fig. 31:**
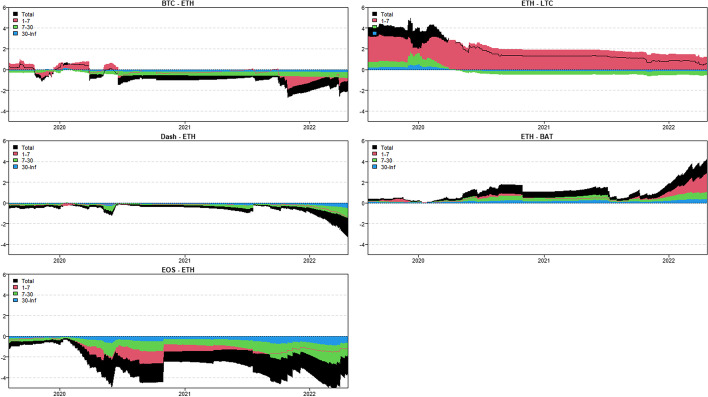
Dynamic net-pairwise connectedness—realized hyperkurtosis (RHK)-ETH

**Fig. 32:**
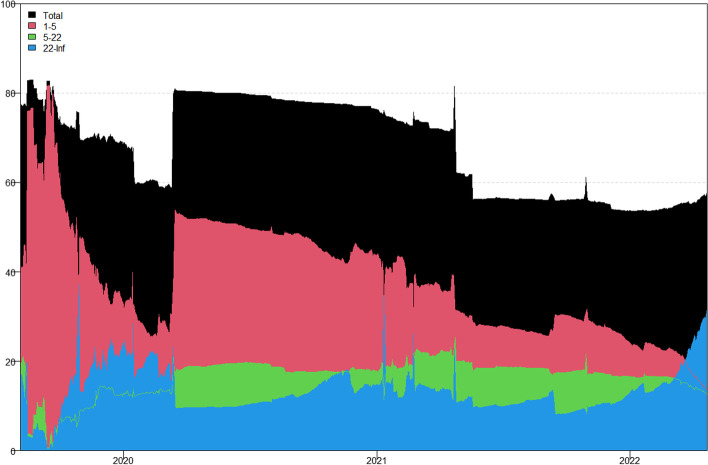
Dynamic total RV connectedness—robustness check

**Fig. 33:**
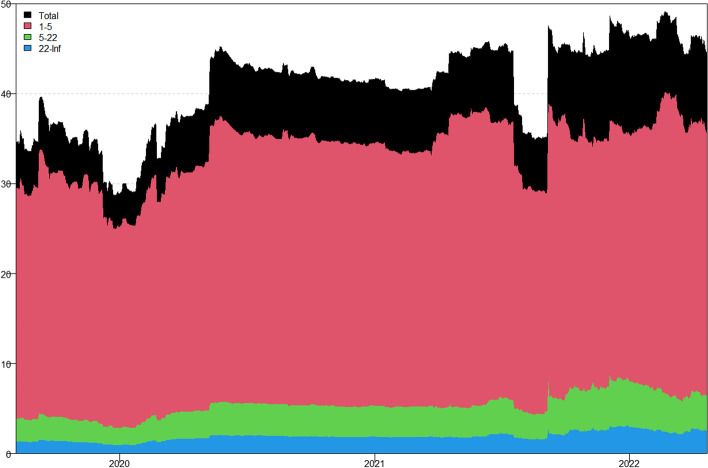
Dynamic total RS connectedness—robustness check

**Fig. 34:**
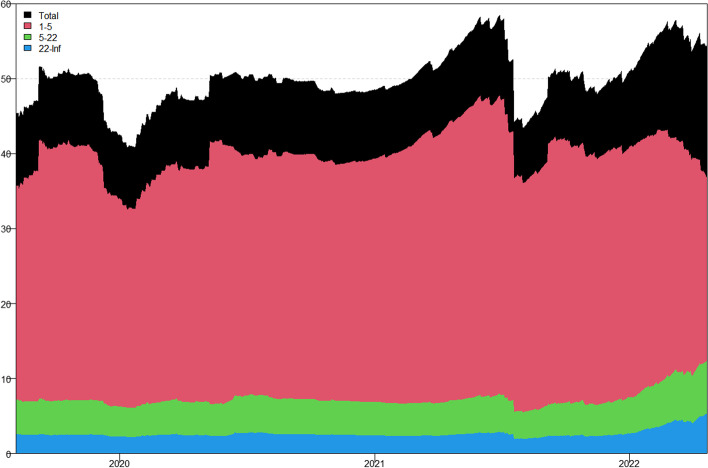
Dynamic total RK connectedness—robustness check

**Fig. 35:**
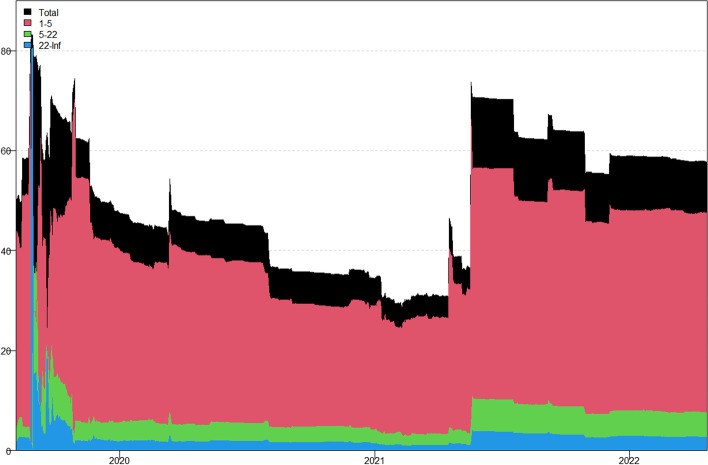
Dynamic total SJV connectedness—robustness check

**Fig. 36:**
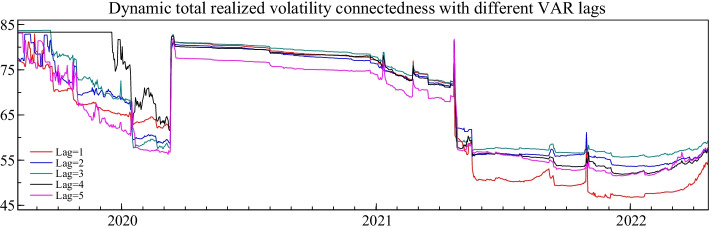
Dynamic total RV connectedness with different VAR lags

**Fig. 37:**
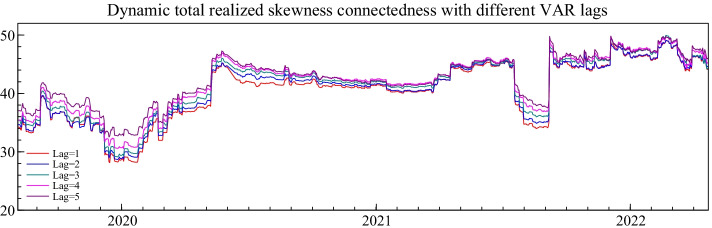
Dynamic total RS connectedness with different VAR lags

**Fig. 38:**
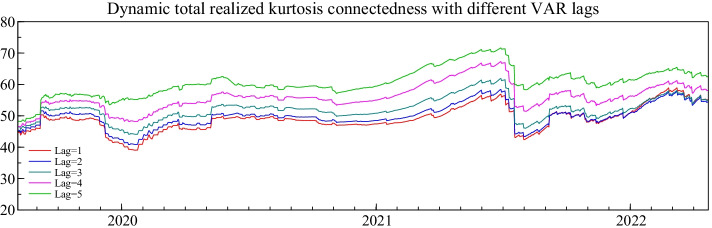
Dynamic total RK connectedness with different VAR lags

**Fig. 39:**
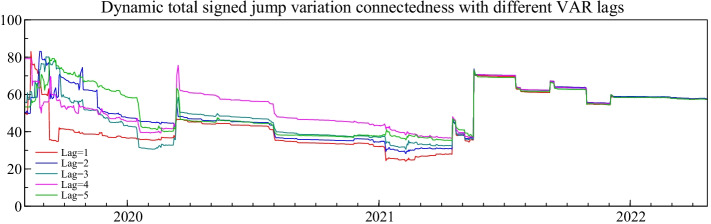
Dynamic total SJV connectedness with different VAR lags

## Data Availability

The datasets used and/or analyzed during the current study are available from the corresponding author upon reasonable request.

## Appendix

See Tables 7, 8, 9, 10, 11, 12, 13 and 14 and Figs. 21, 22, 23, 24, 25, 26, 27, 28, 29, 30, 31, 32, 33, 34, 35, 36, 37, 38 and 39.

**Table 7:** Averaged connectedness table-RSV-P

	BTC	Dash	EOS	ETH	LTC	BAT	FROM
BTC	25.99 (15.49) [7.32] {3.18}	10.00 (5.28) [2.39] {2.33}	11.79 (7.25) [3.07] {1.47}	21.39 (12.62) [6.08] {2.68}	18.02 (10.64) [5.14] {2.24}	12.81 (8.13) [3.24] {1.44}	74.01 (43.92) [19.93] {10.16}
Dash	9.38 (4.06) [2.95] {2.37}	49.13 (23.47) [14.75] {10.91}	14.91 (6.57) [5.09] {3.25}	7.07 (3.30) [2.17] {1.61}	5.53 (2.88) [1.57] {1.07}	13.98 (6.55) [4.57] {2.85}	50.87 (23.37) [16.35] {11.15}
EOS	13.21 (6.95) [3.89] {2.37}	14.82 (6.30) [3.91] {4.62}	33.83 (17.59) [10.96] {5.27}	9.77 (6.02) [2.39] {1.35}	7.71 (4.72) [1.93] {1.06}	20.67 (11.25) [6.25] {3.16}	66.17 (35.25) [18.37] {12.56}
ETH	22.10 (13.60) [5.93] {2.56}	8.60 (5.09) [1.95] {1.56}	9.99 (6.65) [2.41] {0.92}	28.51 (17.18) [7.76] {3.57}	20.48 (11.83) [5.82] {2.82}	10.33 (7.29) [2.23] {0.81}	71.49 (44.47) [18.34] {8.68}
LTC	20.77 (12.84) [5.29] {2.64}	7.59 (5.46) [1.48] {0.66}	9.56 (7.29) [1.74] {0.53}	22.35 (12.84) [6.04] {3.47}	30.62 (18.08) [8.18] {4.36}	9.11 (7.05) [1.58] {0.49}	69.38 (45.47) [16.13] {7.79}
BAT	15.11 (9.09) [4.07] {1.95}	12.78 (7.01) [3.56] {2.21}	22.04 (12.15) [6.85] {3.05}	10.40 (6.80) [2.46] {1.14}	7.74 (5.07) [1.84] {0.83}	31.93 (19.20) [9.15] {3.58}	68.07 (40.12) [18.77] {9.18}
TO	80.56 (46.55) [22.13] {11.88}	53.79 (29.14) [13.28] {11.37}	68.29 (39.91) [19.16] {9.22}	70.99 (41.58) [19.15] {10.26}	59.47 (35.14) [16.30] {8.02}	66.91 (40.27) [17.88] {8.76}	TCI
NET	6.55 (2.63) [2.20] {1.72}	2.92 (5.77) [− 3.07] {0.23}	2.12 (4.67) [0.78] {− 3.33}	− 0.51 (− 2.88) [0.80] {1.58}	− 9.92 (− 10.32) [0.18] {0.23}	− 1.17 (0.15) [− 0.90] {− 0.42}	66.67 (38.77) [17.98] {9.92}

See Table 3

**Table 8:** Averaged connectedness table-RSV-N

	BTC	Dash	EOS	ETH	LTC	BAT	FROM
BTC	24.63 (16.44) [6.20] {1.99}	11.17 (6.52) [3.20] {1.45}	12.82 (7.62) [3.74] {1.46}	20.94 (13.98) [5.36] {1.59}	18.89 (13.00) [4.64] {1.25}	11.54 (7.21) [3.07] {1.26}	75.37 (48.34) [20.03] {7.01}
Dash	12.00 (6.86) [3.09] {2.05}	38.59 (18.59) [12.51] {7.49}	15.66 (8.26) [4.54] {2.87}	10.34 (6.45) [2.70] {1.19}	8.19 (5.03) [2.19] {0.96}	15.22 (8.70) [4.15] {2.36}	61.41 (35.30) [16.67] {9.44}
EOS	14.36 (10.20) [2.99] {1.18}	14.75 (9.92) [3.31] {1.52}	28.73 (20.33) [6.28] {2.12}	13.67 (9.93) [2.79] {0.95}	10.86 (7.85) [2.24] {0.77}	17.62 (12.43) [3.86] {1.33}	71.27 (50.32) [15.19] {5.76}
ETH	21.24 (14.40) [5.27] {1.57}	10.69 (6.33) [3.02] {1.34}	12.41 (7.94) [3.32] {1.14}	25.06 (16.88) [6.27] {1.91}	20.34 (13.92) [5.02] {1.41}	10.27 (7.06) [2.42] {0.78}	74.94 (49.65) [19.05] {6.24}
LTC	21.60 (13.16) [6.10] {2.33}	9.19 (5.17) [2.80] {1.22}	10.39 (6.39) [2.90] {1.09}	22.94 (13.87) [6.52] {2.55}	28.16 (17.57) [7.69] {2.89}	7.73 (5.22) [1.85] {0.66}	71.84 (43.81) [20.17] {7.86}
BAT	12.49 (8.47) [2.95] {1.07}	15.49 (9.85) [4.17] {1.48}	19.70 (12.52) [5.41] {1.77}	10.82 (7.59) [2.43] {0.80}	7.52 (5.26) [1.69] {0.56}	33.98 (21.67) [9.36] {2.96}	66.02 (43.69) [16.65] {5.68}
TO	81.69 (53.10) [20.40] {8.19}	61.29 (37.78) [16.51] {7.00}	70.98 (42.74) [19.91] {8.33}	78.71 (51.82) [19.80] {7.09}	65.80 (45.06) [15.78] {4.96}	62.38 (40.62) [15.36] {6.40}	TCI
NET	6.32 (4.76) [0.37] {1.19}	− 0.12 (2.48) [− 0.16] {− 2.43}	− 0.29 (− 7.58) [4.72] {2.57}	3.77 (2.17) [0.75] {0.85}	− 6.04 (1.24) [− 4.39] {− 2.89}	− 3.64 (− 3.07) [− 1.29] {0.72}	70.14 (45.19) [17.96] {7.00}

See Table 3

**Table 9:** Averaged connectedness table-RHS

	BTC	Dash	EOS	ETH	LTC	BAT	FROM
BTC	50.38 (42.60) [6.25] {1.53}	1.28 (1.22) [0.05] {0.01}	1.31 (1.18) [0.11] {0.03}	26.93 (23.44) [2.79] {0.69}	19.54 (16.32) [2.58] {0.64}	0.55 (0.35) [0.16] {0.04}	49.62 (42.51) [5.70] {1.42}
Dash	1.85 (1.78) [0.07] {0.01}	70.05 (63.68) [5.11] {1.26}	19.27 (18.00) [1.02] {0.25}	0.59 (0.58) [0.01] {0.00}	0.42 (0.41) [0.01] {0.00}	7.82 (7.65) [0.14] {0.03}	29.95 (28.41) [1.25] {0.29}
EOS	2.79 (2.11) [0.54] {0.14}	19.02 (18.01) [0.82] {0.19}	64.81 (57.96) [5.51] {1.34}	2.45 (2.07) [0.31] {0.08}	3.16 (2.66) [0.40] {0.10}	7.77 (7.49) [0.23] {0.05}	35.19 (32.33) [2.30] {0.56}
ETH	26.04 (22.60) [2.76] {0.68}	0.21 (0.17) [0.03] {0.01}	0.29 (0.26) [0.03] {0.01}	48.34 (41.72) [5.31] {1.31}	24.78 (20.55) [3.39] {0.84}	0.34 (0.24) [0.08] {0.02}	51.66 (43.81) [6.30] {1.55}
LTC	20.81 (18.81) [1.62] {0.38}	0.17 (0.12) [0.04] {0.01}	0.42 (0.36) [0.05] {0.01}	25.82 (21.61) [3.37] {0.84}	52.55 (44.88) [6.15] {1.52}	0.23 (0.17) [0.05] {0.01}	47.45 (41.06) [5.14] {1.25}
BAT	1.05 (0.96) [0.07] {0.02}	4.94 (4.67) [0.22] {0.05}	8.39 (7.97) [0.35] {0.07}	0.78 (0.68) [0.08] {0.02}	0.72 (0.59) [0.11] {0.03}	84.11 (71.86) [9.79] {2.46}	15.89 (14.86) [0.84] {0.19}
TO	52.54 (46.25) [5.07] {1.22}	25.62 (24.19) [1.17] {0.27}	29.69 (27.77) [1.56] {0.36}	56.57 (48.37) [6.57] {1.63}	48.62 (40.51) [6.50] {1.61}	16.71 (15.89) [0.66] {0.16}	TCI
NET	2.92 (3.74) [− 0.62] {− 0.20}	− 4.32 (− 4.23) [− 0.08] {− 0.02}	− 5.50 (− 4.56) [− 0.75] {− 0.20}	4.91 (4.56) [0.27] {0.08}	1.17 (− 0.55) [1.36] {0.36}	0.82 (1.03) [− 0.18] {− 0.03}	38.29 (33.83) [3.59] {0.88}

See Table 3

**Table 10:** Averaged connectedness table-RHK

	BTC	Dash	EOS	ETH	LTC	BAT	FROM
BTC	40.60 (31.10) [7.46] {2.04}	0.62 (0.33) [0.22] {0.07}	0.86 (0.59) [0.20] {0.06}	32.66 (25.36) [5.78] {1.52}	24.63 (18.91) [4.52] {1.20}	0.62 (0.35) [0.21] {0.06}	59.40 (45.53) [10.94] {2.92}
Dash	2.32 (0.80) [1.15] {0.37}	63.61 (47.19) [12.87] {3.54}	19.20 (12.18) [5.45] {1.58}	0.84 (0.37) [0.36] {0.12}	0.68 (0.30) [0.29] {0.09}	13.35 (8.67) [3.65] {1.03}	36.39 (22.31) [10.88] {3.20}
EOS	4.39 (1.87) [1.90] {0.61}	15.60 (10.48) [3.97] {1.16}	58.84 (41.70) [13.30] {3.84}	3.43 (1.76) [1.27] {0.40}	3.62 (2.12) [1.14] {0.36}	14.12 (8.64) [4.24] {1.24}	41.16 (24.87) [12.52] {3.77}
ETH	31.77 (25.08) [5.29] {1.39}	0.19 (0.14) [0.04] {0.01}	0.56 (0.45) [0.09] {0.03}	39.84 (31.97) [6.26] {1.61}	27.07 (21.47) [4.46] {1.15}	0.56 (0.40) [0.12] {0.04}	60.16 (47.54) [10.00] {2.61}
LTC	25.65 (20.34) [4.19] {1.12}	0.19 (0.17) [0.02] {0.00}	0.54 (0.48) [0.05] {0.01}	28.96 (23.54) [4.31] {1.11}	44.33 (36.78) [6.01] {1.54}	0.33 (0.25) [0.06] {0.02}	55.67 (44.78) [8.63] {2.27}
BAT	2.40 (1.12) [0.97] {0.31}	6.28 (4.35) [1.49] {0.44}	12.69 (8.60) [3.15] {0.94}	1.74 (0.99) [0.57] {0.18}	1.38 (0.80) [0.44] {0.14}	75.50 (61.65) [10.90] {2.95}	24.50 (15.86) [6.62] {2.01}
TO	66.52 (49.22) [13.50] {3.81}	22.89 (15.47) [5.73] {1.69}	33.86 (22.29) [8.95] {2.62}	67.63 (52.01) [12.29] {3.33}	57.39 (43.60) [10.85] {2.94}	28.98 (18.31) [8.28] {2.39}	TCI
NET	7.12 (3.68) [2.55] {0.88}	− 13.50 (− 6.84) [− 5.15] {− 1.51}	− 7.30 (− 2.58) [− 3.57] {− 1.15}	7.48 (4.47) [2.28] {0.72}	1.72 (− 1.18) [2.23] {0.67}	4.49 (2.44) [1.66] {0.38}	46.21 (33.48) [9.93] {2.80}

See Table 3

**Table 11:** Averaged RV connectedness table-Robustness check

	BTC	Dash	EOS	ETH	LTC	BAT	FROM
BTC	26.72 (14.75) [7.19] {4.79}	8.53 (4.33) [2.03] {2.17}	10.94 (5.72) [2.76] {2.45}	21.19 (12.14) [5.74] {3.31}	20.08 (11.27) [5.65] {3.17}	12.54 (7.57) [2.93] {2.03}	73.28 (41.03) [19.11] {13.13}
Dash	10.05 (3.97) [1.95] {4.12}	44.71 (18.88) [13.13] {12.69}	15.68 (6.44) [4.12] {5.12}	9.09 (4.99) [1.93] {2.17}	6.23 (2.98) [1.32] {1.93}	14.25 (6.79) [3.69] {3.77}	55.29 (25.18) [13.00] {17.11}
EOS	12.08 (6.24) [2.61] {3.23}	13.47 (6.98) [2.96] {3.52}	34.04 (19.00) [8.67] {6.37}	11.13 (7.20) [2.16] {1.77}	8.72 (4.99) [1.88] {1.86}	20.55 (12.07) [4.91] {3.58}	65.96 (37.48) [14.52] {13.95}
ETH	21.24 (12.10) [5.69] {3.45}	9.04 (4.89) [2.18] {1.96}	10.86 (6.30) [2.80] {1.76}	27.99 (16.06) [7.36] {4.57}	20.20 (11.11) [5.69] {3.40}	10.67 (7.08) [2.32] {1.27}	72.01 (41.49) [18.69] {11.83}
LTC	22.26 (11.66) [5.87] {4.72}	6.85 (4.03) [1.62] {1.20}	9.60 (6.27) [2.16] {1.17}	22.68 (11.82) [5.76] {5.10}	29.84 (15.88) [7.83] {6.12}	8.77 (6.03) [1.80] {0.95}	70.16 (39.81) [17.21] {13.14}
BAT	14.91 (8.47) [3.74] {2.70}	12.06 (6.89) [3.12] {2.05}	20.85 (11.23) [5.93] {3.69}	11.22 (7.43) [2.36] {1.43}	8.11 (4.68) [2.08] {1.35}	32.85 (18.83) [9.02] {5.00}	67.15 (38.70) [17.23] {11.23}
TO	80.54 (42.45) [19.87] {18.23}	49.94 (27.13) [11.91] {10.90}	67.93 (35.97) [17.77] {14.19}	75.31 (43.58) [17.95] {13.78}	63.35 (35.02) [16.63] {11.70}	66.78 (39.55) [15.64] {11.60}	TCI
NET	7.27 (1.41) [0.76] {5.09}	− 5.35 (1.95) [− 1.10] {− 6.21}	1.97 (− 1.52) [3.25] {0.24}	3.30 (2.09) [− 0.74] {1.95}	− 6.82 (− 4.79) [− 0.58] {− 1.45}	− 0.37 (0.85) [− 1.59] {0.37}	67.31 (37.28) [16.63] {13.40}

The values in parentheses (), square brackets [], and curly brace {} denote the short-term (1–5 days), medium-term (5–22 days), and long-term (more than 22 days) frequency connectedness results

**Table 12:** Averaged RS connectedness table-Robustness check

	BTC	Dash	EOS	ETH	LTC	BAT	FROM
BTC	47.18 (39.05) [6.03] {2.11}	3.69 (3.05) [0.47] {0.17}	4.65 (3.73) [0.68] {0.24}	25.87 (21.40) [3.29] {1.18}	16.95 (13.38) [2.63] {0.94}	1.66 (1.18) [0.36] {0.13}	52.82 (42.73) [7.42] {2.67}
Dash	5.06 (4.27) [0.60] {0.19}	67.90 (54.64) [9.74] {3.52}	16.99 (14.09) [2.16] {0.74}	2.12 (1.65) [0.34] {0.13}	2.05 (1.68) [0.28] {0.10}	5.89 (5.00) [0.66] {0.23}	32.10 (26.68) [4.03] {1.38}
EOS	5.63 (4.62) [0.75] {0.26}	16.27 (14.12) [1.60] {0.54}	62.02 (50.27) [8.65] {3.10}	3.31 (2.62) [0.50] {0.19}	4.15 (3.31) [0.62] {0.22}	8.62 (6.94) [1.23] {0.45}	37.98 (31.61) [4.71] {1.67}
ETH	26.31 (22.49) [2.86] {0.97}	1.64 (1.39) [0.18] {0.06}	2.66 (2.26) [0.30] {0.10}	47.91 (39.77) [6.01] {2.13}	20.18 (16.06) [3.04] {1.08}	1.29 (1.00) [0.22] {0.08}	52.09 (43.20) [6.60] {2.29}
LTC	18.74 (15.97) [2.07] {0.69}	1.67 (1.25) [0.30] {0.11}	3.41 (2.79) [0.46] {0.15}	21.63 (17.01) [3.37] {1.24}	53.29 (42.72) [7.76] {2.81}	1.27 (0.88) [0.28] {0.11}	46.71 (37.90) [6.50] {2.31}
BAT	2.60 (2.25) [0.26] {0.08}	6.62 (5.49) [0.83] {0.30}	10.55 (9.20) [1.03] {0.32}	2.43 (1.99) [0.32] {0.12}	2.05 (1.70) [0.25] {0.09}	75.76 (61.16) [10.74] {3.86}	24.24 (20.64) [2.69] {0.91}
TO	58.33 (49.60) [6.53] {2.20}	29.89 (25.31) [3.39] {1.19}	38.26 (32.07) [4.63] {1.55}	55.35 (44.67) [7.82] {2.86}	45.38 (36.13) [6.82] {2.43}	18.74 (14.99) [2.75] {0.99}	TCI
NET	5.51 (6.86) [− 0.89] {− 0.46}	− 2.21 (− 1.37) [− 0.64] {− 0.20}	0.27 (0.46) [− 0.08] {− 0.12}	3.26 (1.47) [1.22] {0.57}	− 1.33 (− 1.78) [0.32] {0.13}	− 5.50 (− 5.65) [0.06] {0.08}	40.99 (33.79) [5.33] {1.87}

The values in parentheses (), square brackets [], and curly brace {} denote the short-term (1–5 days), medium-term (5–22 days), and long-term (more than 22 days) frequency connectedness results

**Table 13:** Averaged RK connectedness table-Robustness check

	BTC	Dash	EOS	ETH	LTC	BAT	FROM
BTC	38.03 (31.76) [4.66] {1.61}	2.88 (2.62) [0.20] {0.06}	3.64 (3.32) [0.25] {0.06}	30.86 (26.00) [3.65] {1.20}	22.51 (18.67) [2.89] {0.96}	2.08 (1.92) [0.13] {0.03}	61.97 (52.53) [7.13] {2.31}
Dash	4.09 (3.23) [0.60] {0.26}	58.78 (41.95) [12.14] {4.69}	17.49 (12.13) [3.85] {1.51}	4.23 (3.49) [0.54] {0.20}	3.20 (2.52) [0.49] {0.19}	12.22 (9.13) [2.28] {0.81}	41.22 (30.50) [7.75] {2.96}
EOS	3.81 (2.74) [0.75] {0.32}	19.03 (12.63) [4.61] {1.78}	56.80 (41.06) [11.36] {4.38}	3.28 (2.39) [0.63] {0.26}	3.74 (2.74) [0.70] {0.30}	13.34 (9.42) [2.86] {1.06}	43.20 (29.92) [9.56] {3.72}
ETH	29.46 (25.35) [3.11] {1.00}	2.69 (2.47) [0.18] {0.04}	3.59 (3.15) [0.34] {0.10}	37.32 (31.68) [4.24] {1.40}	24.56 (20.70) [2.92] {0.94}	2.38 (2.21) [0.13] {0.03}	62.68 (53.88) [6.69] {2.11}
LTC	23.64 (19.55) [3.02] {1.07}	2.20 (1.74) [0.34] {0.12}	3.30 (2.64) [0.48] {0.17}	26.94 (22.13) [3.55] {1.25}	42.63 (34.60) [5.95] {2.09}	1.29 (0.99) [0.22] {0.08}	57.37 (47.06) [7.62] {2.69}
BAT	3.20 (2.61) [0.42] {0.17}	12.12 (9.81) [1.70] {0.62}	11.77 (9.13) [1.89] {0.74}	3.79 (3.23) [0.41] {0.15}	2.64 (2.09) [0.39] {0.15}	66.48 (55.37) [8.22] {2.89}	33.52 (26.87) [4.82] {1.83}
TO	64.20 (53.48) [7.91] {2.81}	38.92 (29.26) [7.04] {2.62}	39.78 (30.39) [6.82] {2.57}	69.09 (57.25) [8.79] {3.06}	56.65 (46.72) [7.39] {2.54}	31.31 (23.66) [5.63] {2.02}	TCI
NET	2.24 (0.95) [0.78] {0.51}	− 2.30 (− 1.24) [− 0.72] {− 0.34}	− 3.42 (0.47) [− 2.74] {− 1.15}	6.41 (3.36) [2.10] {0.95}	− 0.72 (− 0.34) [− 0.23] {− 0.15}	− 2.21 (− 3.20) [0.81] {0.18}	49.99 (40.13) [7.26] {2.60}

The values in parentheses (), square brackets [], and curly brace {} denote the short-term (1–5 days), medium-term (5–22 days), and long-term (more than 22 days) frequency connectedness results

**Table 14:** Averaged SJV connectedness table-Robustness check

	BTC	Dash	EOS	ETH	LTC	BAT	FROM
BTC	40.26 (33.89) [4.71] {1.66}	6.21 (4.95) [0.79] {0.47}	9.39 (7.86) [1.13] {0.40}	22.40 (19.17) [2.39] {0.84}	15.64 (13.48) [1.62] {0.55}	6.10 (4.91) [0.77] {0.43}	59.74 (50.36) [6.69] {2.69}
Dash	6.90 (5.72) [0.89] {0.29}	64.85 (52.05) [9.30] {3.50}	10.98 (9.04) [1.44] {0.50}	6.99 (5.75) [0.93] {0.30}	7.11 (5.77) [1.00] {0.35}	3.17 (2.24) [0.60] {0.33}	35.15 (28.51) [4.86] {1.78}
EOS	10.54 (8.78) [1.31] {0.45}	9.57 (7.58) [1.29] {0.69}	45.70 (37.97) [5.72] {2.01}	12.23 (10.24) [1.47] {0.52}	13.91 (11.47) [1.79] {0.64}	8.04 (5.48) [1.70] {0.86}	54.30 (43.56) [7.57] {3.17}
ETH	21.48 (17.96) [2.62] {0.90}	5.62 (4.28) [0.83] {0.52}	10.14 (8.34) [1.32] {0.49}	39.72 (33.39) [4.70] {1.63}	17.43 (14.39) [2.25] {0.79}	5.61 (4.09) [1.00] {0.52}	60.28 (49.05) [8.01] {3.22}
LTC	15.46 (12.78) [1.99] {0.69}	6.78 (5.22) [0.97] {0.59}	13.23 (10.61) [1.92] {0.70}	18.93 (15.64) [2.42] {0.87}	39.86 (32.49) [5.43] {1.94}	5.74 (3.89) [1.20] {0.65}	60.14 (48.14) [8.50] {3.50}
BAT	7.43 (6.15) [0.94] {0.34}	3.91 (2.57) [0.76] {0.58}	8.39 (6.44) [1.40] {0.54}	7.39 (5.95) [1.04] {0.39}	3.99 (3.11) [0.63] {0.25}	68.89 (50.97) [12.88] {5.04}	31.11 (24.23) [4.78] {2.10}
TO	61.82 (51.39) [7.74] {2.68}	32.09 (24.60) [4.64] {2.85}	52.14 (42.29) [7.21] {2.64}	67.93 (56.75) [8.26] {2.92}	58.08 (48.22) [7.29] {2.58}	28.66 (20.60) [5.26] {2.79}	TCI
NET	2.07 (1.03) [1.05] {− 0.01}	− 3.06 (− 3.91) [− 0.23] {1.08}	− 2.16 (− 1.27) [− 0.36] {− 0.53}	7.65 (7.70) [0.25] {− 0.30}	− 2.06 (0.08) [− 1.21] {− 0.92}	− 2.45 (− 3.63) [0.49] {0.69}	50.12 (40.64) [6.73] {2.74}

The values in parentheses (), square brackets [], and curly brace {} denote the short-term (1–5 days), medium-term (5–22 days), and long-term (more than 22 days) frequency connectedness results

## Footnotes

1 https://www.statista.com/statistics/377382/bitcoin-market-capitalization/.

2 The trading data are recorded on 16 May 2022.

3 https://www.investing.com/crypto/currencies.

4 The expressions ‘spillovers’ and ‘connectedness’ are replaceable in this paper.

5 The herding behavior occurs when investors mimic other investors' behaviors.

6 We thank an anonymous reviewer for pointing this out. If the length of the rolling window is 200 days, it means that the spillover effect results in the first 200-day sample interval are lost.

7 According to Khademalomoom et al. (2019) and Ahmed and Mafrachi (2021), hyper-skewness, the fifth-order moment, is a reflection of the asymmetric sensitivity of the fourth-order moment. High (low) hyper-skewness indicates a small (large) change in the mode (shoulders) of a distribution. Hyper-kurtosis, the sixth-order moment, measures the degree of tailedness and peakedness of a distribution.

8 The Morlet wavelet is selected in many comovement studies such as Qiao et al. (2020), Vacha and Barunik (2012), Ahmed and Sleem (2022), Singh et al. (2019), Mensi et al. (2019a), Celeste et al. (2020), and Qureshi et al. (2020). Aguiar-Conraria et al. (2008) asserted that the Morlet wavelet has optimal joint time–frequency concentration. Vacha and Barunik (2012) pointed out that the Morlet wavelet has both real and imaginary parts, thus allowing us to explore both amplitude and phase.

9 Following Hasan et al. (2021), Petukhina et al. (2021), Yarovaya and Zięba (2022), we choose 5-min frequency data of cryptocurrencies.

10 We thank the three reviewers for pointing this out. Our motivation for choosing the starting date of the sample is based on the data availability on intraday 5-min high-frequency data for cryptocurrencies. Indeed, the starting date of the sample roughly six months before the COVID-19 pandemic is reasonable to merge, and so to compare the higher moment comovement and risk connectedness among cryptocurrencies during two different economic situations, namely during a relatively stable economic period (August 2019 to March 2020) and during the COVID-19 pandemic and the Russian-Ukrainian war.

11 Qiao et al. (2020) and Mensi et al. (2019a) also analyzed the time–frequency comovement between BTC and other cryptocurrencies.

12 The market cap value was recorded on 6 May 2022. The original data can be found at https://www.investing.com/crypto/bitcoin.

13 The financial asset price and the return may have instantaneous large fluctuations on some nearly continuous time nodes, and this price mutation phenomenon is called “jump”. In this paper, we examine the signed jump variation (SJV) spillovers among major cryptocurrencies.

14 As Kumar et al. (2022) asserted, unlike traditional financial assets, crypto markets operate on a 24/7 basis. Thus, a 7-day period corresponds to a trading week while a 30-day period corresponds to a trading month.

15 For simplicity, here we only present the directional net-pairwise networks of the time-domain connectedness results. The frequency-domain net-pairwise connectedness networks are available upon request.

16 https://www.deep-secure.com/news-and-events/221-crypto-news-september.php.

17 In this paper, good volatility is measured by realized positive semivariance (RSVP) presented in Eq. (3) while bad volatility is measured by realized negative semivariance (RSVN) shown in Eq. (4). According to Barndorff-Nielsen et al. (2008), bad volatility (negative semivariance) can be also used to represent the downside risk in financial markets.

18 Given that BTC and ETH are two major connectedness net transmitters, here we just present the dynamic net connectedness indices between BTC, ETH and other cryptocurrencies. Other net pairwise connectedness results are also available upon request.

19 We are very grateful to an anonymous reviewer for offering this valuable suggestion.

20 For lack of space, we only show the robustness check results of RV, RS, RK, and SJV connectedness results.

21 For lack of space, we only show the dynamic connectedness indices with different VAR lags in the time domain.
